# Whole-genome sequence association study identifies cyclin dependent kinase 8 as a key gene for the number of mummified piglets

**DOI:** 10.5713/ab.22.0115

**Published:** 2022-09-07

**Authors:** Pingxian Wu, Dejuan Chen, Kai Wang, Shujie Wang, Yihui Liu, Anan Jiang, Weihang Xiao, Yanzhi Jiang, Li Zhu, Xu Xu, Xiaotian Qiu, Xuewei Li, Guoqing Tang

**Affiliations:** 1 Chongqing Academy of Animal Sciences, Rongchang 402460, Chongqing, China; 2 Farm Animal Genetic Resources Exploration and Innovation Key Laboratory of Sichuan Province, Sichuan Agricultural University, Chengdu 611130, Sichuan, China; 3 Aks Vocational and Technical College, Aksu, 843000, Xinjiang, China; 4 Sichuan Animal Husbandry Station, Chengdu, 610041, Sichuan, China; 5 College of Life Science, Sichuan Agricultural University, Yaan 625014, Sichuan, China; 6 National Animal Husbandry Service, Beijing, 100125, Beijing, China

**Keywords:** Cyclin Dependent Kinase 8 (*CDK8*), Genotype Imputation, Mummified Piglets, Ovarian Granulosa Cells, TGF-β/SMAD Signaling

## Abstract

**Objective:**

Pigs, an ideal biomedical model for human diseases, suffer from about 50% early embryonic and fetal death, a major cause of fertility loss worldwide. However, identifying the causal variant remains a huge challenge. This study aimed to detect single nucleotide polymorphisms (SNPs) and candidate genes for the number of mummified (NM) piglets using the imputed whole-genome sequence (WGS) and validate the potential candidate genes.

**Methods:**

The imputed WGS was introduced from genotyping-by-sequencing (GBS) using a multi-breed reference population. We performed genome-wide association studies (GWAS) for NM piglets at birth from a Landrace pig populatiGWAS peak located on SSC11: 0.10 to 7.11 Mbp (Top SNP, SSC11:1,889,658 bp; p = 9.98E-13) was identified in cyclin dependent kinase on. A total of 300 Landrace pigs were genotyped by GBS. The whole-genome variants were imputed, and 4,252,858 SNPs were obtained. Various molecular experiments were conducted to determine how the genes affected NM in pigs.

**Results:**

A strong GWAS peak located on SSC11: 0.10 to 7.11 Mbp (Top SNP, SSC11:1,889,658 bp; p = 9.98E-13) was identified in cyclin dependent kinase 8 (*CDK8*) gene, which plays a crucial role in embryonic retardation and lethality. Based on the molecular experiments, we found that Y-box binding protein 1 (*YBX1*) was a crucial transcription factor for *CDK8*, which mediated the effect of *CDK8* in the proliferation of porcine ovarian granulosa cells via transforming growth factor beta/small mother against decapentaplegic signaling pathway, and, as a consequence, affected embryo quality, indicating that this pathway may be contributing to mummified fetal in pigs.

**Conclusion:**

A powerful imputation-based association study was performed to identify genes associated with NM in pigs. *CDK8* was suggested as a functional gene for the proliferation of porcine ovarian granulosa cells, but further studies are required to determine causative mutations and the effect of loci on NM in pigs.

## INTRODUCTION

The number of mummified (NM) pigs at birth is a polygenetic trait that causes a substantial loss of fertility. In the modern industry, pig breeding aims to increase the total number of births and the litter size at weaning. Pig reproductive performance has improved noticeably in recent years. However, NM results in a significant loss of piglets in pig breeding programs. Minimum NM is encouraged to reduce the loss of fertility in pigs. Although some studies investigated the genetic architecture of early fetal death in pigs [1,2], no causative variant has been unequivocally identified. Understanding the genetic mechanisms affecting embryonic development is required to reduce NM and increase pig fertility. Currently, our understanding of genetic architecture for NM is negligible. Thus, identifying causal variants and understanding the genetic mechanism of NM will provide a promising selection strategy for pig breeding programs.

The reproductive trait, which is a polygenetic character, is elusive. The genetic architecture of pigs has been successfully explored using genome-wide association studies (GWAS) of the reproductive trait. Several studies have attempted to identify quantitative trait loci (QTLs) and promising candidate genes for fetal death in pigs [1,2]. NM is one of the most important components of prenatal fetal death. A total of 95 QTLs (Pig QTLdb, https://www.animalgenome.org/cgibin/QTLdb/SS/index) in pigs contributed to significant genetic variants for NM. The power of complex trait GWAS was significantly reduced by the low density of single nucleotide polymorphisms (SNPs) panel, which contained limited genetic variation [3]. As this data is expected to contain most of the genetic variants, whole-genome sequencing (WGS) enhances the detection of causal loci [4]. Recently, genotype imputation has been successfully implemented to pursue WGS analysis. Using a reference WGS is a highly desirable method to exploit an imputed WGS for a large-scale target population. Imputation has been effectively implemented in various populations, and has provided many novel genetic variants and QTLs for complex traits. Therefore, using imputed WGS can improve the power of GWAS, especially for identifying rare variants in complex traits.

Cyclin dependent kinase 8 (*CDK8*) is a kinase associated with the mediator complex and directly regulates transcription by phosphorylating Ser2 and Ser5 of the carboxy-terminal domain of RNA polymerase II and the general transcription factor transcription factor IIH (TFIIH) [5]. Many studies found that *CDK8* plays a pivotal role in human cancer development and progression [6]. Furthermore, this gene is also crucial for embryonic development. The expression of zebrafish *CDK8* maternally in embryonic development was revealed using northern blotting and whole-mount *in situ* hybridization [7]. *CDK8* deficiency causes embryonic retardation and lethality in mice because of transcriptional deregulation of developmentally critical genes [8], indicating that it is required for early embryonic development. Y-box binding protein 1 (*YBX1*) is an important transcription factor of *CDK8*, which is widely involved in various biological processes, like DNA replication, transcription, and cell proliferation [9]. The down-regulation of *YBX1* reduces cell proliferation and promotes apoptosis in multiple myeloma cells [10]. *YBX1* transcript and protein have been found to play an important role in embryonic development in mouse embryonic stem cells [9]. *YBX1* protein is abundantly expressed during development and is closely associated with translating many genes involved in cellular growth and death. A high expression level of *YBX1* has been detected in early embryos [11]. The disruption of the mouse *YBX1* gene resulted in severe growth retardation and embryonic lethality after embryonic day 13.5 [12]. *YBX1* is involved in cell proliferation, cell survival, and apoptosis and could play a role in embryonic development. Consequently, it is expected that *YBX1*-mediated *CDK8* may play an important role in early embryonic development in pigs. However, no functional molecular investigation in pigs has been reported to date.

A mummified fetus in pigs is a fetal death that occurs before farrowing and is caused by intrauterine growth retardation (IUGR). The prenatal fetus is affected by IUGR in pigs and humans worldwide. IUGR has been found to affect up to 5% to 10% of fetuses in humans. Deciphering the genetic mechanism underlying fetal death in pigs not only provides important variants for pig breeding programs but may also provide an informative indication for IUGR in humans because the pig is an ideal model for studying human diseases. In the present study, we aimed to investigate the genetic mechanism of NM using the following methods stepwise i) identification of important genetic loci that may affect NM in a purebred Landrace pig population using a powerful GWAS based on the imputed WGS; ii) determination of the most promising functional gene in the GWAS; iii) construction of gene expression profiles in different tissues and ovarian granulosa cells; iv) identification of important transcription factor of the target gene; v) study of the transcription factor that regulated target gene affecting the proliferation of porcine ovarian granulosa cells.

## MATERIALS AND METHODS

### Animal care

All experimental procedures were performed in accordance with the Institutional Review Board (IRB14044) and the Institutional Animal Care and Use Committee of the Sichuan Agricultural University under permit number DKY-B2014 0302.

### Animals and phenotype records

A total of 360 pigs (including 320 Landrace and 40 Yorkshire pigs) from the national core pig breeding farm of Sichuan Tianzow Breeding Technology Co., Ltd ( http://www.tianzow.com/areashow.php?id=790, Nanchong, China) were used in this study. All pigs with common genetic background were obtained from Canadian Hylife Company in 2008. The ear tissues from 360 pigs were collected and stored in a 75% alcohol solution.

A total of 300 Landrace pigs with farrowing records were collected from 2012 to 2016. The phenotypic distributions for farrowing intervals were not a standard normal distribution. The normal transformation of phenotypic records was conducted using R software. The statistical results of the phenotypic data are revealed in Table 1.

### Genotyping by sequencing

A total of 300 Landrace pigs with phenotypic records were sequenced using genotyping by sequencing (GBS) technology (Supplementary Table S1). Sequencing was performed on an Illumina HiSeq PE150 instrument, and the clean reads were aligned to the pig reference genome (Suscrofa11.1) using Burrows-Wheeler Aligner (BWA) software version 0.7.15 [13]. Genetic variants were detected by GATK’s HaplotypeCaller as documented in GATK Best Practices [14]. We removed the sites with minor allele frequency (MAF) less than 0.01, a missing rate higher than 0.2, and a mean depth greater than or equal to 3 using VCFtools (version 4.2) [15]. SNPs located on sex chromosomes with no position information were then filtered from this dataset. After quality control, 325,557 SNPs were obtained.

### Whole-genome sequencing

Illumina HiSeq PE150 platform was used to obtain the WGS reference data from 60 pigs (including 20 Landrace and 40 Yorkshire pigs) (Supplementary Table S2). The clean reads were aligned to the pig reference genome (Suscrofa11.1) using BWA version 0.7.15 [13]. GATK software was used to call the variants, as documented in GATK Best Practices [14]. Quality control was performed using VCFtools (version 4.2) [15] with a MAF of 0.05, missing rate of 0.1, Hardy-Weinberg equilibrium of 10^−6^, and SNPs with a mean depth greater than or equal to 6. SNPs located on sex chromosomes with no position information were then filtered from this dataset. Finally, the missing genotypes were imputed by Beagle software (version 5.1) [16], and this sequence data were used as a reference for genotype imputation.

### Genotype imputation

A combined reference population (including Yorkshire and Landrace breeds) was used to improve the accuracy of genotype imputation from GBS to sequence data. The imputation from GBS to WGS was conducted using Beagle software (version 5.1) [16] with the default parameters, except for setting the effective population size to 100. The sites with accuracy (allelic R^2^ value) of less than 0.90 and MAF of less than 0.01 were excluded from the imputed dataset. These quality control criteria generated 4,252,858 WGS variants for the 300 Landrace pigs used for GWAS.

### Genome-wide association studies

The imputed WGS was used to conduct a GWAS for NM. To begin, a normal distribution transformation $\sqrt{x+1}$ was applied to the phenotype data to make the results more reliable for association analysis, where *x* is the phenotype value. Then, we performed GWAS for NM using a linear mixed model in the GEMMA software [17], with a MAF of 0.05 and using the p-values from the likelihood ratio test. The model is as follows:


y=Wα+Xβ+Zu+e

where **y** is the NM value of the individuals; **α** is a vector of fixed effects including the year and month of sows farrowing; **β** is the SNP effects; **u** is the random effects and follows $\text{u}\sim \text{N}(0,\;\text{G}\sigma_{u}^{2})$, where G is the genomic relationship matrix; **W**, **X**, and **Z** are incidence matrices for **α**, **β**, and **u**; **e** is the residual effect.

The Manhattan and Q-Q plots were generated using R software. The significant threshold value was determined using the Bonferroni correction method. SNPs with p-values <1.18×10^−8^ were further determined as important loci linked to candidate genes that affect NM. The genes flanking the significant SNPs for each locus were considered candidate genes.

### Tissue samples

Ovarian, heart, liver, spleen, lung, kidney, leaf fat, subcutaneous fat, and lumbar muscle tissues were collected from multiparous Landrace sow from New Hope Group, Co., Ltd (Sichuan, China). Liquid nitrogen was used to transport tissue samples, which were stored at −80°C until needed.

### Quantitative real-time polymerase chain reaction

Total RNA was extracted from tissues and cells using the TRIzol reagent (Thermo Fisher Scientific, Shanghai, China) and was reverse-transcribed into cDNA by PrimeScript RT reagent Kit with gDNA Eraser (TaKaRa, Dalian, China). Quantitative real-time polymerase chain reaction (qPCR) analyses were conducted using CFX96 system (Bio-Rad, Hercules, CA, USA). The relative expression levels were calculated using the 2^−ΔΔ^*^Ct^* method, by normalizing to glyceraldehyde-3-phosphate dehydrogenase [18]. All primer sequences used are listed in Supplementary Table S3.

### Fluorescence *in situ* hybridization assay

Paraffin-embedded slices of porcine ovarian tissue were sectioned at 4 μm. Fluorescence *in situ* hybridization (FISH) assay was performed according to the paraffin-double fluorescence probe-FISH protocol (Servicebio, Wuhan, China). Following the FISH instructions, the ovarian slices were hybridized with a *CDK8* mRNA FISH probe and counterstained with 4′,6-diamidino-2-phenylin-dole. A fluorescence microscope (NIKON ECLIPSE CI, Tokyo, Japan) was used to take the photos.

### DNA pull-down assay

The DNA pull-down assay was performed using the BersinBio Bes5004 DNA pull-down Kit (BersinBio, Guangzhou, China) according to manufacturer’s instructions. The biotin-labeled DNA probe was designed according to *CDK8* promoter region. After washing with elution buffer, the retrieved protein was analyzed using liquid chromatography-mass spectrometry (LC/MS) on a Q Exactive Mass Spectrometer (Thermo, Shanghai, China). The mass-spectrometric data were analyzed using MaxQuant software (version 1.5.6.0) [19].

### Culture of porcine ovarian granulosa cells and transfection

Porcine ovarian granulosa cells of mature Bama Xiang pig were purchased from iCell Bioscience Inc. (Shanghai, China). These cells were cultured in Dulbecco’s modified Eagle medium (DMEM; PriMed-iCell-02; iCell Bioscience Inc., Shanghai, China) at 37°C in an atmosphere containing 5% CO_2_. Constructs overexpressing *YBX1* (p-*YBX1*) were obtained by cloning the cDNA of *YBX1* into a pCDNA3.1^+^ plasmid. Short hairpin RNA (shRNA) molecules against *YBX1* (sh*YBX1*) along with the negative control (NC) were designed and synthesized by Shanghai GenePharma Co., Ltd. (Shanghai, China) Ovarian granulosa cells were seeded into a 6-well plate. They were then transfected with p-*YBX1*, sh*YBX1*, and shNC using TransEasy (FOREGENE, Chengdu, China) according to the manufacturer’s instructions. The plasmid transfection efficiency was evaluated using an OlympusIX73 microscope. The primer sequences used are listed in Supplementary Table S4.

### Western blotting

The nuclear and cytoplasmic proteins were extracted using the Nuclear and Cytoplasmic Protein Extraction Kit (Beyotime, Shanghai, China) according to the manufacturer’s instructions. The concentration of proteins was evaluated using an enzyme-labeled instrument (Bio-Rad, USA), separated on a 12% sodium dodecyl sulfate-polyacrylamide gel electrophoresis, and transferred to polyvinylidene fluoride (PVDF) membrane. PVDF membrane was blocked with 5% non-fat milk in Tris-buffered saline with Tween (TBST) buffer for 90 min and incubated with primary antibodies (1:1,000) for 2 h at 37°C. Subsequently, PVDF membrane was washed five times for 10 min by TBST, incubated with a secondary antibody (1:2,000) for 2 h at 37°C, and washed five times for 5 min by TBST. The protein signal was detected with the Pierce ECL western blotting substrate (Thermo Fisher Scientific, China), and β-actin was used as the internal control.

### Statistical analysis

Each experiment was performed in triplicate, and the quantitative results were represented as the mean±standard deviation. Statistical analysis was performed using GraphPad Prism 5 (GraphPad Software Inc., San Diego, CA, USA) and SPSS 22.0 (IBM, Armonk, NY, USA) software. The differences were evaluated with the one-tailed t-test. Statistical significance was set as * p<0.05 and ** p<0.01.

## RESULTS

### Calculation of imputation accuracy

**Figure 1:**
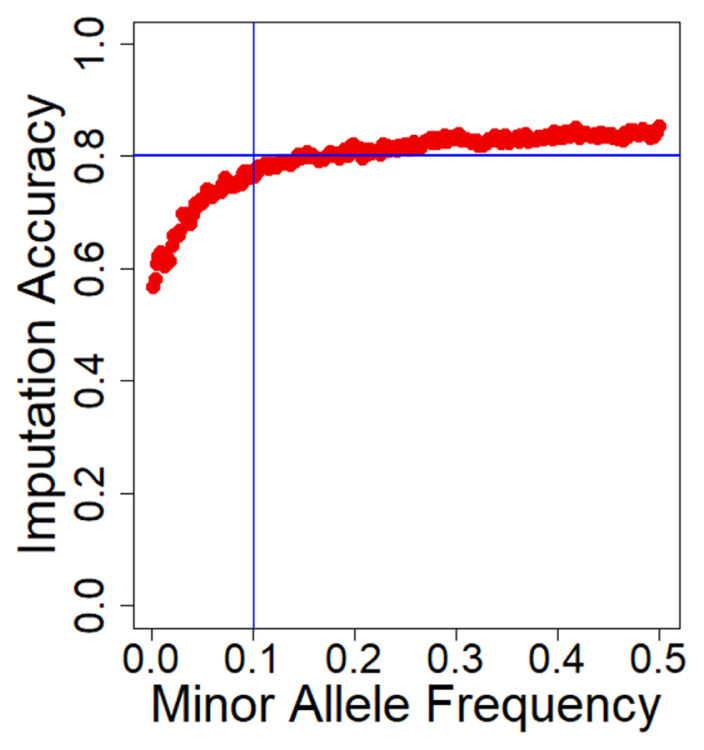
Average imputation accuracy against minor allele frequency (MAF). The x-axis is the MAF range from 0 to 0.5, and the y-axis is average imputation accuracy denoted by the Beagle R^2^. Single nucleotide polymorphisms (SNPs) were divided into bins of SNPs with common MAF. MAF, minor allele frequency; SNPs, single nucleotide polymorphisms.

We used the Beagle software (version 5.1) to impute 300 GBS data using 60 reference WGS data resulting in 14 million variants. To evaluate the imputation accuracy for the imputed data, we excluded sites with MAF less than 10E-6 and then computed the mean allelic R^2^ value across the range of MAF (Figure 1). Across all variants, the imputed WGS had a high mean allelic R^2^ of 0.73, which is sufficient for an association study. The allelic R^2^ was sensitive to MAF, which increased as MAF increased in the imputation. For the SNPs with a MAF less than 0.10, allelic R^2^ decreased significantly, and it kept a plateau at approximately 0.80 for the 0.1 to 0.5 MAF range. Furthermore, the imputation accuracy was also explored for each chromosome (Supplementary Table S5). The mean accuracy per chromosome was kept at a plateau for the 0.71 to 0.76 allelic R^2^ range. In general, there were no significant differences between chromosomes. After filtering the sites with MAF<0.01 and R^2^<0.90, 4,252,858 SNPs with a high mean R^2^ = 0.96 were retained for genetic analysis.

### GWAS reveals loci causing mummified embryo in pigs

**Figure 2:**
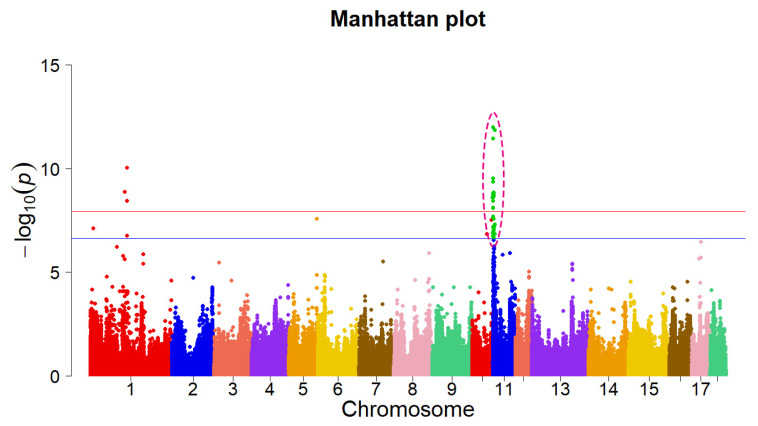
Genome-wide association studies (GWAS) results for the number of mummified (NM) in Landrace pigs. Each dot on this figure corresponds to an SNP within the dataset, while the horizontal red and blue lines denote the genome-wide significance (1.18E-08) and suggestive significance threshold (2.35E-07), respectively. GWAS, genome-wide association studies; NM, the number of mummified; SNP, single nucleotide polymorphisms.

To identify genomic variants associated with prenatal death in pigs, we performed a GWAS for the NM piglets at birth using the imputed WGS. A normal distribution transformation $\sqrt{x+1}$ was applied to the phenotypic data to make the results more reliable in association analysis. Based on the strict Bonferroni correction, a suggestive threshold of −log_10_ (p-values) >6.63 identified 3,603 SNPs distributed on SSC1, 5, 10, and 11 that were associated with NM in pigs, while a significant genome threshold of −log_10_ (p-values) >7.93 identified two chromosomal regions (SSC1: 133.00 to 141.90 Mbp and SSC11: 0.10 to 7.11 Mbp) with 2,793 variants (Figure 2; Supplementary Table S6). An attractive GWAS peak was typically detected on SSC11: 0.10 to 7.11 Mbp containing 2,762 consecutive SNPs. We next confirmed that this significant region was mapped close to a previously reported QTL for NM [20]. This indicated that this region might have important loci causing prenatal death in pigs. For further study, we focused on these significant loci located in the region of SSC11: 0.10 to 7.11 Mbp. A total of 44 genes were identified within this region, out of which *CDK8* was the nearest to the significant SNP. It was found to regulate embryonic development in previous literature [7,8].

### *CDK8* gene is associated with the porcine ovarian tissues and ovarian granulosa cells

**Figure 3:**
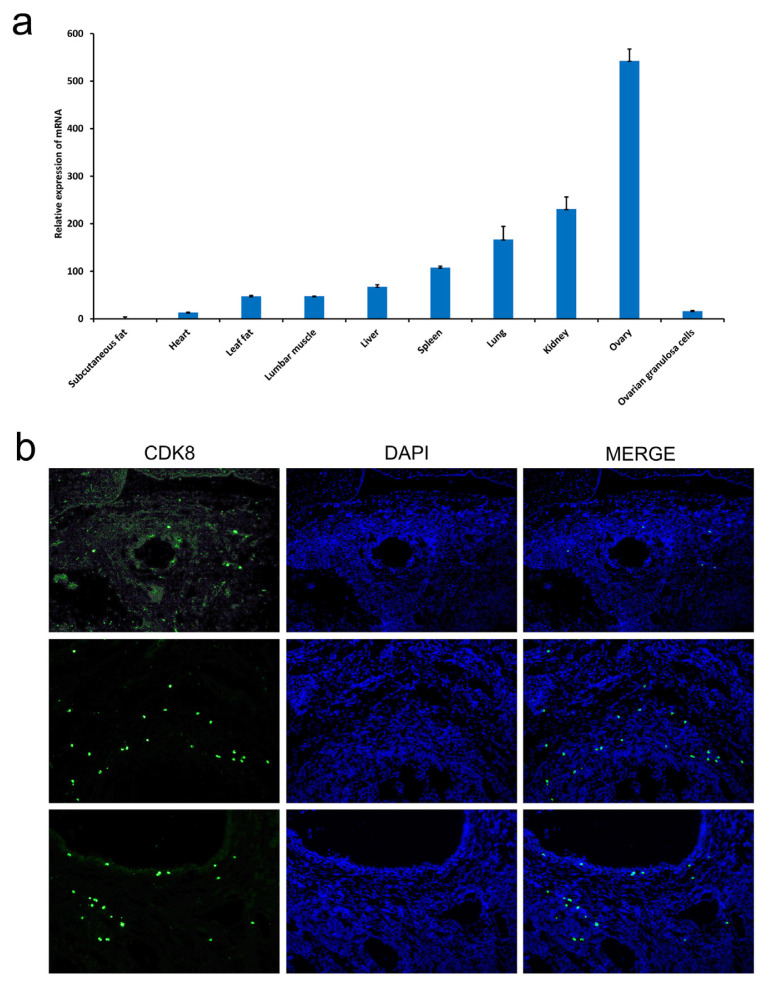
Identification and characterization of *CDK8* gene. (a) Tissue expression profile of *CDK8* gene. Each group contained at least 3 biologic replicate samples and results are shown as mean±standard deviation. (b) Localization of *CDK8* gene in ovarian tissue (40×, 200×). *CDK8*, cyclin dependent kinase 8.

We focused our study on the *CDK8* gene, which significantly affects NM and is essential for embryonic development. To identify the *CDK8* association with the porcine ovarian tissues, we measured its expression levels in different tissues of Landrace sow pigs. According to the quantitative real-time PCR (qRT-PCR), *CDK8* was expressed at the highest levels in porcine ovarian tissues compared to other tissues such as fat, heart, muscle, and so on (Figure 3a). The qRT-PCR and FISH analysis revealed that *CDK8* was expressed in the functional cells of the ovarian tissues, which are the granulosa cells (Figure 3). These findings indicated that *CDK8* gene might contribute to sow reproductive function.

### *YBX1* is a key transcription factor in *CDK8* gene transcription

**Figure 4:**
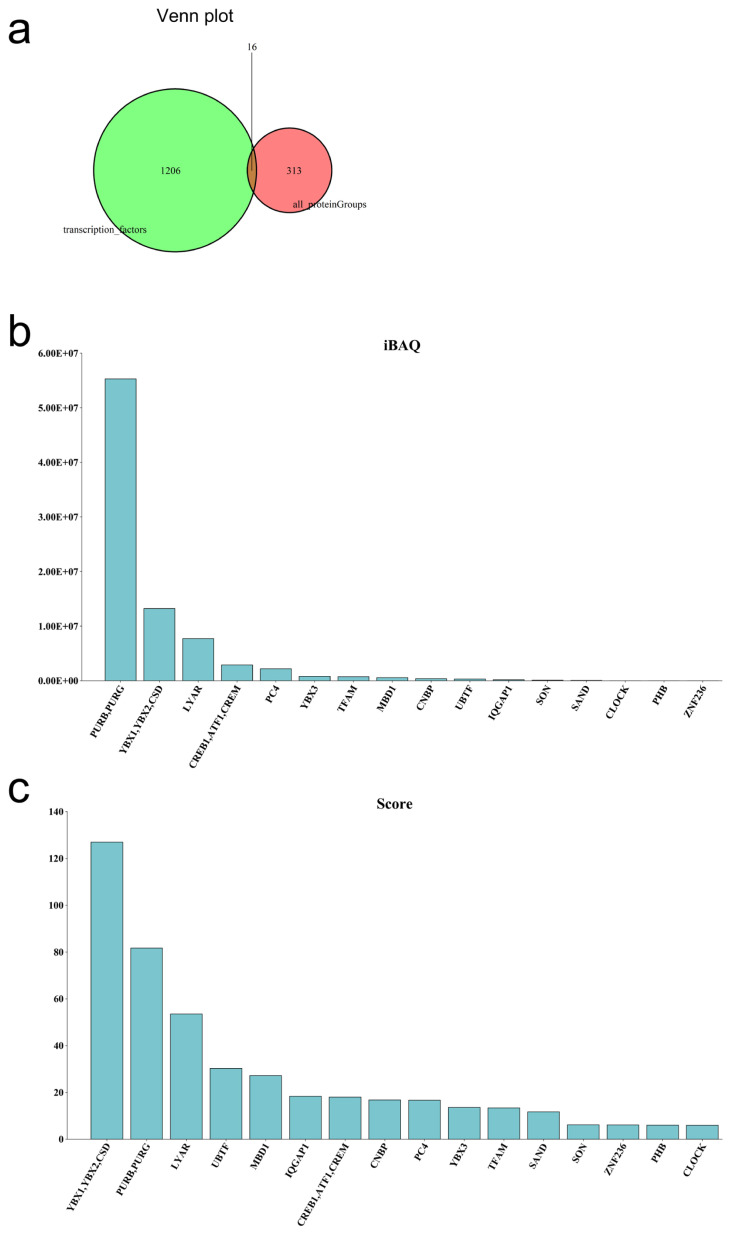
Identifying informative transcription factors of *CDK8* gene. (a) Wayne diagram of the identified transcription factors. (b) Histogram of transcription factor iBAQ value. (c) Histogram of transcription factor score. *CDK8*, cyclin dependent kinase 8; iBAQ, intensity based absolute quantification.

Gene transcription depends on the regulation of transcription factors in the biological process. A DNA pull-down assay using porcine ovarian tissues was used to confirm if transcription factors regulate CDK8. A total of 1,141 peptides and 329 proteins (FDR<0.01) were identified from ovarian tissues based on LC/MS analysis. We found 16 transcription factors (Figure 4a) when compared to the AnimalTFDB 3.0 ( http://bioinfo.life.hust.edu.cn/AnimalTFDB/). Intensity based absolute quantification (iBAQ) quantification methods showed that purine rich element binding protein B (*PURB*), purine rich element binding protein G (*PURG*), *YBX1*, *YBX2*, cold shock proteins (*CSD*), and Ly1 antibody reactive (*LYAR*) had high iBAQ and Score values (Figures 4b and 4c, Supplementary Table S7).

**Figure 5:**
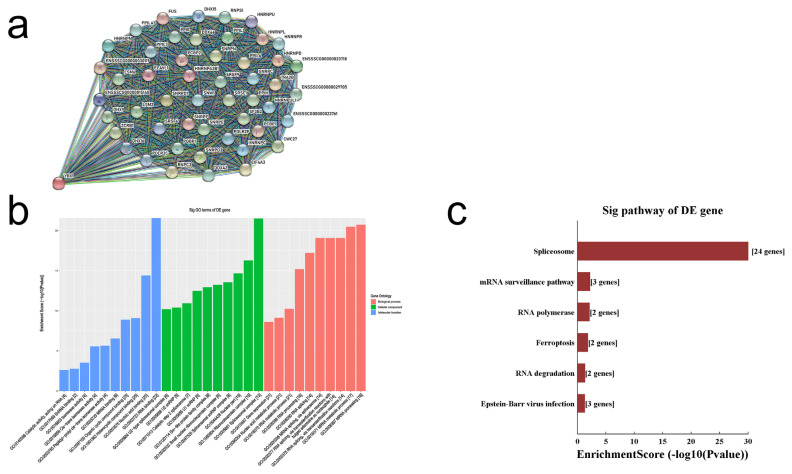
Exploring the biological functions for *YBX1* gene. (a) The interaction map of 51 interacting genes, (b) GO analysis for 51 interacting genes, (c) Pathway analysis for 51 interacting genes. *YBX1*, Y-box binding protein 1; GO, gene ontology.

A previous study reported that *YBX1* plays a pivotal role in embryonic development and results in prenatal fetal death in mice [12]. We drew the gene interaction network diagram for *YBX1*, the significant threshold was set at medium confidence 0.4, and the total number of interacting genes was 50 (Figure 5a). These interacting genes were mostly involved in the biological processes of RNA, according to gene ontology (GO) and Kyoto encyclopedia of genes and genomes analyses (Figures 5b and 5c). The results revealed that *YBX1* is an important transcription regulator in biological processes and may affect *CDK8* expression. Thus, we focused our analysis on *YBX1* for further investigation in porcine ovarian granulosa cells.

### *CDK8* gene promotes the proliferation of porcine ovarian granulosa cells through TGF-**β**/SMAD signaling pathway

**Figure 6:**
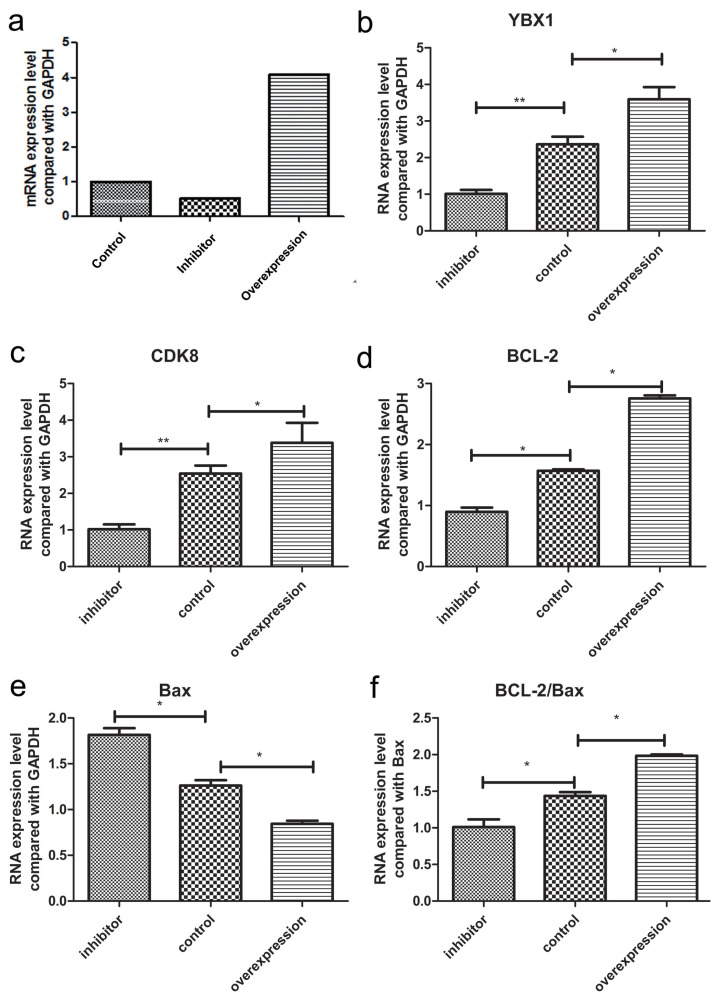
The mRNA expression level after *YBX1* treated with overexpression and inhibitor in porcine ovarian granulosa cells. (a) Calculation of porcine ovarian granulocyte *YBX1* overexpression and inhibition efficiency. (b–f) The mRNA expression level of *YBX1*, *CDK8*, *BCL-2*, *Bax*, and *BCL-2/Bax* gene in overexpression and inhibitor groups. Each group contained at least 3 biologic replicate samples and results are shown as mean±standard deviation. *YBX1*, Y-box binding protein 1; *CDK8*, cyclin dependent kinase 8; *BCL-2*, BCL2 apoptosis regulator; *Bax*, BCL2 associated X, apoptosis regulator; *BCL-2/Bax*, BCL2 apoptosis regulator/BCL2 associated X, apoptosis regulator. * Indicates p<0.05, ** indicates p<0.01.

To further identify the function of *CDK8* in porcine ovarian granulosa cells, we assessed whether the overexpression and silencing of *YBX1* affected the expression of *CDK8* and other genes involved in the transforming growth factor beta/small mother against decapentaplegic (TGF-β/SMAD) signaling pathway in these cells. The silencing efficiency of *YBX1* was 49.18% (Figure 6a) using pGPU6/GFP/Neo-YBX1-474. By transfecting pCDNA3.1-*YBX1* and empty plasmid, the relative expression level of overexpressing *YBX1* was 4.07 (Figure 6a), which suggested efficient gene transfer in porcine ovarian granulosa cells.

**Figure 7:**
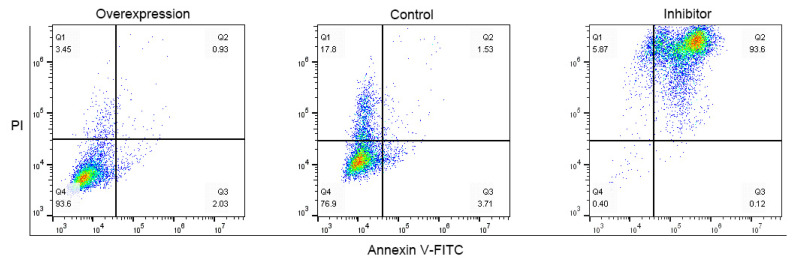
Effect of *YBX1* on apoptosis porcine ovarian granulosa cells after interfered and or overexpression. Representative of three experimental replicates with porcine ovarian granulosa cells. Flow cytometry was formed using fluorescer conjugated Annexin V-FITC and PI double staining. *YBX1*, Y-box binding protein 1; V-FITC, fluorescently labeled Annexin V; PI, propidium iodide.

*CDK8*, ovarian granulosa cell proliferation, and anti-apoptotic gene (BCL2 apoptosis regulator [*BCL-2*]) were significantly up-regulated (p<0.05) by overexpressing *YBX1* in ovarian granulosa cells transfected with p-YBX1. In contrast, pro-apoptotic gene (BCL2 associated X, apoptosis regulator [*BAX*]) was significantly down-regulated (p<0.05) (Figures 6b to 6f). *CDK8* (p<0.01) proliferation of ovarian granulosa cells (p<0.05), and *BCL-2* (p<0.01) were all significantly down-regulated, when *YBX1* expression was inhibited, whereas *BAX* was significantly up-regulated (p< 0.05) (Figures 6b to 6f). Meanwhile, flow cytometry results displayed that overexpressing *YBX1* promoted the proliferation of ovarian granulosa cells, whereas inhibitor *YBX1* inhibited their proliferation (Figure 7).

**Figure 8:**
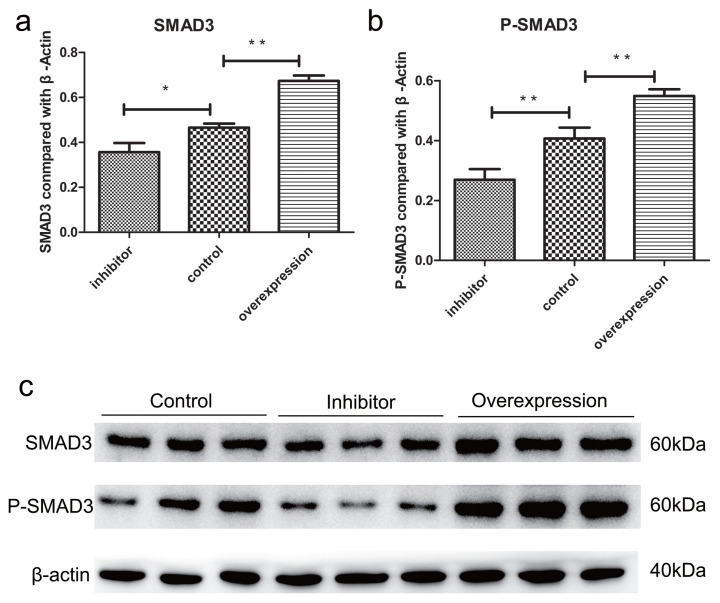
Effect of YBX1 on the protein expression levels of SMAD3, P-SMAD3, and β-actin after interfered and overexpression treatment. (a–b) The gray degree values analysis of SMAD3 and P-SMAD3 in Western blot. (c) Western blot analysis showed the level of SMAD3, P-SMAD3, and β-actin protein abundance. Each group contained at least 3 biologic replicate samples and results are shown as mean±standard deviation. YBX1, Y-box binding protein 1; SMAD3, small mother against decapentaplegic 3; P-SMAD3, phospho small mother against decapentaplegic 3. * Indicate p<0.05, ** indicate p<0.01.

CDK8 phosphorylated the T179 site of SMAD3. To reveal molecular mechanisms involved in the proliferation of ovarian granulosa cells underlying *YBX1* mediated *CDK8*, we then analyzed the expression of SMAD3 and P-SMAD3 in the TGF-β/SMAD signaling pathway. According to western blotting results, P-SMAD3 was significantly increased by overexpressing *YBX1* and reduced by interfering with its expression in ovarian granulosa cells, while SMAD3 was not significantly affected by overexpressing or interfering with *YBX1* in ovarian granulosa cells (Figure 8). These results indicated that *YBX1*-mediated *CDK8* promotes porcine ovarian granulosa cell proliferation through TGF-β/SMAD signaling pathway.

## DISCUSSION

This study demonstrated the advantages of using imputed WGS to conduct genetic analysis in pig complex traits. The imputed WGS contained most of the genetic variants, thus greatly improving the identification of causal variants in GWAS. Our experiments revealed that the number of early fetal deaths varies greatly. Association analysis linked this genetic variation to *CDK8* gene, which is involved in transcription regulation and has previously been linked to early embryo development [8]. Overexpression and inhibition of *YBX1* had dramatic effects on the proliferation of porcine ovarian granulosa cells, as well as the expression level of *CDK8* and *SMAD3* in the TGF-β/SMAD signaling pathway, indicating that *YBX1*-mediated *CDK8* may play a pivotal role in regulating porcine ovarian granulosa cells via TGF-β/SMAD signaling pathway, and further influence embryo development in pigs.

IUGR is a failure of normal embryo growth and development during pregnancy, resulting in severe fetal death and deficiency. In pigs, mummified fetal death occurs before farrowing and may be caused by IUGR. A previous study showed that the number of mummified fetuses positively correlated with the number of IUGR in pigs [21], indicating a common genetic background. However, the molecular mechanisms underlying IUGR in pigs are unknown. Exploring the genetic mechanism of mummified fetuses may contribute to understanding IUGR better. In addition, IUGR has a significant impact on human health. Pigs are closely associated with humans in terms of anatomy, genomics, and physiology, so they are often used as an optimal biomedical model for human diseases [22]. In humans, research is mainly focused on nutritional intervention to decrease the rate of IUGR. Our study on genetic variants of NM would provide new insights into the genetic mechanisms underlying IUGR in humans.

### GWAS reveals important NM-associated loci in pigs

Previous studies have identified 159 QTLs for offspring mortality (death of the offspring during embryonic/fetal development) in pigs. A QTL mapping study using a low-density chip (PorcineSNP60 BeadChip) identified two QTLs on chromosome 11 [20]. However, no follow-up study investigated the genes underlying these QTLs. A comprehensive whole-genome association study is expected to uncover the genetic variants and genome regions that explain the variation in the targeted trait and improve the genetic selection in pig offspring mortality trait. Compared to previous studies, our imputed WGS displayed a much higher number of variants (over 4 million SNPs), out of which 2,793 significant SNPs had a distinct GWAS peak in the 0.10 to 7.11 Mbp region of chromosome 11. In pigs, numerous significant associations overlapped with three known reproductive QTLs for NM [20], teat number [23], and uterine horn length [24]. However, none of the genes identified in our study overlapped with the previous studies. The overlapped associations were considered evidence for replication in reproductive traits, and non-overlaps implicated that our study revealed some loci and candidate genes for pig offspring mortality trait. Furthermore, based on the follow-up molecular experiments, we verified one gene that regulates the proliferation of porcine ovarian granulosa cells. These results broadened our knowledge of offspring mortality traits and confirmed the effectiveness of imputed WGS in GWAS as a beneficial genetic tool for complex phenotypes in pigs. Furthermore, fetal loss is also a common occurrence in humans. Considering the physiological and genetic similarities between pigs and human [22], improvement of imputed GWAS in pig offspring mortality would not only improve the field of pig genetic architecture, but also could provide a useful, informative indication for human fetal impairment and death.

### *YBX1* is a crucial transcription factor of *CDK8*

To further explore the biological role of *CDK8* in ovarian granulosa cells, we assessed if any transcription factor regulated its expression. We identified six important transcription factors (*PURB*, *PURG*, *YBX1*, *YBX2*, *CSD*, and *LYAR*) using a DNA pull-down assay and mass spectrometry analysis. The Pur family proteins, *PURB* and *PURG*, are crucial in regulating DNA replication, gene expression, and cell growth. The previous studies have revealed that *PURB* is widely expressed in most tissues and cells, whereas *PURG* is not [25]. *PURB* has been linked to developing cells, brain, and disease [26] and regulating milk synthesis [27]. However, the molecular functions of *PURG* remain unknown. *LYAR* was found to be highly expressed in mouse embryonic stem cells and immature spermatocytes, implicating its involvement in cell growth regulation [28]. The Y-box protein-encoding genes, *YBX1* and *YBX2*, are evolutionarily conserved in eukaryotes and are linked to DNA repair, transport, and translational regulation [29]. Previous work has shown that *YBX2* was highly expressed in post-meiotic male germ cells and played a pivotal role in male fertility and spermatid differentiation in mice [30]. *YBX1* expression is found throughout murine embryonic development [31], and the *YBX1* deficient embryos resulted in an embryonic lethality phenotype from the late embryonic to the early neonatal stage [31]; this provides an insight into its function in pig fetal death. Therefore, *YBX1* was identified as a critical transcription factor in exploring the function of *CDK8* in the pig ovarian.

### *CDK8* may play a role in embryo development through the TGF-β/SMAD signaling pathway

The *CDK8* has been shown to play an important role in transcriptional regulation [32]. It was abundantly expressed in pig ovarian tissues [33], consistent with our findings. We further identified that *CDK8* was also expressed in porcine ovarian granulosa cells using both FISH and qPCR assay. Therefore, we concluded that *CDK8* might be related to reproductive traits in pigs. The transforming growth factor (TGF) is one of the important key regulators of embryo development and tissue homeostasis. The TGF-β signaling pathway is one of the most crucial developmental signaling pathways involved in regulating cellular and molecular processes in development and disease, which is important for regulating apoptosis, follicular atresia, and thus affecting reproductive performance in porcine ovarian granulosa cells [34]. *CDK8* has previously been shown to drive SMAD transcriptional activation and turnover through the TGF-β pathway [35]. In addition, we verified that the T179 site of SMAD3 was phosphorylated by *CDK8*, which regulates TGF-β/SMAD signaling pathway and promotes the proliferation of ovarian granulosa cells in pigs. Similarly, phosphorylation of agonist-activated SMADs by *CDK8* resulted in SMAD-dependent transcription before triggering SMAD turnover [35]. The ovarian granulosa cells were important in regulating ovarian function and consequently affecting embryo production. Granulosa cells in mammals may protect oocytes and secrete steroid hormones to maintain a favorable environment for early ovarian follicular development, fertilization, implantation, and embryo development [36]. The quality of granulosa cells has been shown to affect embryo quality significantly and directly influence the pregnancy outcome [37]. In this study, *CDK8* was highly expressed in pig ovarian granulosa cells and promoted their proliferation through the TGF-β/SMAD pathway. In summary, based on the regulation of *YBX1*, *CDK8* gene interacts with the TGF-β/SMAD signaling pathway, a crucial pathway affecting the proliferation of ovarian granulosa cells. These results implied that this signaling pathway may have an important impact on embryo quality and results in prenatal death in pigs.

## CONCLUSION

To the best of our knowledge, this is the first study to use a GBS imputation panel based on WGS for the genetic architecture of NM in Landrace pigs, as well as to confirm the biological function of a key candidate gene (*CDK8*) in porcine ovarian granulosa cells. Our imputed GWAS firstly revealed numerous significant loci with a distinct GWAS peak and determined a promising candidate gene (*CDK8*) for NM in Landrace pigs. Then, we validated that the transcription factor *YBX1* mediates *CDK8*, affecting the proliferation of porcine ovarian granulosa cells through TGF-β/SMAD signaling pathway. These results showed that *YBX1*-mediated *CDK8* through TGF-β/SMAD signaling pathway, which might contribute to prenatal death in pigs. However, fine mapping in this region and functional studies are still needed to determine mechanisms by which the loci affected NM in pigs.

## Figures and Tables

**Table 1:** Summary statistics of the analyzed phenotypes in Landrace pigs

Phenotype	N	Mean±SD	MAX	MIN	CV
NM	300	0.27±0.92	10	0	3.41

N, the number of individuals; Mean, arithmetic mean; SD, standard deviation; MAX, maximum; MIN, minimum; CV, coefficient of variation; NM, the number of mummified piglets.

## SUPPLEMENTARY MATERIAL

The datasets supporting the results of this article are included within the article. The Supplemental Tables S1–7 were deposited in Figshare doi: https://doi.org/10.6084/m9.figshare.16778731

**Supplementary Table S1**. Summary and mapping statistics of 300 Landrace pigs using genotyping by sequencing

**Supplementary Table S2**. Summary and mapping statistics of 40 Yorkshire pigs and 20 Landrace pigs using whole-genome sequencing

**Supplementary Table S3**. The primer information of mRNA

**Supplementary Table S4**. shRNA sequence and primer information

**Supplementary Table S5**. Imputation accuracy and the number of SNPs calculated for each chromosome.

**Supplementary Table S6**. Genome-wide significant and suggestive SNPs for NM in Landrace pigs

**Supplementary Table S7**. Summary of transcription factor information
