# Supplementary material for: Whole-genome sequence association study identifies cyclin dependent kinase 8 as a key gene for the number of mummified piglets
Source: Anim Biosci. 2022 Sep 7;36(1):29–42. doi: 10.5713/ab.22.0115 (PMC9834657; doi:10.5713/ab.22.0115)
Supplement: Supplementary file 1 [file ab-22-0115-suppl1.pdf]

Supplementary Table 1. Summary and mapping statistics of 300 Landrace pigs using genotyping by sequencing

sample	Raw Base(bp)	Clean Base(bp)	Effective Rate(%)	Error Rate(%)	Q20(%)	Q30(%)	GC Content(%)	clean reads	mapped reads	mapping rate(%)	average depth(X)	coverage at least 1X(%)	coverage at least 4X(%)	Raw Base(bp)	Clean Base(bp)	Effective Rate(%)	Error Rate(%)	Q20(%)	Q30(%)	GC Content(%)
39481LF	784770624	784640160	99.98	0.02	97.33	94.09	43.2	5448890	5167424	94.83	11.66	4.89	2.09	784770624	784640160	99.98	0.02	97.33	94.09	43.2
14173LF	639086688	638981280	99.98	0.01	97.47	94.43	43.2	4437370	4209129	94.86	10.39	4.28	1.89	639086688	638981280	99.98	0.01	97.47	94.43	43.2
27317LF	900576000	900472608	99.99	0.01	97.54	94.55	40.73	6253282	5934523	94.9	15.23	4.86	1.87	900576000	900472608	99.99	0.01	97.54	94.55	40.73
38844LF	873567360	873461088	99.99	0.01	97.55	94.58	42.13	6065702	5754235	94.87	13.19	5.32	2.05	873567360	873461088	99.99	0.01	97.55	94.58	42.13
94415LF	763347168	763257024	99.99	0.01	97.52	94.49	42.25	5300396	5030650	94.91	12.1	5.11	1.91	763347168	763257024	99.99	0.01	97.52	94.49	42.25
30258LF	650702304	650615040	99.99	0.01	97.54	94.6	41.16	4518160	4287748	94.9	12	3.9	1.71	650702304	650615040	99.99	0.01	97.54	94.6	41.16
39454LF	919464768	919346400	99.99	0.01	97.62	94.77	41.76	6384350	6051654	94.79	13.65	5.7	2.06	919464768	919346400	99.99	0.01	97.62	94.77	41.76
39579LF	879432768	879329376	99.99	0.01	97.57	94.62	42.03	6106454	5788212	94.79	13.32	5.39	2.03	879432768	879329376	99.99	0.01	97.57	94.62	42.03
8577LF	819214848	819121824	99.99	0.02	97.33	94.1	41.69	5688346	5411718	95.14	11.9	6.36	1.95	819214848	819121824	99.99	0.02	97.33	94.1	41.69
24179LF	960519744	960393024	99.99	0.01	97.52	94.59	41.94	6669396	6337139	95.02	14.54	5.21	2.09	960519744	960393024	99.99	0.01	97.52	94.59	41.94
38507LF	1033847136	1033733952	99.99	0.01	97.47	94.43	41.5	7178708	6809013	94.85	14.7	6.36	2.13	1033847136	1033733952	99.99	0.01	97.47	94.43	41.5
82100LF	867119040	867017088	99.99	0.01	97.47	94.48	41.72	6020952	5721617	95.03	13.97	4.59	2	867119040	867017088	99.99	0.01	97.47	94.48	41.72
23780LF	874737792	874622880	99.99	0.01	97.55	94.62	41.88	6073770	5764986	94.92	13.6	5.03	2.02	874737792	874622880	99.99	0.01	97.55	94.62	41.88
39482LF	368931168	368893728	99.99	0.01	97.59	94.62	40.28	2561762	2432792	94.97	8.86	3.23	1.16	368931168	368893728	99.99	0.01	97.59	94.62	40.28
27386LF	584554464	584482464	99.99	0.01	97.45	94.35	40.46	4058906	3857866	95.05	10.29	5.25	1.53	584554464	584482464	99.99	0.01	97.45	94.35	40.46
18617LF	967085856	966975264	99.99	0.01	97.54	94.53	42.17	6715106	6364678	94.78	12.88	6.53	2.23	967085856	966975264	99.99	0.01	97.54	94.53	42.17
18623LF	960881472	960777504	99.99	0.01	97.42	94.31	41.59	6672066	6349312	95.16	12.5	7.54	2.13	960881472	960777504	99.99	0.01	97.42	94.31	41.59
31182LF	1050855264	1050722496	99.99	0.01	97.52	94.5	41.7	7296684	6937922	95.08	14.7	6.47	2.17	1050855264	1050722496	99.99	0.01	97.52	94.5	41.7
11745LF	1056637440	1056504960	99.99	0.01	97.38	94.36	40.91	7336840	6988769	95.26	14.14	7.14	2.18	1056637440	1056504960	99.99	0.01	97.38	94.36	40.91
23002LF	1092342240	1092201120	99.99	0.01	97.52	94.56	41.07	7584730	7209148	95.05	15.46	6.56	2.15	1092342240	1092201120	99.99	0.01	97.52	94.56	41.07
23852LF	983104416	982978848	99.99	0.01	97.45	94.43	41.02	6826242	6489455	95.07	13.89	6.59	2.09	983104416	982978848	99.99	0.01	97.45	94.43	41.02
38055LF	1144705536	1144547712	99.99	0.01	97.38	94.36	41.46	7948248	7552264	95.02	15.17	7.08	2.27	1144705536	1144547712	99.99	0.01	97.38	94.36	41.46
23438LF	1079827200	1079689536	99.99	0.01	97.53	94.59	41.56	7497844	7133532	95.14	15.11	6.35	2.2	1079827200	1079689536	99.99	0.01	97.53	94.59	41.56
11450LF	908301600	908207136	99.99	0.02	97.37	94.12	41.54	6306994	5991637	95	13.78	5.68	2.01	908301600	908207136	99.99	0.02	97.37	94.12	41.54
30314LF	866134080	866036160	99.99	0.01	97.5	94.45	42.33	6014140	5707343	94.9	12.98	5.29	2.06	866134080	866036160	99.99	0.01	97.5	94.45	42.33
11977LF	963784224	963665568	99.99	0.01	97.65	94.78	42.48	6692122	6345491	94.82	13.82	5.44	2.19	963784224	963665568	99.99	0.01	97.65	94.78	42.48
26709LF	906204096	906101856	99.99	0.02	97.5	94.41	42.03	6292374	5974689	94.95	12.76	6.11	2.12	906204096	906101856	99.99	0.02	97.5	94.41	42.03
11707LF	990762912	990634464	99.99	0.01	97.48	94.53	42.09	6879406	6529128	94.91	14.51	5.31	2.17	990762912	990634464	99.99	0.01	97.48	94.53	42.09
96915LF	970972704	970840224	99.99	0.01	97.49	94.54	41.94	6741946	6399555	94.92	14.38	5.21	2.15	970972704	970840224	99.99	0.01	97.49	94.54	41.94
27266LF	941897664	941798880	99.99	0.01	97.56	94.62	42.41	6540270	6208139	94.92	14.38	4.96	2.1	941897664	941798880	99.99	0.01	97.56	94.62	42.41
8060LF	1075756320	1075620096	99.99	0.01	97.56	94.65	42.03	7469584	7092850	94.96	15.41	5.54	2.23	1075756320	1075620096	99.99	0.01	97.56	94.65	42.03
30147LF	971304480	971194752	99.99	0.01	97.46	94.35	42.05	6744408	6403993	94.95	13.86	5.96	2.14	971304480	971194752	99.99	0.01	97.46	94.35	42.05
30885LF	604573920	604470240	99.98	0.01	97.6	94.35	42.45	4197710	3978343	94.77	7.87	5.78	2.01	604573920	604470240	99.98	0.01	97.6	94.35	42.45
37789LF	1306409472	1306254240	99.99	0.01	97.6	94.69	41.35	9071210	8604438	94.85	18.82	5.66	2.27	1306409472	1306254240	99.99	0.01	97.6	94.69	41.35
36718LF	992777472	992661120	99.99	0.01	97.46	94.38	41.82	6893480	6536201	94.82	14.95	5.36	2.1	992777472	992661120	99.99	0.01	97.46	94.38	41.82
18118LF	877387104	877301856	99.99	0.02	97.05	93.34	41.97	6092374	5779353	94.86	13.67	5.18	2	877387104	877301856	99.99	0.02	97.05	93.34	41.97
39368LF	944925984	944819424	99.99	0.01	97.59	94.66	42.26	6561246	6223085	94.85	13.49	5.86	2.14	944925984	944819424	99.99	0.01	97.59	94.66	42.26
93633LF	783535680	783442656	99.99	0.01	97.53	94.53	42.42	5440574	5158964	94.82	11.51	5.61	2.01	783535680	783442656	99.99	0.01	97.53	94.53	42.42
18115LF	844853760	844726752	99.98	0.01	97.26	94.06	40.53	5866158	5563815	94.85	12.34	5.73	2.05	844853760	844726752	99.98	0.01	97.26	94.06	40.53
39577LF	1130127552	1129938624	99.98	0.01	97.25	94.05	42	7846796	7444051	94.87	13.69	6.33	2.59	1130127552	1129938624	99.98	0.01	97.25	94.05	42
24883LF	987804576	987638976	99.98	0.01	97.25	94.02	41.7	6858604	6509763	94.91	11.81	7.27	2.42	987804576	987638976	99.98	0.01	97.25	94.02	41.7
37953LF	1072769472	1072591776	99.98	0.01	97.33	94.22	41.17	7448554	7063552	94.83	13.98	5.86	2.42	1072769472	1072591776	99.98	0.01	97.33	94.22	41.17
20080LF	1167024672	1166842368	99.98	0.01	97.39	94.34	41.41	8103072	7693496	94.95	13.52	7.39	2.6	1167024672	1166842368	99.98	0.01	97.39	94.34	41.41
37166LF	1027794240	1027634976	99.98	0.01	97.31	94.17	42.1	7136354	6765193	94.8	12.55	6.35	2.51	1027794240	1027634976	99.98	0.01	97.31	94.17	42.1
39597LF	984148416	984002400	99.99	0.02	97.13	93.72	41.45	6833350	6493134	95.02	11.92	7.46	2.35	984148416	984002400	99.99	0.02	97.13	93.72	41.45
20486LF	1080600480	1080413856	99.98	0.01	97.22	94.09	41.63	7502874	7126358	94.98	13.34	6.35	2.52	1080600480	1080413856	99.98	0.01	97.22	94.09	41.63
38851LF	1054534464	1054367136	99.98	0.01	97.17	93.91	41.41	7321994	6956902	95.01	13.02	6.74	2.45	1054534464	1054367136	99.98	0.01	97.17	93.91	41.41
94621LF	1091586528	1091420352	99.98	0.01	97.25	94.08	41.77	7579308	7196169	94.94	13.8	5.88	2.51	1091586528	1091420352	99.98	0.01	97.25	94.08	41.77
94002LF	1154064384	1153888128	99.98	0.01	97.29	94.17	41.77	8013112	7601059	94.86	13.72	6.51	2.63	1154064384	1153888128	99.98	0.01	97.29	94.17	41.77
39385LF	1076429088	1076265792	99.98	0.01	97.39	94.28	40.69	7474068	7094046	94.92	14.51	6.11	2.31	1076429088	1076265792	99.98	0.01	97.39	94.28	40.69
18628LF	940129056	940004064	99.99	0.01	97.17	93.86	40.66	6527806	6196299	94.92	12.2	6.83	2.22	940129056	940004064	99.99	0.01	97.17	93.86	40.66
25973LF	894760128	894632256	99.99	0.01	97.26	94.03	41.98	6212724	5899972	94.97	11.38	6.16	2.35	894760128	894632256	99.99	0.01	97.26	94.03	41.98
38056LF	931463712	931326048	99.99	0.01	97.15	93.81	41.32	6467542	6153456	95.14	11.08	7.59	2.32	931463712	931326048	99.99	0.01	97.15	93.81	41.32
38279LF	1002385728	1002227616	99.98	0.01	97.22	94.01	41.53	6959914	6610837	94.98	12.27	6.9	2.41	1002385728	1002227616	99.98	0.01	97.22	94.01	41.53
20487LF	1019480832	1019312064	99.98	0.01	97.12	93.92	40.62	7078556	6743332	95.26	11.16	8.51	2.44	1019480832	1019312064	99.98	0.01	97.12	93.92	40.62
18627LF	1180911744	1180723680	99.98	0.01	97.27	94.12	40.98	8199470	7791448	95.02	13.87	7.58	2.53	1180911744	1180723680	99.98	0.01	97.27	94.12	40.98
25714LF	1039640256	1039478112	99.98	0.01	97.12	93.88	40.75	7218598	6866045	95.12	13.3	6.44	2.38	1039640256	1039478112	99.98	0.01	97.12	93.88	40.75
36719LF	1112782176	1112595264	99.98	0.01	97.07	93.81	41.23	7726356	7344818	95.06	13.25	7.26	2.51	1112782176	1112595264	99.98	0.01	97.07	93.81	41.23
18487LF	1161398592	1161213120	99.98	0.01	97.23	94.09	41.23	8063980	7634283	94.67	13.89	7.09	2.53	1161398592	1161213120	99.98	0.01	97.23	94.09	41.23
18862LF	1005619680	1005483456	99.99	0.02	97.15	93.75	41.41	6982524	6633355	95	12.33	6.96	2.41	1005619680	1005483456	99.99	0.02	97.15	93.75	41.41
8576LF	964186272	964054656	99.99	0.01	97.2	93.92	42.28	6694824	6351313	94.87	12.32	5.84	2.43	964186272	964054656	99.99	0.01	97.2	93.92	42.28
38723LF	898272576	898142400	99.99	0.01	97.33	94.22	42.23	6237100	5917499	94.88	11.55	5.67	2.4	898272576	898142400	99.99	0.01	97.33	94.22	42.23
7856LF	851215392	851094720	99.99	0.02	97.27	93.98	42.05	5910380	5611837	94.95	11.42	5.71	2.25	851215392	851094720	99.99	0.02	97.27	93.98	42.05
20475LF	913426272	913288608	99.98	0.01	97.2	94.05	41.84	6342282	6012725	94.8	11.94	5.62	2.37	913426272	913288608	99.98	0.01	97.2	94.05	41.84
19349LF	1004679936	1004499936	99.98	0.01	97.18	94	41.7	6975694	6624336	94.96	12.85	5.83	2.45	1004679936	1004499936	99.98	0.01	97.18	94	41.7
94414LF	951718176	951569280	99.98	0.01	97.23	94.06	42.09	6608120	6272119	94.92	12.91	5.15	2.36	951718176	951569280	99.98	0.01	97.23	94.06	42.09
18443LF	1095678720	1095508512	99.98	0.01	97.23	94.06	41.66	7607698	7224660	94.97	14.01	5.86	2.49	1095678720	1095508512	99.98	0.01	97.23	94.06	41.66
89682LF	639207648	639190080	100	0.02	96.94	93.03	43.25	4438820	4204568	94.72	9.59	4.71	1.98	639207648	639190080	100	0.02	96.94	93.03	43.25
99575LF	349996896	349992864	100	0.02	96.93	92.75	44.14	2430506	2298093	94.55	7.44	3.5	1.22	349996896	349992864	100	0.02	96.93	92.75	44.14
85697LF	789496704	789370848	99.98	0.02	97.5	94.14	42.88	5481742	5200928	94.88	10.13	6.05	2.24	789496704	789370848	99.98	0.02	97.5	94.14	42.88
87421LF	447665760	447657696	100	0.02	97	93.08	42.84	3108734	2942697	94.66	7.9	4.1	1.55	447665760	447657696	100	0.02	97	93.08	42.84
91692LF	442605312	442596672	100	0.02	96.86	92.74	43.26	3073588	2910975	94.71	7.84	4.03	1.55	442605312	442596672	100	0.02	96.86	92.74	43.26
1087LF	399337056	399335040	100	0.02	96.18	91.04	43.39	2773160	2624303	94.63	7.73	3.6	1.43	399337056	399335040	100	0.02	96.18	91.04	43.39
60955LF	557166240	557158176	100	0.02	96.95	93.03	43.3	3869154	3666533	94.76	8.85	4.63	1.79	557166240	557158176	100	0.02	96.95	93.03	43.3
55594LF	536981760	536969664	100	0.02	96.94	92.97	43.37	3728956	3532192	94.72	8.65	4.57	1.75	536981760	536969664	100	0.02	96.94	92.97	43.37
3577LF	740041632	739937952	99.99	0.02	97.23	93.52	43.09	5138458	4880678	94.98	11.54	4.51	2.02	740041632	739937952	99.99	0.02	97.23	93.52	43.09
66098LF	743955264	743896224	99.99	0.02	97.2	93.59	44.12	5165946	4900900	94.87	11.96	3.84	2.03	743955264	743896224	99.99	0.02	97.2	93.59	44.12
3608LF	524407104	524372544	99.99	0.02	97.12	93.41	44.28	3641476	3451180	94.77	9.58	3.44	1.7	524407104	524372544	99.99	0.02	97.12	93.41	44.28
65777LF	697825728	697762944	99.99	0.01	97.25	93.72	43.62	4845576	4593296	94.79	11.61	3.67	1.96	697825728	697762944	99.99	0.01	97.25	93.72	43.62
5776LF	518691744	518645376	99.99	0.01	97.27	93.78	43.83	3601704	3415001	94.82	9.41	3.53	1.7	518691744	518645376	99.99	0.01	97.27	93.78	43.83
1337LF	673622496	673514208	99.98	0.02	97.44	94.06	42.15	4677182	4435868	94.84	11.24	4.09	1.89	673622496	673514208	99.98	0.02	97.44	94.06	42.15
84565LF	547780320	547747776	99.99	0.02	96.88	92.81	44.31	3803804	3606512	94.81	9.82	3.55	1.74	547780320	547747776	99.99	0.02	96.88	92.81	44.31
94230LF	323259264	323226720	99.99	0.01	97.15	93.63	44.34	2244630	2131425	94.96	7.16	3.12	1.2	323259264	323226720	99.99	0.01	97.15	93.63	44.34
114LF	493281792	493245216	99.99	0.02	97.03	93.31	44.33	3425314	3245527	94.75	9.17	3.49	1.63	493281792	493245216	99.99	0.02	97.03	93.31	44.33

89316LF	538490592	538447392	99.99	0.01	97.15	93.56	44.33	3739218	3546700	94.85	9.71	3.5	1.73	538490592	538447392	99.99	0.01	97.15	93.56	44.33
84516LF	650038752	649981152	99.99	0.01	97.16	93.58	44.35	4513758	4279203	94.8	10.85	3.77	1.91	650038752	649981152	99.99	0.01	97.16	93.58	44.35
3572LF	601982784	601947648	99.99	0.02	97.23	93.56	43.2	4180192	3960971	94.76	10.73	3.73	1.76	601982784	601947648	99.99	0.02	97.23	93.56	43.2
85705LF	560807424	560778624	99.99	0.02	97.09	93.31	43.34	3894296	3689019	94.73	10.04	3.7	1.73	560807424	560778624	99.99	0.02	97.09	93.31	43.34
3574LF	605984544	605951424	99.99	0.02	97.15	93.34	44.45	4207996	3987205	94.75	10.41	3.72	1.83	605984544	605951424	99.99	0.02	97.15	93.34	44.45
110LF	466117632	466092576	99.99	0.02	96.99	93.09	44.3	3236754	3069554	94.83	8.84	3.43	1.58	466117632	466092576	99.99	0.02	96.99	93.09	44.3
73175LF	550224288	550185120	99.99	0.02	97.16	93.47	44.53	3820730	3621576	94.79	9.99	3.48	1.73	550224288	550185120	99.99	0.02	97.16	93.47	44.53
96540LF	683541504	683486208	99.99	0.01	97.17	93.62	44.42	4746432	4498720	94.78	11.38	3.69	1.95	683541504	683486208	99.99	0.01	97.17	93.62	44.42
51511LF	371099232	371069856	99.99	0.01	97.35	93.84	44.91	2576874	2437913	94.61	8.45	3.04	1.26	371099232	371069856	99.99	0.01	97.35	93.84	44.91
84847LF	732063456	732004704	99.99	0.01	97.14	93.5	43.98	5083366	4816134	94.74	11.74	3.85	2.03	732063456	732004704	99.99	0.01	97.14	93.5	43.98
87325LF	513741024	513684864	99.99	0.01	97.06	93.47	44.42	3567256	3382389	94.82	9.47	3.44	1.68	513741024	513684864	99.99	0.01	97.06	93.47	44.42
7928LF	607666752	607620096	99.99	0.01	97.16	93.61	44.5	4219584	4000309	94.8	10.43	3.68	1.84	607666752	607620096	99.99	0.01	97.16	93.61	44.5
86138LF	646653312	646620192	99.99	0.02	96.84	92.66	44.02	4490418	4250827	94.66	10.9	3.69	1.9	646653312	646620192	99.99	0.02	96.84	92.66	44.02
87362LF	371141856	371124576	100	0.02	96.38	91.51	44.33	2577254	2438980	94.63	8.01	3.12	1.32	371141856	371124576	100	0.02	96.38	91.51	44.33
56237LF	645639552	645584832	99.99	0.01	97.12	93.47	44.25	4483228	4251809	94.84	10.91	3.64	1.91	645639552	645584832	99.99	0.01	97.12	93.47	44.25
99419LF	497479392	497441376	99.99	0.02	97.11	93.45	44.29	3454454	3276188	94.84	9.15	3.58	1.64	497479392	497441376	99.99	0.02	97.11	93.45	44.29
942LF	1144566144	1144513152	100	0.03	96.41	91.46	42.81	7948008	7546437	94.95	14.26	5.61	2.61	1144566144	1144513152	100	0.03	96.41	91.46	42.81
90171LF	1233823392	1233762912	100	0.03	95.98	90.43	42.72	8567798	8121266	94.79	15.46	5.44	2.63	1233823392	1233762912	100	0.03	95.98	90.43	42.72
91676LF	1583931744	1583851392	99.99	0.03	96.44	91.69	42.67	10998968	10439054	94.91	18.02	6.08	2.94	1583931744	1583851392	99.99	0.03	96.44	91.69	42.67
90288LF	1307240928	1307179008	100	0.03	96.25	91.2	42.5	9077632	8611483	94.86	16.03	5.64	2.7	1307240928	1307179008	100	0.03	96.25	91.2	42.5
78126LF	1466810208	1466736192	99.99	0.03	96.39	91.5	42.59	10185668	9663313	94.87	17.13	5.97	2.85	1466810208	1466736192	99.99	0.03	96.39	91.5	42.59
87423LF	1460975904	1460899872	99.99	0.03	96.37	91.44	42.45	10145138	9626930	94.89	17.43	5.71	2.81	1460975904	1460899872	99.99	0.03	96.37	91.44	42.45
88510LF	1350402912	1350339840	100	0.03	96.47	91.53	41.07	9377360	8899024	94.9	17.01	6.56	2.55	1350402912	1350339840	100	0.03	96.47	91.53	41.07
92141LF	950274432	950227488	100	0.03	96.21	91.02	41.41	6598802	6254257	94.78	13.38	5	2.28	950274432	950227488	100	0.03	96.21	91.02	41.41
3624LF	1206774720	1206730368	100	0.03	96.25	91.02	42.7	8380072	7950737	94.88	14.97	5.59	2.64	1206774720	1206730368	100	0.03	96.25	91.02	42.7
20323LF	963668448	963625536	100	0.03	96.14	90.81	42.56	6691844	6351587	94.92	12.53	5.83	2.4	963668448	963625536	100	0.03	96.14	90.81	42.56
19951LF	1154575872	1154519712	100	0.03	96.32	91.28	42.67	8017498	7607326	94.88	14.7	5.32	2.58	1154575872	1154519712	100	0.03	96.32	91.28	42.67
38310LF	1243121472	1243050048	99.99	0.03	96.37	91.54	42.58	8632292	8193776	94.92	15.59	5.34	2.65	1243121472	1243050048	99.99	0.03	96.37	91.54	42.58
39599LF	1185102432	1185038208	99.99	0.03	96.39	91.5	42.33	8229432	7796875	94.74	15.22	5.2	2.58	1185102432	1185038208	99.99	0.03	96.39	91.5	42.33
18595LF	1269319680	1269260352	100	0.03	96.31	91.34	42.25	8814308	8362315	94.87	16.04	5.34	2.63	1269319680	1269260352	100	0.03	96.31	91.34	42.25
37739LF	1475030592	1474949664	99.99	0.03	96.28	91.41	42.58	10242706	9712870	94.83	17.67	5.77	2.79	1475030592	1474949664	99.99	0.03	96.28	91.41	42.58
38044LF	1386548064	1386463680	99.99	0.03	96.42	91.58	42.69	9628220	9131913	94.85	16.67	5.71	2.76	1386548064	1386463680	99.99	0.03	96.42	91.58	42.69
39578LF	1169218656	1169167680	100	0.03	95.98	90.39	42.5	8119220	7698167	94.81	14.98	5.32	2.57	1169218656	1169167680	100	0.03	95.98	90.39	42.5
39459LF	1261036800	1260986112	100	0.03	96.22	91	42.88	8756848	8298767	94.77	15.59	5.6	2.66	1261036800	1260986112	100	0.03	96.22	91	42.88
19953LF	1427009760	1426934592	99.99	0.03	96.49	91.69	42.55	9909268	9395667	94.82	16.55	6.17	2.84	1427009760	1426934592	99.99	0.03	96.49	91.69	42.55
37508LF	781513056	781459776	99.99	0.02	96.4	91.98	41.95	5426804	5157829	95.04	12.26	4.22	2.04	781513056	781459776	99.99	0.02	96.4	91.98	41.95
14309LF	798382656	798320160	99.99	0.02	96.41	92.1	43.19	5543890	5278306	95.21	11.76	4.31	2.19	798382656	798320160	99.99	0.02	96.41	92.1	43.19
38327LF	744364512	744313536	99.99	0.02	96.31	91.83	43.3	5168844	4913083	95.05	11.17	4.24	2.12	744364512	744313536	99.99	0.02	96.31	91.83	43.3
36982LF	754611264	754552224	99.99	0.02	96.48	92.23	43.03	5239946	4975576	94.95	11.48	4.17	2.11	754611264	754552224	99.99	0.02	96.48	92.23	43.03
37070LF	686783232	686718720	99.99	0.02	96.55	92.41	42.96	4768880	4537472	95.15	10.67	4.12	2.03	686783232	686718720	99.99	0.02	96.55	92.41	42.96
18863LF	600555744	600511968	99.99	0.02	96.44	92.1	43.5	4170222	3967496	95.14	9.76	4.01	1.89	600555744	600511968	99.99	0.02	96.44	92.1	43.5
20074LF	708950592	708912576	99.99	0.02	96	91.09	43.08	4923004	4682273	95.11	10.86	4.06	2.09	708950592	708912576	99.99	0.02	96	91.09	43.08
37139LF	927696672	927615744	99.99	0.02	96.5	92.39	43.56	6441776	6125024	95.08	12.63	4.82	2.36	927696672	927615744	99.99	0.02	96.5	92.39	43.56
37948LF	929121696	929049984	99.99	0.02	96.33	91.91	43.36	6451736	6131133	95.03	12.79	4.74	2.34	929121696	929049984	99.99	0.02	96.33	91.91	43.36
14211LF	1025184960	1025100576	99.99	0.02	96.44	92.19	43.48	7118754	6767085	95.06	13.41	5.11	2.47	1025184960	1025100576	99.99	0.02	96.44	92.19	43.48
38419LF	891094176	891024192	99.99	0.02	96.47	92.22	43.49	6187668	5877063	94.98	11.93	5.05	2.35	891094176	891024192	99.99	0.02	96.47	92.22	43.49
39598LF	936844992	936794016	99.99	0.02	96.57	92.2	42.11	6505514	6173496	94.9	13.47	4.61	2.25	936844992	936794016	99.99	0.02	96.57	92.2	42.11
13503LF	583522272	583489152	99.99	0.02	96.42	91.94	42.61	4052008	3847819	94.96	9.82	3.94	1.82	583522272	583489152	99.99	0.02	96.42	91.94	42.61
94171LF	632474784	632443968	100	0.02	96.38	91.78	43.59	4391972	4169492	94.93	10.06	4.16	1.94	632474784	632443968	100	0.02	96.38	91.78	43.59
89041LF	609701184	609664608	99.99	0.02	96.26	91.59	43.45	4233782	4020795	94.97	9.89	3.99	1.91	609701184	609664608	99.99	0.02	96.26	91.59	43.45
24292LF	583160832	583117632	99.99	0.02	96.49	92.14	43.78	4049428	3848485	95.04	9.53	4.24	1.83	583160832	583117632	99.99	0.02	96.49	92.14	43.78
94215LF	626201856	626141088	99.99	0.02	96.44	92.25	43.65	4348202	4134560	95.09	9.93	4.2	1.93	626201856	626141088	99.99	0.02	96.44	92.25	43.65
89020LF	663302592	663245856	99.99	0.02	96.53	92.31	43.58	4605874	4375913	95.01	10.42	4.18	1.98	663302592	663245856	99.99	0.02	96.53	92.31	43.58
3571LF	725135040	725068224	99.99	0.02	96.42	92.08	43.36	5035196	4783587	95	10.91	4.38	2.09	725135040	725068224	99.99	0.02	96.42	92.08	43.36
862LF	780359904	780281568	99.99	0.02	96.35	92.11	43.62	5418622	5151665	95.07	11.56	4.35	2.16	780359904	780281568	99.99	0.02	96.35	92.11	43.62
84899LF	949122432	949070016	99.99	0.02	96.19	91.31	43.17	6590764	6261051	95	12.7	5.02	2.39	949122432	949070016	99.99	0.02	96.19	91.31	43.17
46246LF	899917344	899861760	99.99	0.02	96.44	91.94	43.66	6249040	5932541	94.94	12.12	5.09	2.34	899917344	899861760	99.99	0.02	96.44	91.94	43.66
83125LF	935633664	935554752	99.99	0.02	96.66	92.53	43.59	6496908	6173096	95.02	12.18	5.32	2.42	935633664	935554752	99.99	0.02	96.66	92.53	43.59
2636LF	652134528	652103424	100	0.02	96.38	91.79	43.13	4528496	4302963	95.02	10.11	4.28	1.99	652134528	652103424	100	0.02	96.38	91.79	43.13
2036LF	648425376	648352800	99.99	0.02	96.46	92.29	43.56	4502450	4271616	94.87	10.12	4.25	1.97	648425376	648352800	99.99	0.02	96.46	92.29	43.56
66023LF	564041664	563982336	99.99	0.02	96.47	92.29	43.59	3916544	3723458	95.07	9.26	4.13	1.81	564041664	563982336	99.99	0.02	96.47	92.29	43.59
69331LF	526693536	526657536	99.99	0.02	96.59	92.36	43.76	3657344	3475668	95.03	9.02	4	1.72	526693536	526657536	99.99	0.02	96.59	92.36	43.76
96913LF	534880512	534835008	99.99	0.02	96.56	92.38	43.59	3714132	3529117	95.02	9.05	3.95	1.76	534880512	534835008	99.99	0.02	96.56	92.38	43.59
87364LF	585694656	585662688	99.99	0.02	96.29	91.62	43.58	4067102	3860080	94.91	9.61	3.99	1.86	585694656	585662688	99.99	0.02	96.29	91.62	43.58
99421LF	685154592	685124640	100	0.02	96.11	91.16	43.28	4757810	4520267	95.01	10.53	4.28	2.03	685154592	685124640	100	0.02	96.11	91.16	43.28
3664LF	794083968	794029536	99.99	0.02	96.57	92.33	43.36	5514094	5235624	94.95	11.2	4.86	2.2	794083968	794029536	99.99	0.02	96.57	92.33	43.36
84796LF	856288224	856222272	99.99	0.02	96.42	91.91	43.42	5945988	5651064	95.04	11.72	4.9	2.31	856288224	856222272	99.99	0.02	96.42	91.91	43.42
89828LF	731691936	731669760	100	0.03	95.44	89.61	43.41	5081040	4822023	94.9	10.83	4.47	2.11	731691936	731669760	100	0.03	95.44	89.61	43.41
97596LF	838333152	838266048	99.99	0.02	96.49	92.22	43.52	5821292	5529977	95	11.47	5.04	2.28	838333152	838266048	99.99	0.02	96.49	92.22	43.52
90271LF	830301120	830236320	99.99	0.02	96.47	92.16	43.55	5765530	5480628	95.06	10.95	5.43	2.3	830301120	830236320	99.99	0.02	96.47	92.16	43.55
65435LF	604158336	604022112	99.98	0.03	96	91.23	41.38	4194598	3973605	94.73	10.24	4.14	1.79	604158336	604022112	99.98	0.03	96	91.23	41.38
53255LF	611242272	611110944	99.98	0.03	95.91	91.16	42.59	4243826	4022403	94.78	9.36	4.25	1.99	611242272	611110944	99.98	0.03	95.91	91.16	42.59
53918LF	543399840	543278304	99.98	0.03	95.83	90.98	42.55	3772766	3577172	94.82	8.72	3.92	1.89	543399840	543278304	99.98	0.03	95.83	90.98	42.55
99653LF	583159104	583012224	99.97	0.02	96	91.34	42.25	4048696	3834739	94.72	9.34	4.05	1.9	583159104	583012224	99.97	0.02	96	91.34	42.25
89644LF	538736832	538610112	99.98	0.02	96.08	91.55	42.36	3740348	3544110	94.75	8.73	4	1.85	538736832	538610112	99.98	0.02	96.08	91.55	42.36
84875LF	428311008	428216256	99.98	0.03	95.99	91.25	42.86	2973724	2818717	94.79	7.45	3.94	1.57	428311008	428216256	99.98	0.03	95.99	91.25	42.86
77727LF	550232928	550122624	99.98	0.03	95.62	90.38	42.72	3820296	3620425	94.77	8.67	4.17	1.88	550232928	550122624	99.98	0.03	95.62	90.38	42.72
3583LF	766644192	766464480	99.98	0.02	96.03	91.52	42.91	5322670	5046813	94.82	10.42	4.93	2.27	766644192	766464480	99.98	0.02	96.03	91.52	42.91
4050LF	783934272	783764640	99.98	0.03	95.86	91.17	42.87	5442810	5157552	94.76	10.73	4.86	2.27	783934272	783764640	99.98	0.03	95.86	91.17	42.87
93728LF	832291200	832089888	99.98	0.03	95.95	91.3	42.84	5778402	5475246	94.75	10.99	5.09	2.36	832291200	832089888	99.98	0.03	95.95	91.3	42.84
116LF	759729312	759552192	99.98	0.02	95.96	91.26	42.74	5274668	4999264	94.78	10.31	4.98	2.26	759729312	759552192	99.98	0.02	95.96	91.26	42.74
53967LF	858434688	858255552	99.98	0.03	96.07	91.31	41.41	5960108	5643958	94.7	12.28	4.84	2.21	858434688	858255552	99.98	0.03	96.07	91.31	41.41
53740LF	610750368	610631136	99.98	0.03	95.94	91.2	41.66	4240494	4013158	94.64	9.72	4.14	1.92	610750368	610631136	99.98	0.03	95.94	91.2	41.66
93584LF	524552544	524464992	99.98	0.03	95.81	90.79	42.83	3642118	3450068	94.73	8.49	4.02	1.83	524552544	524464992	99.98	0.03	95.81	90.79	42.83
2874LF	493374816	493278912	99.98	0.03	95.81	90.87	42.65	3425548	3245181	94.73	8.21	3.87	1.77	493374816	493278912	99.98	0.03	95.81	90.87	42.65
77295LF	531056160	530933184	99.98	0.03	95.86	91.04	42.86	3687036	3496927	94.84	8.64	3.96	1.84	531056160	530933184	99.98	0.03	95.86	91.04	42.86
85487LF	554452416	554301216	99.97	0.02	95.91	91.3	42.74	3849314	3649499	94.81	8.79	4.14	1.88	554452416	554301216	99.97	0.02	95.91	91.3	42.74
31113LF	548979840	548839008	99.97	0.03	95.97	91.3	42.61	3811382	3612982	94.79	8.82	4.01	1.87	548979840	548839008	99.97	0.03	95.97	91.3	42.61

82235LF	683303040	683142624	99.98	0.03	95.88	91.13	42.42	4744046	4492592	94.7	10.18	4.28	2.1	683303040	683142624	99.98	0.03	95.88	91.13	42.42
87995LF	816653664	816431328	99.97	0.02	95.87	91.23	42.98	5669662	5375679	94.81	10.94	4.99	2.33	816653664	816431328	99.97	0.02	95.87	91.23	42.98
348LF	805906368	805693824	99.97	0.02	96.02	91.43	42.98	5595096	5303056	94.78	10.4	5.32	2.36	805906368	805693824	99.97	0.02	96.02	91.43	42.98
5534LF	751529088	751377312	99.98	0.03	95.73	90.55	42.35	5217898	4943963	94.75	10.29	4.88	2.25	751529088	751377312	99.98	0.03	95.73	90.55	42.35
91331LF	783789984	783616896	99.98	0.03	95.89	91.03	42.75	5441784	5155867	94.75	10.52	4.99	2.3	783789984	783616896	99.98	0.03	95.89	91.03	42.75
38240LF	823383360	823182624	99.98	0.03	96.13	91.56	42.8	5716546	5410577	94.65	10.69	5.29	2.36	823383360	823182624	99.98	0.03	96.13	91.56	42.8
14148LF	673701408	673581312	99.98	0.03	95.96	91.06	42.35	4677648	4433331	94.78	9.99	4.33	2.1	673701408	673581312	99.98	0.03	95.96	91.06	42.35
10329LF	636599520	636433344	99.97	0.02	95.96	91.42	42.77	4419676	4188642	94.77	9.51	4.4	2.04	636599520	636433344	99.97	0.02	95.96	91.42	42.77
1080LF	615658464	615504672	99.98	0.02	95.97	91.38	42.84	4274338	4054648	94.86	9.4	4.27	2	615658464	615504672	99.98	0.02	95.97	91.38	42.84
79683LF	608804064	608663520	99.98	0.03	96.04	91.41	42.77	4226830	4005663	94.77	9.34	4.21	1.99	608804064	608663520	99.98	0.03	96.04	91.41	42.77
84550LF	592964352	592813152	99.97	0.03	96.09	91.57	42.68	4116758	3899695	94.73	9.16	4.2	1.96	592964352	592813152	99.97	0.03	96.09	91.57	42.68
73021LF	523389312	523276704	99.98	0.03	95.76	90.67	42.76	3633866	3443174	94.75	8.48	4.02	1.83	523389312	523276704	99.98	0.03	95.76	90.67	42.76
85696LF	669646080	669526848	99.98	0.03	95.71	90.54	42.16	4649492	4404508	94.73	10.04	4.26	2.08	669646080	669526848	99.98	0.03	95.71	90.54	42.16
86325LF	868118688	867916800	99.98	0.03	96.07	91.42	42.35	6027200	5709750	94.73	11.48	5.23	2.36	868118688	867916800	99.98	0.03	96.07	91.42	42.35
3573LF	819642816	819472896	99.98	0.03	95.94	91.1	42.47	5690784	5390881	94.73	10.73	5.08	2.37	819642816	819472896	99.98	0.03	95.94	91.1	42.47
60265LF	701539776	701448192	99.99	0.03	95.07	89.04	42.56	4871168	4607617	94.59	10.06	4.53	2.16	701539776	701448192	99.99	0.03	95.07	89.04	42.56
115LF	908909856	908699904	99.98	0.03	95.94	91.28	42.6	6310416	5976805	94.71	11.49	5.45	2.46	908909856	908699904	99.98	0.03	95.94	91.28	42.6
83314LF	915764832	915541344	99.98	0.03	95.9	91.13	42.81	6357926	6025926	94.78	11.66	5.53	2.44	915764832	915541344	99.98	0.03	95.9	91.13	42.81
3713LF	821984832	821966976	100	0.03	96.05	91.42	41.77	5708104	5407952	94.74	12.63	4.62	2.05	821984832	821966976	100	0.03	96.05	91.42	41.77
82134LF	994225248	994204512	100	0.03	96.06	91.49	42.84	6904198	6549166	94.86	12.84	5.81	2.41	994225248	994204512	100	0.03	96.06	91.49	42.84
1911LF	808394400	808379424	100	0.03	95.92	91.19	43.28	5613746	5319965	94.77	11.51	4.89	2.18	808394400	808379424	100	0.03	95.92	91.19	43.28
57253LF	831100320	831080160	100	0.02	96.13	91.62	42.72	5771390	5468837	94.76	11.95	4.79	2.19	831100320	831080160	100	0.02	96.13	91.62	42.72
3519LF	794066400	794048256	100	0.02	96.16	91.77	42.79	5514224	5227641	94.8	11.51	4.74	2.16	794066400	794048256	100	0.02	96.16	91.77	42.79
3566LF	669829536	669815136	100	0.02	96.07	91.45	43.33	4651494	4411932	94.85	9.96	4.8	2	669829536	669815136	100	0.02	96.07	91.45	43.33
82034LF	694479456	694465056	100	0.03	95.5	90.26	43.37	4822674	4569605	94.75	10.38	4.75	2.02	694479456	694465056	100	0.03	95.5	90.26	43.37
44736LF	901933344	901910880	100	0.02	96.25	91.97	43.1	6263270	5941328	94.86	11.08	5.9	2.46	901933344	901910880	100	0.02	96.25	91.97	43.1
89510LF	915875712	915854976	100	0.02	96.11	91.67	43.26	6360104	6029884	94.81	11.97	5.43	2.38	915875712	915854976	100	0.02	96.11	91.67	43.26
3570LF	997543872	997522272	100	0.02	96.16	91.72	43.02	6927238	6565282	94.77	11.86	6.21	2.56	997543872	997522272	100	0.02	96.16	91.72	43.02
92121LF	828045792	828027936	100	0.02	96.06	91.5	43.14	5750194	5447137	94.73	10.67	5.62	2.32	828045792	828027936	100	0.02	96.06	91.5	43.14
85821LF	768713184	768700512	100	0.03	96.13	91.47	42.16	5338198	5055128	94.7	11.35	5	2.06	768713184	768700512	100	0.03	96.13	91.47	42.16
90167LF	675831456	675816768	100	0.03	95.81	90.87	42.81	4693172	4451249	94.85	10.44	4.38	2	675831456	675816768	100	0.03	95.81	90.87	42.81
73801LF	677632032	677617920	100	0.02	96.17	91.86	42.95	4705680	4461790	94.82	10.18	4.53	2.03	677632032	677617920	100	0.02	96.17	91.86	42.95
92854LF	678478464	678462624	100	0.02	96.16	91.8	42.94	4711546	4470640	94.89	10.14	4.61	2.04	678478464	678462624	100	0.02	96.16	91.8	42.94
73566LF	743930208	743915232	100	0.03	96.09	91.57	43.38	5166078	4900208	94.85	10.95	4.67	2.1	743930208	743915232	100	0.03	96.09	91.57	43.38
89681LF	810684288	810660960	100	0.02	96.1	91.7	43.11	5629590	5337843	94.82	11.63	4.68	2.2	810684288	810660960	100	0.02	96.1	91.7	43.11
78042LF	727822368	727807104	100	0.03	95.66	90.56	43.33	5054216	4791263	94.8	10.87	4.51	2.08	727822368	727807104	100	0.03	95.66	90.56	43.33
90165LF	734685984	734675616	100	0.03	95.5	90.27	42.57	5101914	4837090	94.81	11.25	4.14	2.08	734685984	734675616	100	0.03	95.5	90.27	42.57
57208LF	980216352	980191296	100	0.03	96.06	91.51	42.66	6806884	6450391	94.76	13	5.21	2.4	980216352	980191296	100	0.03	96.06	91.51	42.66
54431LF	929088288	929070720	100	0.03	95.81	90.96	43.05	6451880	6115816	94.79	12.09	5.35	2.4	929088288	929070720	100	0.03	95.81	90.96	43.05
93623LF	835388064	835378272	100	0.03	94.75	88.51	43.2	5801238	5493162	94.69	11.57	4.98	2.25	835388064	835378272	100	0.03	94.75	88.51	43.2
67990LF	962959680	962940096	100	0.02	96.05	91.53	42.9	6687084	6337027	94.77	12.15	5.78	2.45	962959680	962940096	100	0.02	96.05	91.53	42.9
50869LF	836344224	836325504	100	0.02	96.07	91.5	42.84	5807816	5507936	94.84	10.76	5.96	2.29	836344224	836325504	100	0.02	96.07	91.5	42.84
73568LF	1043585856	1043457408	99.99	0.01	97.96	95.5	42.17	7246232	6882610	94.98	12.72	6.19	2.54	1043585856	1043457408	99.99	0.01	97.96	95.5	42.17
8076LF	694539072	694427328	99.98	0.04	94.27	87.65	43.02	4822412	4557429	94.51	10.06	5.06	2.03	694539072	694427328	99.98	0.04	94.27	87.65	43.02
5568LF	810856512	810726048	99.98	0.04	94.05	87.16	43.25	5630042	5320704	94.51	11.64	4.76	2.17	810856512	810726048	99.98	0.04	94.05	87.16	43.25
73020LF	823874400	823742496	99.98	0.04	94.23	87.62	42.3	5720434	5411602	94.6	12.41	4.4	2.12	823874400	823742496	99.98	0.04	94.23	87.62	42.3
96544LF	780348096	780220224	99.98	0.04	94.36	87.93	42.37	5418196	5125689	94.6	11.74	4.47	2.09	780348096	780220224	99.98	0.04	94.36	87.93	42.37
90466LF	831099744	830959776	99.98	0.04	94.2	87.44	43.33	5770554	5458006	94.58	11.43	5.16	2.23	831099744	830959776	99.98	0.04	94.2	87.44	43.33
84876LF	658940544	658847808	99.99	0.04	93.48	85.95	42.02	4575332	4328153	94.6	9.67	5.23	1.96	658940544	658847808	99.99	0.04	93.48	85.95	42.02
55432LF	784446336	784309248	99.98	0.04	94.38	88.05	42.8	5446592	5155993	94.66	11.51	4.48	2.15	784446336	784309248	99.98	0.04	94.38	88.05	42.8
81848LF	798451776	798321600	99.98	0.04	94.12	87.44	42.86	5543900	5243668	94.58	11.32	5.2	2.14	798451776	798321600	99.98	0.04	94.12	87.44	42.86
7804LF	756593568	756469152	99.98	0.04	94.27	87.7	43	5253258	4971071	94.63	11.18	4.54	2.11	756593568	756469152	99.98	0.04	94.27	87.7	43
84898LF	802603584	802475136	99.98	0.04	94.06	87.31	43.18	5572744	5266437	94.5	11.52	4.7	2.17	802603584	802475136	99.98	0.04	94.06	87.31	43.18
60047LF	699114816	699015168	99.99	0.04	94	87	41.48	4854272	4589813	94.55	11.44	4.38	1.87	699114816	699015168	99.99	0.04	94	87	41.48
66415LF	515824416	515745216	99.98	0.04	93.82	86.74	42.52	3581564	3378488	94.33	9.08	3.99	1.64	515824416	515745216	99.98	0.04	93.82	86.74	42.52
82219LF	620479872	620392320	99.99	0.04	93.68	86.32	43.52	4308280	4069882	94.47	9.72	4.36	1.91	620479872	620392320	99.99	0.04	93.68	86.32	43.52
95470LF	649473984	649385856	99.99	0.04	93.7	86.43	43.23	4509624	4265155	94.58	10.18	4.22	1.96	649473984	649385856	99.99	0.04	93.7	86.43	43.23
83345LF	691270848	691148160	99.98	0.04	94.16	87.4	43.16	4799640	4540816	94.61	10.31	4.78	2.01	691270848	691148160	99.98	0.04	94.16	87.4	43.16
85485LF	792809280	792678816	99.98	0.04	94.4	88.09	43.06	5504714	5204392	94.54	11.5	4.62	2.16	792809280	792678816	99.98	0.04	94.4	88.09	43.06
92228LF	777414240	777289248	99.98	0.04	94.32	87.81	42.78	5397842	5105384	94.58	11.62	4.44	2.11	777414240	777289248	99.98	0.04	94.32	87.81	42.78
87001LF	751104000	750971232	99.98	0.04	94.19	87.54	42.46	5215078	4928055	94.5	11.43	4.35	2.06	751104000	750971232	99.98	0.04	94.19	87.54	42.46
57717LF	827963136	827815680	99.98	0.04	94.43	88.12	43.28	5748720	5429518	94.45	11.61	4.96	2.2	827963136	827815680	99.98	0.04	94.43	88.12	43.28
89081LF	902049984	901907136	99.98	0.04	94.4	87.97	43.23	6263244	5921821	94.55	12.49	5	2.28	902049984	901907136	99.98	0.04	94.4	87.97	43.23
85463LF	738114624	738011520	99.99	0.04	93.57	86.02	42.76	5125080	4845078	94.54	10.98	4.81	2.05	738114624	738011520	99.99	0.04	93.57	86.02	42.76
40817LF	781997472	781886880	99.99	0.04	93.9	86.83	43.34	5429770	5129485	94.47	11.45	4.6	2.13	781997472	781886880	99.99	0.04	93.9	86.83	43.34
99892LF	680146848	680044608	99.98	0.04	94.37	87.83	43.21	4722532	4460917	94.46	9.97	5.05	1.99	680146848	680044608	99.98	0.04	94.37	87.83	43.21
2290LF	669928032	669833856	99.99	0.04	93.81	86.5	43.15	4651624	4393425	94.45	10.1	4.63	1.98	669928032	669833856	99.99	0.04	93.81	86.5	43.15
99431LF	685244736	685118592	99.98	0.04	94.59	88.35	43.57	4757768	4499520	94.57	10.41	4.5	2.01	685244736	685118592	99.98	0.04	94.59	88.35	43.57
43655LF	789192576	789062688	99.98	0.04	94.4	88.04	43.29	5479602	5182137	94.57	11.1	4.95	2.18	789192576	789062688	99.98	0.04	94.4	88.04	43.29
1085LF	780118848	779982048	99.98	0.04	94.29	87.65	43.64	5416542	5119379	94.51	11.09	5.02	2.14	780118848	779982048	99.98	0.04	94.29	87.65	43.64
94240LF	817058304	816929856	99.98	0.04	94.46	88.08	43.21	5673124	5365622	94.58	11.66	4.74	2.2	817058304	816929856	99.98	0.04	94.46	88.08	43.21
58279LF	853392672	853362432	100	0.05	92.47	85	43.17	5926128	5563877	93.89	11.6	4.8	2.29	853392672	853362432	100	0.05	92.47	85	43.17
89019LF	801082944	800939520	99.98	0.02	96.84	92.63	42.05	5562080	5277469	94.88	10.89	5.2	2.26	801082944	800939520	99.98	0.02	96.84	92.63	42.05
89528LF	842039424	841871808	99.98	0.02	97.05	93.2	42.37	5846332	5542437	94.8	11.3	5.12	2.33	842039424	841871808	99.98	0.02	97.05	93.2	42.37
84628LF	914274432	914076000	99.98	0.01	97.43	94.19	42.39	6347750	6019948	94.84	11.54	5.65	2.45	914274432	914076000	99.98	0.01	97.43	94.19	42.39
94238LF	819279936	819110016	99.98	0.02	97.31	93.85	42.72	5688264	5395150	94.85	10.81	5.22	2.34	819279936	819110016	99.98	0.02	97.31	93.85	42.72
73062LF	609566400	609509664	99.99	0.02	97.12	93.34	41.47	4232706	4005634	94.64	9.43	4.93	1.83	609566400	609509664	99.99	0.02	97.12	93.34	41.47
93524LF	518165856	518115456	99.99	0.02	97.1	93.34	42.69	3598024	3412152	94.83	7.4	5.29	1.78	518165856	518115456	99.99	0.02	97.1	93.34	42.69
38842LF	796795200	796589856	99.97	0.01	97.45	94.48	42.06	5531874	5257144	95.03	11.17	4.64	2.28	796795200	796589856	99.97	0.01	97.45	94.48	42.06
27438LF	628051968	627897888	99.98	0.01	97.41	94.34	41.1	4360402	4144583	95.05	9.39	4.77	1.97	628051968	627897888	99.98	0.01	97.41	94.34	41.1
38416LF	658565856	658420704	99.98	0.02	97.16	93.62	41.47	4572366	4340079	94.92	9.74	4.62	2.06	658565856	658420704	99.98	0.02	97.16	93.62	41.47
30418LF	659445408	659388096	99.99	0.02	97.53	94.47	42	4579084	4346052	94.91	10	4.88	1.96	659445408	659388096	99.99	0.02	97.53	94.47	42
37788LF	686118816	686031552	99.99	0.01	97.66	94.91	41.88	4764108	4523893	94.96	10.14	5	2.02	686118816	686031552	99.99	0.01	97.66	94.91	41.88
95474LF	696769920	696697632	99.99	0.01	97.66	94.84	41.91	4838178	4594150	94.96	10.4	4.84	2.03	696769920	696697632	99.99	0.01	97.66	94.84	41.91
39312LF	602292384	602233632	99.99	0.01	97.48	94.36	42.13	4182178	3970196	94.93	9.43	4.65	1.88	602292384	602233632	99.99	0.01	97.48	94.36	42.13
94259LF	554512032	554451552	99.99	0.01	97.5	94.63	41.34	3850358	3660399	95.07	8.84	4.68	1.77	554512032	554451552	99.99	0.01	97.5	94.63	41.34
96789LF	683270784	683214624	99.99	0.03	96.55	91.81	41.63	4744546	4510644	95.07	11.4	4.54	1.83	683270784	683214624	99.99	0.03	96.55	91.81	41.63
18117LF	525339072	525224448	99.98	0.02	97.15	93.77	42.03	3647392	3469516	95.12	8.34	4.23	1.85	525339072	525224448	99.98	0.02	97.15	93.77	42.03

10942LF	703250496	703047456	99.97	0.01	97.31	94.25	41.81	4882274	4643251	95.1	9.92	4.86	2.16	703250496	703047456	99.97	0.01	97.31	94.25	41.81
19082LF	751876128	751678848	99.97	0.01	97.4	94.38	42.13	5219992	4958477	94.99	10.5	4.72	2.25	751876128	751678848	99.97	0.01	97.4	94.38	42.13
27334LF	682809120	682688448	99.98	0.02	97.13	93.65	41.64	4740892	4506626	95.06	9.87	4.86	2.09	682809120	682688448	99.98	0.02	97.13	93.65	41.64
78140LF	1772431488	1772405280	100	0.02	96.69	92.63	41.38	12308370	11655037	94.69	20.73	7.3	2.78	1772431488	1772405280	100	0.02	96.69	92.63	41.38
29789LF	1488945600	1488919680	100	0.01	96.81	92.92	42.12	10339720	9795661	94.74	18.98	5.73	2.62	1488945600	1488919680	100	0.01	96.81	92.92	42.12
39309LF	1247731488	1247710176	100	0.01	96.67	92.73	43.06	8664654	8204280	94.69	15.69	5.62	2.61	1247731488	1247710176	100	0.01	96.67	92.73	43.06
89317LF	608078016	608067936	100	0.01	96.89	93.03	43.83	4222694	3995431	94.62	9.42	4.79	1.86	608078016	608067936	100	0.01	96.89	93.03	43.83
345LF	670083840	669964032	99.98	0.01	97.46	94.51	41.15	4652528	4420229	95.01	10.9	4.01	1.95	670083840	669964032	99.98	0.01	97.46	94.51	41.15
54851LF	801618336	801487872	99.98	0.01	97.62	94.8	40.88	5565888	5284319	94.94	12.22	4.98	2.02	801618336	801487872	99.98	0.01	97.62	94.8	40.88
3528LF	740827296	740798784	100	0.03	95.97	90.46	42.05	5144436	4885765	94.97	10.05	5.9	2.08	740827296	740798784	100	0.03	95.97	90.46	42.05
1336LF	857028096	856989504	100	0.03	96.41	91.6	41.47	5951316	5648356	94.91	11.21	6.13	2.23	857028096	856989504	100	0.03	96.41	91.6	41.47
26705LF	541579104	541558944	100	0.03	96.5	91.8	41.83	3760826	3568512	94.89	8.9	4.18	1.8	541579104	541558944	100	0.03	96.5	91.8	41.83
3520LF	869394240	869355360	100	0.03	96.47	91.67	42.25	6037190	5725559	94.84	11.75	5.5	2.27	869394240	869355360	100	0.03	96.47	91.67	42.25
89314LF	898977888	898939008	100	0.03	96.38	91.51	42	6242632	5924269	94.9	12.82	4.96	2.23	898977888	898939008	100	0.03	96.38	91.51	42
95477LF	1129445856	1129133376	99.97	0.02	97.16	93.79	40.26	7841204	7454622	95.07	13.28	6.93	2.58	1129445856	1129133376	99.97	0.02	97.16	93.79	40.26
94283LF	1018514304	1018213344	99.97	0.02	97.08	93.66	42.08	7070926	6715861	94.98	12.12	5.7	2.68	1018514304	1018213344	99.97	0.02	97.08	93.66	42.08
99897LF	1138447296	1138119840	99.97	0.02	96.97	93.47	41.7	7903610	7512874	95.06	12.88	6.46	2.78	1138447296	1138119840	99.97	0.02	96.97	93.47	41.7
93487LF	1284552576	1284139296	99.97	0.01	97.16	93.9	41.55	8917634	8460745	94.88	13.99	6.74	2.91	1284552576	1284139296	99.97	0.01	97.16	93.9	41.55
9829LF	1278286848	1277844480	99.97	0.01	97.17	94.06	41.51	8873920	8442652	95.14	14.44	6.09	2.89	1278286848	1277844480	99.97	0.01	97.17	94.06	41.51
89647LF	1312146144	1311711552	99.97	0.01	97.22	94.08	41.27	9109108	8660902	95.08	14.94	5.99	2.89	1312146144	1311711552	99.97	0.01	97.22	94.08	41.27
8579LF	1367140320	1366682688	99.97	0.01	97.18	93.98	41.08	9490852	9020676	95.05	14.31	7.41	2.99	1367140320	1366682688	99.97	0.01	97.18	93.98	41.08
84912LF	1385182368	1384722720	99.97	0.01	97.16	94.03	41.44	9616130	9156503	95.22	13.89	7.81	3.09	1385182368	1384722720	99.97	0.01	97.16	94.03	41.44
84495LF	1394716032	1394231616	99.97	0.01	97.25	94.13	41.51	9682164	9207278	95.1	14.56	7.26	3.04	1394716032	1394231616	99.97	0.01	97.25	94.13	41.51
8444LF	1304020512	1303652160	99.97	0.02	96.91	93.25	41.05	9053140	8609675	95.1	14.22	7.09	2.88	1304020512	1303652160	99.97	0.02	96.91	93.25	41.05
81846LF	1217259648	1216877760	99.97	0.02	97.11	93.72	41.86	8450540	8030075	95.02	13.12	6.98	2.9	1217259648	1216877760	99.97	0.02	97.11	93.72	41.86
8155LF	1361986560	1361541600	99.97	0.01	97.31	94.2	41.89	9455150	8978910	94.96	14.38	6.78	3.05	1361986560	1361541600	99.97	0.01	97.31	94.2	41.89
58277LF	1426721472	1426316544	99.97	0.02	97.02	93.53	41.67	9904976	9408576	94.99	16.07	5.9	2.96	1426721472	1426316544	99.97	0.02	97.02	93.53	41.67
38722LF	1321848000	1321408512	99.97	0.01	97.24	94.16	41.65	9176448	8721004	95.04	14.23	6.63	2.99	1321848000	1321408512	99.97	0.01	97.24	94.16	41.65
18488LF	1283112864	1282656384	99.96	0.01	97.23	94.16	41.61	8907336	8461768	95	14.31	6.19	2.91	1283112864	1282656384	99.96	0.01	97.23	94.16	41.61
28312LF	1252004256	1251602208	99.97	0.01	97.22	94.05	41.95	8691682	8257806	95.01	13.82	6.42	2.91	1252004256	1251602208	99.97	0.01	97.22	94.05	41.95
28303LF	1110669696	1110325248	99.97	0.01	97.19	94.03	41.7	7710592	7328366	95.04	13.12	5.75	2.74	1110669696	1110325248	99.97	0.01	97.19	94.03	41.7
28087LF	1122635232	1122302880	99.97	0.02	97.01	93.53	41.91	7793770	7400034	94.95	13.06	5.93	2.76	1122635232	1122302880	99.97	0.02	97.01	93.53	41.91
27433LF	1059070464	1058771232	99.97	0.02	96.83	93.09	41.29	7352578	6990102	95.07	12.82	5.76	2.63	1059070464	1058771232	99.97	0.02	96.83	93.09	41.29
27382LF	1141960896	1141585920	99.97	0.01	97.25	94.11	41.03	7927680	7524259	94.91	13.74	5.7	2.69	1141960896	1141585920	99.97	0.01	97.25	94.11	41.03
27372LF	1030378464	1030070304	99.97	0.02	97.04	93.62	41.79	7153266	6799493	95.05	12.48	5.54	2.66	1030378464	1030070304	99.97	0.02	97.04	93.62	41.79
27318LF	807406272	807220224	99.98	0.02	96.22	91.69	41.16	5605696	5325770	95.01	10.71	5.28	2.31	807406272	807220224	99.98	0.02	96.22	91.69	41.16
27268LF	1014461856	1014135264	99.97	0.01	97.17	94	41.22	7042606	6696484	95.09	12.19	5.94	2.61	1014461856	1014135264	99.97	0.01	97.17	94	41.22
26899LF	1115894592	1115523936	99.97	0.01	97.16	93.94	41.53	7746694	7365198	95.08	13.13	5.95	2.72	1115894592	1115523936	99.97	0.01	97.16	93.94	41.53
26854LF	574796448	574782336	100	0.02	96.55	92.47	39.79	3991544	3788188	94.91	9.07	4.67	1.78	574796448	574782336	100	0.02	96.55	92.47	39.79
25985LF	793771488	793745568	100	0.02	96.51	92.5	41.42	5512122	5239522	95.05	9.4	5.49	2.57	793771488	793745568	100	0.02	96.51	92.5	41.42
