# Supplementary material for: Whole-genome sequence association study identifies cyclin dependent kinase 8 as a key gene for the number of mummified piglets
Source: Anim Biosci. 2022 Sep 7;36(1):29–42. doi: 10.5713/ab.22.0115 (PMC9834657; doi:10.5713/ab.22.0115)
Supplement: Supplementary file 2 [file ab-22-0115-suppl2.pdf]

Supplementary Table 2. Summary and mapping statistics of 40 Yorkshire pigs and 20 Landrace pigs using whole genome sequencing

Breed	Sample_ID	Read_Length(bp)	Clean_reads	Clean_bases(bp)	mapped_reads	mapped_bases(bp)	mismatch_bases(bp)	mismatch_ratio	mapping_ratio	dup_ratio	uniq_reads	Average_depth(x)	Coverage at least 1×	Coverage at least 4×
Landrace	L34001	150	335820428	50373064200	311801446	46213043692	557173208	1.21%	92.85%	6.39%	291888140	17.04	98.57%	96.21%
	L34005	150	334686080	50202912000	310680291	46015949213	586094437	1.27%	92.83%	6.65%	290028119	16.91	98.56%	96.18%
	L34603	150	335218524	50282778600	311014716	46064557161	587650239	1.28%	92.78%	6.79%	289892961	16.85	98.55%	95.89%
	L34903	150	334264202	50139630300	310274300	45980670807	560474193	1.22%	92.82%	6.45%	290262791	16.93	98.57%	96.19%
	L34904	150	336090594	50413589100	311470673	46129299352	591301598	1.28%	92.67%	6.10%	292475939	17.06	98.55%	96.11%
	L35703	150	336215912	50432386800	311639868	46142122393	603857807	1.31%	92.69%	5.69%	293910646	17.14	98.57%	96.17%
	L35709	150	336509542	50476431300	312576504	46278629530	607846070	1.31%	92.89%	5.56%	295201693	17.21	98.56%	96.07%
	L36007	150	335062848	50259427200	310393272	45978056243	580934557	1.26%	92.64%	5.92%	292025591	17.03	98.57%	96.14%
	L36008	150	334348478	50152271700	311286801	46100881725	592138425	1.28%	93.10%	5.99%	292625738	17.05	98.56%	96.18%
	L37603	150	336869760	50530464000	314477028	46553790399	617763801	1.33%	93.35%	6.25%	294810023	17.17	98.55%	96.30%
	L37606	150	334636164	50195424600	312071401	46220289404	590420746	1.28%	93.26%	6.30%	292410337	17.05	98.56%	96.47%
	L37607	150	336211362	50431704300	313229893	46374713620	609770330	1.31%	93.16%	6.34%	293362969	17.05	98.53%	96.15%
	L38603	150	335032912	50254936800	310860469	46002233815	626836535	1.36%	92.79%	5.91%	292495718	16.93	98.52%	95.23%
	L38605	150	335861790	50379268500	311821288	46178303730	594889470	1.29%	92.84%	6.20%	292501726	17.04	98.53%	95.89%
	L38607	150	335606092	50340913800	311530113	46123435613	606081337	1.31%	92.83%	5.78%	293529580	17.13	98.54%	96.42%
	L38609	150	336254460	50438169000	312381603	46279717422	577523028	1.25%	92.90%	5.48%	295249582	17.25	98.53%	96.40%
	L38801	150	335806734	50371010100	312014212	46203728968	598402832	1.30%	92.91%	4.76%	297162127	17.31	98.58%	96.23%
	L38805	150	334609234	50191385100	311015528	46069292174	583037026	1.27%	92.95%	4.39%	297365939	17.29	98.59%	96.17%
	L38806	150	335904976	50385746400	311902210	46187573116	597758384	1.29%	92.85%	4.90%	296609321	17.25	98.57%	96.09%
	L38809	150	335864038	50379605700	312347254	46268930315	583157785	1.26%	93.00%	5.74%	294432029	17.19	98.54%	96.43%
Yorkshire	Y30912	150	334467628	50170144200	310972473	46051495289	594375661	1.29%	92.98%	5.63%	293478123	17.01	98.56%	95.93%
	Y31016	150	337387992	50608198800	312843968	46303746604	622848596	1.35%	92.73%	5.34%	296142726	17.15	98.57%	96.08%
	Y31614	150	335321184	50298177600	313219783	46407023877	575943573	1.24%	93.41%	5.48%	296060462	17.28	98.54%	96.44%
	Y32118	150	336394912	50459236800	313959348	46516330502	577571698	1.24%	93.33%	4.68%	299263709	17.49	98.55%	96.39%
	Y32311	150	336709000	50506350000	314290343	46590752517	552798933	1.19%	93.34%	5.11%	298240605	17.43	98.57%	96.50%
	Y32813	150	335100268	50265040200	311084650	46038851437	623846063	1.36%	92.83%	6.01%	292389594	16.9	98.53%	95.65%
	Y33305	150	361849978	54277496700	334371801	49476182126	679588024	1.37%	92.41%	5.72%	315229211	18.12	98.37%	93.67%
	Y33411	150	337578512	50636776800	313559293	46436155000	597738950	1.29%	92.88%	5.85%	295208034	17.15	98.53%	95.58%
	Y33514	150	336999198	50549879700	314512520	46581858304	595019696	1.28%	93.33%	5.79%	296290962	17.21	98.52%	95.52%
	Y33614	150	337896516	50684477400	313811108	46513373490	558292710	1.20%	92.87%	7.00%	291840331	17.04	98.58%	96.45%
	Y35101	150	336342148	50451322200	310441931	45963925980	602363670	1.31%	92.30%	7.15%	288245164	16.73	98.49%	94.95%
	Y36102	150	334564368	50184655200	310701330	46065198503	540000997	1.17%	92.87%	8.15%	285384502	16.68	98.55%	96.10%
	Y36404	150	335286916	50293037400	310890994	46056981765	576667335	1.25%	92.72%	7.07%	288923585	16.8	98.52%	95.29%
	Y37313	150	335977606	50396640900	312026187	46226408885	577519165	1.25%	92.87%	7.09%	289890294	16.91	98.54%	96.30%
	Y37411	150	335768890	50365333500	311775319	46151020098	615277752	1.33%	92.85%	5.91%	293337239	16.97	98.49%	95.20%
	Y37703	150	336939116	50540867400	312474258	46291877514	579261186	1.25%	92.74%	5.08%	296610760	17.3	98.59%	96.27%
	Y37707	150	334101856	50115278400	310958445	46048927735	594839015	1.29%	93.07%	5.37%	294273742	17.12	98.54%	96.24%
	Y37813	150	336693222	50503983300	313682150	46409052235	643270265	1.39%	93.17%	5.16%	297488207	17.21	98.40%	94.06%
	Y37816	150	335686728	50353009200	313058740	46356804939	602006061	1.30%	93.26%	4.97%	297503682	17.34	98.55%	96.43%
	Y37902	150	336082608	50412391200	310400651	45970980822	589116828	1.28%	92.36%	4.74%	295695054	17.26	98.56%	96.20%
	Y38003	150	335586048	50337907200	311862781	46182712680	596704470	1.29%	92.93%	5.08%	296017605	17.29	98.57%	96.17%
	Y38206	150	336352898	50452934700	312576509	46300985662	585490688	1.26%	92.93%	5.90%	294120730	17.12	98.55%	96.38%
	Y38302	150	334773130	50215969500	311595698	46173259192	566095508	1.23%	93.08%	3.90%	299458168	17.52	98.57%	96.26%
	Y38310	150	336369520	50455428000	314239198	46524324033	611555667	1.31%	93.42%	4.88%	298909771	17.4	98.55%	95.98%
	Y38311	150	337731074	50659661100	313469121	46421006192	599361958	1.29%	92.82%	5.16%	297294443	17.3	98.53%	96.01%
	Y39017	150	336842146	50526321900	312042286	46195604210	610738690	1.32%	92.64%	6.68%	291196070	16.87	98.50%	95.47%
	Y39104	150	333604398	50040659700	309419813	45831582237	581389713	1.27%	92.75%	5.38%	292787355	17.08	98.56%	96.22%
	Y39113	150	334669750	50200462500	310530360	46012916181	566637819	1.23%	92.79%	6.16%	291390530	17.02	98.52%	96.36%
	Y39114	150	335564604	50334690600	314128701	46549666410	569638740	1.22%	93.61%	5.62%	296475671	17.35	98.54%	96.49%
	Y39115	150	335277436	50291615400	311870295	46196493194	584051056	1.26%	93.02%	5.73%	294015297	17.16	98.54%	96.38%
	Y39504	150	334914356	50237153400	310997483	46078427262	571195188	1.24%	92.86%	4.96%	295582310	17.28	98.56%	96.23%

	Y39505	150	335421566	50313234900	311265826	46105616446	584257454	1.27%	92.80%	5.02%	295628037	17.28	98.56%	96.16%
	Y39506	150	335061702	50259255300	309019821	45727128304	625844846	1.37%	92.23%	5.79%	291132748	16.84	98.48%	95.05%
	Y39510	150	336507496	50476124400	313561020	46403275616	630877384	1.36%	93.18%	5.78%	295446553	17.15	98.53%	95.61%
	Y39513	150	334747910	50212186500	313135552	46389875025	580457775	1.25%	93.54%	4.51%	299005123	17.5	98.55%	96.35%
	Y40303	150	335710784	50356617600	311036296	46105655337	549789063	1.19%	92.65%	8.53%	284516667	16.58	98.57%	96.11%
	Y40307	150	336738838	50510825700	312624823	46315929165	577794285	1.25%	92.84%	8.00%	287625379	16.74	98.54%	96.33%
	Y40310	150	334545294	50181794100	310728227	46037036961	572197089	1.24%	92.88%	8.12%	285511247	16.61	98.53%	96.08%
	Y40315	150	335266114	50289917100	311537428	46161998851	568615349	1.23%	92.92%	7.21%	289071674	16.84	98.63%	96.21%
	Y40412	150	336868670	50530300500	312980540	46391208927	555872073	1.20%	92.91%	7.55%	289344720	16.89	98.57%	96.35%
