# Supplementary material for: Whole-genome sequence association study identifies cyclin dependent kinase 8 as a key gene for the number of mummified piglets
Source: Anim Biosci. 2022 Sep 7;36(1):29–42. doi: 10.5713/ab.22.0115 (PMC9834657; doi:10.5713/ab.22.0115)
Supplement: Supplementary file 3 [file ab-22-0115-suppl3.pdf]

Supplemental Table S3. The primer information of mRNA

Gene name	Sequence(5' to 3')
CDK8	F-ACGTTTTTGCCGGTTGTCAG<br>R-TGATGAGTCCGCCTGAGGTA
YBX1	F-GAGAAGTGATGGAGGGTGCT<br>R-GGTTGTCTTTGGCGAGGAG
BCL-2	F-GATCCAGGATAACGGAGGCT<br>R-CCAGGAGAAATCAAATAGAGGC
BAX	F-CCTTTTGCTTCAGGGTTTCA<br>R-GTTACTGTCCAGTTCATCTCCAAT
GAPDH	F-TGACCCCTTCATTGACCTCC<br>R-TTCTCCGCCTTGACTGTGC

Supplemental Table S4. shRNA sequence and primer information

Gene	sequence (5'—3')
YBX1	474F-CACCGGAGTTTGATGTTGTTGAAGGTTCAAGAGACCTTCAACAACATCAAACTCCTTTTTTG
