# Supplementary material for: Whole-genome sequence association study identifies cyclin dependent kinase 8 as a key gene for the number of mummified piglets
Source: Anim Biosci. 2022 Sep 7;36(1):29–42. doi: 10.5713/ab.22.0115 (PMC9834657; doi:10.5713/ab.22.0115)
Supplement: Supplementary file 4 [file ab-22-0115-suppl4.pdf]

Supplemental Table S3. The primer information of mRNA

Gene name	Sequence(5' to 3')
CDK8	F-ACGTTTTTGCCGGTTGTCAG
	R-TGATGAGTCCGCCTGAGGTA
YBX1	F-GAGAAGTGATGGAGGGTGCT
	R-GGTTGTCTTTGGCGAGGAG
BCL-2	F-GATCCAGGATAACGGAGGCT
	R-CCAGGAGAAATCAAATAGAGGC
BAX	F-CCTTTTGCTTCAGGGTTTCA
	R-GTTACTGTCCAGTTCATCTCCAAT
GAPDH	F-TGACCCCTTCATTGACCTCC
	R-TTCTCCGCCTTGACTGTGC

Supplemental Table S4. shRNA sequence and primer information

Gene	sequence (5'—3')
YBX1	474F-CACCGGAGTTTGATGTTGTTGAAGGTTCAAGAGACCTTCAACAACATCAAACTCCTTTTTTG

	474R- GATCCAAAAAAGGAGTTTGATGTTGTTGAAGGTCTCTTGAACCTTCAACAACATCAAACTCC
	614F- CACCGCAATTACCAGCAGAATTACCTTCAAGAGAGGTAATTCTGCTGGTAATTGCTTTTTTG
	614R- GATCCAAAAAAGCAATTACCAGCAGAATTACCTCTCTTGAAGGTAATTCTGCTGGTAATTGC
	986F- CACCGGTACCGCCGCAACTTCAATTTCAAGAGAATTGAAGTTGCGGCGGTACCTTTTTTG
	986R- GATCCAAAAAAGGTACCGCCGCAACTTCAATTCTCTTGAAATTGAAGTTGCGGCGGTACC
Negative control	F- CACCGTTCTCCGAACGTGTCACGTTTCAAGAGAACGTGACACGTTCGGAGAATTTTTTG
	R- GATCCAAAAAATTCTCCGAACGTGTCACGTTCTCTTGAAACGTGACACGTTCGGAGAAC

Supplemental Table S5. Imputation accuracy and the number of SNPs calculated for each chromosome.

Chromosome	Before quality control		After quality control	
	Number of SNPs	Average accuracy	Number of SNPs	Average accuracy
1	1,356,375	0.71	477,856	0.97
2	1,052,192	0.71	303,923	0.96
3	866,093	0.71	300,641	0.97
4	783,586	0.72	274,087	0.96
5	691,083	0.72	183,287	0.96
6	943,353	0.72	301,818	0.96
7	844,634	0.73	229,335	0.96
8	900,494	0.73	294,289	0.96
9	892,768	0.73	300,569	0.96
