# Supplementary material for: Whole-genome sequence association study identifies cyclin dependent kinase 8 as a key gene for the number of mummified piglets
Source: Anim Biosci. 2022 Sep 7;36(1):29–42. doi: 10.5713/ab.22.0115 (PMC9834657; doi:10.5713/ab.22.0115)
Supplement: Supplementary file 6 [file ab-22-0115-suppl6.pdf]

Supplemental Table 6.Genome-wide significant and suggestive SNPs for NM in Landrace pigs

Chromosome	Position (bp)	n_miss	Allele	MAF	SNP effect	SE	*P* -value
1	141815233	0	C/T	0.014	0.61	0.09	8.96E-11
1	141816776	0	C/T	0.014	0.61	0.09	8.96E-11
1	133007748	0	C/T	0.011	0.76	0.12	1.30E-09
1	141810850	0	G/A	0.017	0.52	0.08	3.55E-09
1	141810910	0	G/T	0.017	0.52	0.08	3.55E-09
1	141810985	0	C/T	0.017	0.52	0.08	3.55E-09
1	141811143	0	C/T	0.017	0.52	0.08	3.55E-09
1	141811876	0	G/A	0.017	0.52	0.08	3.55E-09
1	141811910	0	C/A	0.017	0.52	0.08	3.55E-09
1	141812177	0	C/T	0.017	0.52	0.08	3.55E-09
1	141812373	0	T/C	0.017	0.52	0.08	3.55E-09
1	141812537	0	A/G	0.017	0.52	0.08	3.55E-09
1	141812610	0	G/A	0.017	0.52	0.08	3.55E-09
1	141813176	0	G/A	0.017	0.52	0.08	3.55E-09
1	141814081	0	T/C	0.017	0.52	0.08	3.55E-09
1	141814836	0	A/C	0.017	0.52	0.08	3.55E-09
1	141815148	0	A/G	0.017	0.52	0.08	3.55E-09
1	141815633	0	C/T	0.017	0.52	0.08	3.55E-09
1	141815918	0	A/C	0.017	0.52	0.08	3.55E-09
1	141816093	0	C/T	0.017	0.52	0.08	3.55E-09
1	141816230	0	C/T	0.017	0.52	0.08	3.55E-09
1	141816551	0	G/A	0.017	0.52	0.08	3.55E-09
1	141816841	0	G/C	0.017	0.52	0.08	3.55E-09
1	141816878	0	C/T	0.017	0.52	0.08	3.55E-09
1	141818059	0	T/C	0.017	0.52	0.08	3.55E-09
1	141818270	0	T/C	0.017	0.52	0.08	3.55E-09
1	141818436	0	C/T	0.017	0.52	0.08	3.55E-09
1	141822642	0	T/C	0.017	0.52	0.08	3.55E-09
1	141840538	0	T/C	0.017	0.52	0.08	3.55E-09
1	141840645	0	C/G	0.017	0.52	0.08	3.55E-09
1	141901429	0	A/G	0.017	0.52	0.08	3.55E-09
11	1889658	0	T/C	0.011	0.87	0.11	9.98E-13
11	7114076	0	A/G	0.022	0.56	0.07	1.35E-12
11	2446903	0	G/C	0.011	0.86	0.12	3.41E-12
11	2446909	0	T/A	0.011	0.86	0.12	3.41E-12
11	2447201	0	T/C	0.011	0.86	0.12	3.41E-12
11	1301414	0	G/T	0.034	0.40	0.06	3.02E-10

11	1301444	0	T/C	0.034	0.40	0.06	3.02E-10
11	1304274	0	G/A	0.034	0.40	0.06	3.02E-10
11	1307566	0	A/G	0.034	0.40	0.06	3.02E-10
11	1429400	0	G/C	0.075	0.30	0.05	4.26E-10
11	1448703	0	G/T	0.075	0.30	0.05	4.26E-10
11	1448736	0	T/G	0.075	0.30	0.05	4.26E-10
11	2633207	0	T/C	0.017	0.62	0.10	1.43E-09
11	1273376	0	A/G	0.073	0.29	0.05	1.51E-09
11	1273583	0	T/C	0.073	0.29	0.05	1.51E-09
11	1274094	0	C/T	0.073	0.29	0.05	1.51E-09
11	1274544	0	T/G	0.073	0.29	0.05	1.51E-09
11	1274587	0	A/C	0.073	0.29	0.05	1.51E-09
11	1275008	0	C/G	0.073	0.29	0.05	1.51E-09
11	1275048	0	C/T	0.073	0.29	0.05	1.51E-09
11	1275356	0	A/G	0.073	0.29	0.05	1.51E-09
11	1275384	0	T/C	0.073	0.29	0.05	1.51E-09
11	1275411	0	T/C	0.073	0.29	0.05	1.51E-09
11	1275451	0	G/C	0.073	0.29	0.05	1.51E-09
11	1275460	0	T/C	0.073	0.29	0.05	1.51E-09
11	1275492	0	G/A	0.073	0.29	0.05	1.51E-09
11	1275978	0	G/A	0.073	0.29	0.05	1.51E-09
11	1275987	0	G/C	0.073	0.29	0.05	1.51E-09
11	1277503	0	C/T	0.073	0.29	0.05	1.51E-09
11	1278303	0	G/A	0.073	0.29	0.05	1.51E-09
11	1279289	0	C/A	0.073	0.29	0.05	1.51E-09
11	1279649	0	A/G	0.073	0.29	0.05	1.51E-09
11	1280116	0	G/A	0.073	0.29	0.05	1.51E-09
11	1280466	0	G/C	0.073	0.29	0.05	1.51E-09
11	1280644	0	A/G	0.073	0.29	0.05	1.51E-09
11	1281303	0	C/T	0.073	0.29	0.05	1.51E-09
11	1281440	0	T/A	0.073	0.29	0.05	1.51E-09
11	1284356	0	T/C	0.073	0.29	0.05	1.51E-09
11	1285805	0	T/C	0.073	0.29	0.05	1.51E-09
11	1287264	0	A/G	0.073	0.29	0.05	1.51E-09
11	1288193	0	C/G	0.073	0.29	0.05	1.51E-09
11	1288731	0	T/G	0.073	0.29	0.05	1.51E-09
11	1289254	0	C/G	0.073	0.29	0.05	1.51E-09
11	1289594	0	C/G	0.073	0.29	0.05	1.51E-09
11	1289702	0	G/A	0.073	0.29	0.05	1.51E-09
11	1289735	0	A/G	0.073	0.29	0.05	1.51E-09

11	1289956	0	A/G	0.073	0.29	0.05	1.51E-09
11	1290245	0	C/T	0.073	0.29	0.05	1.51E-09
11	1290348	0	C/T	0.073	0.29	0.05	1.51E-09
11	1290971	0	G/A	0.073	0.29	0.05	1.51E-09
11	1291081	0	A/G	0.073	0.29	0.05	1.51E-09
11	1291105	0	T/C	0.073	0.29	0.05	1.51E-09
11	1291115	0	A/G	0.073	0.29	0.05	1.51E-09
11	1291156	0	G/T	0.073	0.29	0.05	1.51E-09
11	1291903	0	C/T	0.073	0.29	0.05	1.51E-09
11	1291910	0	T/G	0.073	0.29	0.05	1.51E-09
11	1291952	0	C/T	0.073	0.29	0.05	1.51E-09
11	1291959	0	T/C	0.073	0.29	0.05	1.51E-09
11	1291963	0	G/A	0.073	0.29	0.05	1.51E-09
11	1292697	0	G/A	0.073	0.29	0.05	1.51E-09
11	1292969	0	T/C	0.073	0.29	0.05	1.51E-09
11	1293428	0	A/G	0.073	0.29	0.05	1.51E-09
11	1293479	0	A/T	0.073	0.29	0.05	1.51E-09
11	1293528	0	T/C	0.073	0.29	0.05	1.51E-09
11	1293532	0	C/G	0.073	0.29	0.05	1.51E-09
11	1293689	0	C/T	0.073	0.29	0.05	1.51E-09
11	1294087	0	A/G	0.073	0.29	0.05	1.51E-09
11	1294296	0	T/C	0.073	0.29	0.05	1.51E-09
11	1295174	0	G/A	0.073	0.29	0.05	1.51E-09
11	1295271	0	C/T	0.073	0.29	0.05	1.51E-09
11	1295385	0	G/A	0.073	0.29	0.05	1.51E-09
11	1295497	0	A/G	0.073	0.29	0.05	1.51E-09
11	1295592	0	C/T	0.073	0.29	0.05	1.51E-09
11	1295597	0	A/G	0.073	0.29	0.05	1.51E-09
11	1295687	0	T/C	0.073	0.29	0.05	1.51E-09
11	1295880	0	G/A	0.073	0.29	0.05	1.51E-09
11	1296208	0	A/G	0.073	0.29	0.05	1.51E-09
11	1296224	0	G/A	0.073	0.29	0.05	1.51E-09
11	1296245	0	G/T	0.073	0.29	0.05	1.51E-09
11	1296342	0	G/A	0.073	0.29	0.05	1.51E-09
11	1296689	0	T/C	0.073	0.29	0.05	1.51E-09
11	1296807	0	C/T	0.073	0.29	0.05	1.51E-09
11	1296960	0	A/G	0.073	0.29	0.05	1.51E-09
11	1296962	0	G/A	0.073	0.29	0.05	1.51E-09
11	1297006	0	G/A	0.073	0.29	0.05	1.51E-09
11	1297238	0	A/G	0.073	0.29	0.05	1.51E-09

11	1297367	0	G/A	0.073	0.29	0.05	1.51E-09
11	1297513	0	C/T	0.073	0.29	0.05	1.51E-09
11	1297541	0	C/T	0.073	0.29	0.05	1.51E-09
11	1297626	0	T/C	0.073	0.29	0.05	1.51E-09
11	1297676	0	G/C	0.073	0.29	0.05	1.51E-09
11	1297680	0	C/A	0.073	0.29	0.05	1.51E-09
11	1297702	0	T/C	0.073	0.29	0.05	1.51E-09
11	1297707	0	T/C	0.073	0.29	0.05	1.51E-09
11	1297744	0	G/A	0.073	0.29	0.05	1.51E-09
11	1297750	0	C/T	0.073	0.29	0.05	1.51E-09
11	1297766	0	G/T	0.073	0.29	0.05	1.51E-09
11	1297793	0	A/G	0.073	0.29	0.05	1.51E-09
11	1297836	0	T/C	0.073	0.29	0.05	1.51E-09
11	1297942	0	A/T	0.073	0.29	0.05	1.51E-09
11	1297953	0	T/C	0.073	0.29	0.05	1.51E-09
11	1298161	0	A/G	0.073	0.29	0.05	1.51E-09
11	2880327	0	A/G	0.017	0.62	0.10	1.63E-09
11	2880330	0	C/T	0.017	0.62	0.10	1.63E-09
11	2880345	0	G/A	0.017	0.62	0.10	1.63E-09
11	2880431	0	G/A	0.017	0.62	0.10	1.63E-09
11	2880440	0	A/G	0.017	0.62	0.10	1.63E-09
11	2316966	0	T/C	0.014	0.67	0.11	1.87E-09
11	2319201	0	T/C	0.014	0.67	0.11	1.87E-09
11	2366348	0	G/A	0.014	0.67	0.11	1.87E-09
11	2366540	0	C/T	0.014	0.67	0.11	1.87E-09
11	2366641	0	A/C	0.014	0.67	0.11	1.87E-09
11	2367474	0	G/A	0.014	0.67	0.11	1.87E-09
11	2367507	0	A/C	0.014	0.67	0.11	1.87E-09
11	2367648	0	A/G	0.014	0.67	0.11	1.87E-09
11	2367656	0	C/A	0.014	0.67	0.11	1.87E-09
11	2368223	0	A/G	0.014	0.67	0.11	1.87E-09
11	2368553	0	C/G	0.014	0.67	0.11	1.87E-09
11	2368568	0	A/G	0.014	0.67	0.11	1.87E-09
11	2368605	0	G/A	0.014	0.67	0.11	1.87E-09
11	2368664	0	G/A	0.014	0.67	0.11	1.87E-09
11	2368709	0	A/G	0.014	0.67	0.11	1.87E-09
11	2368766	0	A/G	0.014	0.67	0.11	1.87E-09
11	2368782	0	C/T	0.014	0.67	0.11	1.87E-09
11	2368794	0	G/A	0.014	0.67	0.11	1.87E-09
11	2369148	0	A/G	0.014	0.67	0.11	1.87E-09

11	2369182	0	A/G	0.014	0.67	0.11	1.87E-09
11	2369353	0	T/C	0.014	0.67	0.11	1.87E-09
11	2369373	0	T/C	0.014	0.67	0.11	1.87E-09
11	2404235	0	G/T	0.014	0.67	0.11	1.87E-09
11	2404304	0	A/G	0.014	0.67	0.11	1.87E-09
11	2404343	0	T/C	0.014	0.67	0.11	1.87E-09
11	2404478	0	T/C	0.014	0.67	0.11	1.87E-09
11	2408171	0	A/G	0.014	0.67	0.11	1.87E-09
11	2408537	0	T/G	0.014	0.67	0.11	1.87E-09
11	2408650	0	T/C	0.014	0.67	0.11	1.87E-09
11	2408738	0	C/T	0.014	0.67	0.11	1.87E-09
11	2408745	0	T/C	0.014	0.67	0.11	1.87E-09
11	2408813	0	C/G	0.014	0.67	0.11	1.87E-09
11	2411359	0	T/G	0.014	0.67	0.11	1.87E-09
11	2411370	0	A/G	0.014	0.67	0.11	1.87E-09
11	2411390	0	T/G	0.014	0.67	0.11	1.87E-09
11	2412314	0	C/T	0.014	0.67	0.11	1.87E-09
11	2413175	0	T/C	0.014	0.67	0.11	1.87E-09
11	2413485	0	G/A	0.014	0.67	0.11	1.87E-09
11	2442406	0	A/T	0.014	0.67	0.11	1.87E-09
11	2442486	0	C/G	0.014	0.67	0.11	1.87E-09
11	2442487	0	C/G	0.014	0.67	0.11	1.87E-09
11	2444048	0	T/G	0.014	0.67	0.11	1.87E-09
11	2444263	0	T/A	0.014	0.67	0.11	1.87E-09
11	2444286	0	T/C	0.014	0.67	0.11	1.87E-09
11	2444294	0	A/G	0.014	0.67	0.11	1.87E-09
11	2444404	0	A/G	0.014	0.67	0.11	1.87E-09
11	2444459	0	A/C	0.014	0.67	0.11	1.87E-09
11	2444844	0	A/G	0.014	0.67	0.11	1.87E-09
11	2446842	0	A/T	0.014	0.67	0.11	1.87E-09
11	2446880	0	T/C	0.014	0.67	0.11	1.87E-09
11	2486317	0	A/G	0.014	0.67	0.11	1.87E-09
11	2503303	0	G/A	0.014	0.67	0.11	1.87E-09
11	2503313	0	C/T	0.014	0.67	0.11	1.87E-09
11	2503476	0	G/C	0.014	0.67	0.11	1.87E-09
11	2504436	0	C/A	0.014	0.67	0.11	1.87E-09
11	2504583	0	A/G	0.014	0.67	0.11	1.87E-09
11	2504585	0	C/T	0.014	0.67	0.11	1.87E-09
11	2577311	0	T/C	0.014	0.67	0.11	1.87E-09
11	2577371	0	A/G	0.014	0.67	0.11	1.87E-09

11	2577589	0	C/T	0.014	0.67	0.11	1.87E-09
11	2577711	0	T/C	0.014	0.67	0.11	1.87E-09
11	2578408	0	G/A	0.014	0.67	0.11	1.87E-09
11	2578410	0	A/G	0.014	0.67	0.11	1.87E-09
11	2579103	0	A/G	0.014	0.67	0.11	1.87E-09
11	2579157	0	T/C	0.014	0.67	0.11	1.87E-09
11	2579267	0	A/T	0.014	0.67	0.11	1.87E-09
11	2579271	0	A/G	0.014	0.67	0.11	1.87E-09
11	2580920	0	G/T	0.014	0.67	0.11	1.87E-09
11	2581451	0	C/G	0.014	0.67	0.11	1.87E-09
11	2581619	0	C/T	0.014	0.67	0.11	1.87E-09
11	2582211	0	G/C	0.014	0.67	0.11	1.87E-09
11	2582338	0	T/G	0.014	0.67	0.11	1.87E-09
11	2582972	0	A/G	0.014	0.67	0.11	1.87E-09
11	2583127	0	G/A	0.014	0.67	0.11	1.87E-09
11	2583945	0	G/C	0.014	0.67	0.11	1.87E-09
11	2583946	0	G/A	0.014	0.67	0.11	1.87E-09
11	2584179	0	T/C	0.014	0.67	0.11	1.87E-09
11	2584216	0	T/C	0.014	0.67	0.11	1.87E-09
11	2584222	0	C/T	0.014	0.67	0.11	1.87E-09
11	2584290	0	T/C	0.014	0.67	0.11	1.87E-09
11	2584293	0	A/G	0.014	0.67	0.11	1.87E-09
11	2584358	0	T/G	0.014	0.67	0.11	1.87E-09
11	2584679	0	T/C	0.014	0.67	0.11	1.87E-09
11	2584930	0	A/C	0.014	0.67	0.11	1.87E-09
11	2584937	0	G/T	0.014	0.67	0.11	1.87E-09
11	2585061	0	G/C	0.014	0.67	0.11	1.87E-09
11	2585074	0	T/C	0.014	0.67	0.11	1.87E-09
11	2585075	0	G/A	0.014	0.67	0.11	1.87E-09
11	2585080	0	G/A	0.014	0.67	0.11	1.87E-09
11	2585118	0	C/T	0.014	0.67	0.11	1.87E-09
11	2585128	0	C/T	0.014	0.67	0.11	1.87E-09
11	2585145	0	A/G	0.014	0.67	0.11	1.87E-09
11	2585169	0	T/C	0.014	0.67	0.11	1.87E-09
11	2585231	0	C/T	0.014	0.67	0.11	1.87E-09
11	2585249	0	C/G	0.014	0.67	0.11	1.87E-09
11	2585730	0	T/C	0.014	0.67	0.11	1.87E-09
11	2585781	0	T/C	0.014	0.67	0.11	1.87E-09
11	2585972	0	G/A	0.014	0.67	0.11	1.87E-09
11	2585985	0	A/G	0.014	0.67	0.11	1.87E-09

11	2586114	0	C/T	0.014	0.67	0.11	1.87E-09
11	2586326	0	A/G	0.014	0.67	0.11	1.87E-09
11	2586425	0	G/T	0.014	0.67	0.11	1.87E-09
11	2587480	0	C/A	0.014	0.67	0.11	1.87E-09
11	2587956	0	C/T	0.014	0.67	0.11	1.87E-09
11	2588017	0	T/C	0.014	0.67	0.11	1.87E-09
11	2588650	0	G/A	0.014	0.67	0.11	1.87E-09
11	2591308	0	A/G	0.014	0.67	0.11	1.87E-09
11	2591831	0	A/G	0.014	0.67	0.11	1.87E-09
11	2591842	0	C/T	0.014	0.67	0.11	1.87E-09
11	2591890	0	T/C	0.014	0.67	0.11	1.87E-09
11	2591894	0	T/G	0.014	0.67	0.11	1.87E-09
11	2591911	0	C/T	0.014	0.67	0.11	1.87E-09
11	2592376	0	T/C	0.014	0.67	0.11	1.87E-09
11	2592692	0	C/T	0.014	0.67	0.11	1.87E-09
11	2592773	0	G/A	0.014	0.67	0.11	1.87E-09
11	2592877	0	G/A	0.014	0.67	0.11	1.87E-09
11	2593126	0	G/A	0.014	0.67	0.11	1.87E-09
11	2593540	0	C/T	0.014	0.67	0.11	1.87E-09
11	2593617	0	A/G	0.014	0.67	0.11	1.87E-09
11	2594352	0	A/T	0.014	0.67	0.11	1.87E-09
11	2594598	0	C/A	0.014	0.67	0.11	1.87E-09
11	2594600	0	T/A	0.014	0.67	0.11	1.87E-09
11	2594750	0	C/T	0.014	0.67	0.11	1.87E-09
11	2595789	0	G/T	0.014	0.67	0.11	1.87E-09
11	2596040	0	T/C	0.014	0.67	0.11	1.87E-09
11	2596093	0	A/G	0.014	0.67	0.11	1.87E-09
11	2596161	0	A/G	0.014	0.67	0.11	1.87E-09
11	2596836	0	G/A	0.014	0.67	0.11	1.87E-09
11	2597165	0	A/G	0.014	0.67	0.11	1.87E-09
11	2597189	0	G/A	0.014	0.67	0.11	1.87E-09
11	2597263	0	T/C	0.014	0.67	0.11	1.87E-09
11	2597426	0	T/G	0.014	0.67	0.11	1.87E-09
11	2597496	0	C/G	0.014	0.67	0.11	1.87E-09
11	2597528	0	A/G	0.014	0.67	0.11	1.87E-09
11	2600006	0	C/T	0.014	0.67	0.11	1.87E-09
11	2600499	0	C/T	0.014	0.67	0.11	1.87E-09
11	2600526	0	A/G	0.014	0.67	0.11	1.87E-09
11	2601209	0	C/T	0.014	0.67	0.11	1.87E-09
11	2601241	0	G/A	0.014	0.67	0.11	1.87E-09

11	2601255	0	A/G	0.014	0.67	0.11	1.87E-09
11	2601303	0	T/G	0.014	0.67	0.11	1.87E-09
11	2601418	0	T/A	0.014	0.67	0.11	1.87E-09
11	2601647	0	C/T	0.014	0.67	0.11	1.87E-09
11	2601725	0	A/G	0.014	0.67	0.11	1.87E-09
11	2602061	0	A/T	0.014	0.67	0.11	1.87E-09
11	2602115	0	T/C	0.014	0.67	0.11	1.87E-09
11	2602146	0	T/C	0.014	0.67	0.11	1.87E-09
11	2602977	0	A/G	0.014	0.67	0.11	1.87E-09
11	2603507	0	G/A	0.014	0.67	0.11	1.87E-09
11	2603675	0	T/G	0.014	0.67	0.11	1.87E-09
11	2605956	0	A/G	0.014	0.67	0.11	1.87E-09
11	2606046	0	A/C	0.014	0.67	0.11	1.87E-09
11	2606117	0	A/G	0.014	0.67	0.11	1.87E-09
11	2606147	0	A/G	0.014	0.67	0.11	1.87E-09
11	2606154	0	A/G	0.014	0.67	0.11	1.87E-09
11	2606186	0	G/T	0.014	0.67	0.11	1.87E-09
11	2606197	0	C/G	0.014	0.67	0.11	1.87E-09
11	2606432	0	C/T	0.014	0.67	0.11	1.87E-09
11	2606433	0	A/G	0.014	0.67	0.11	1.87E-09
11	2606545	0	A/G	0.014	0.67	0.11	1.87E-09
11	2607037	0	T/C	0.014	0.67	0.11	1.87E-09
11	2607511	0	A/C	0.014	0.67	0.11	1.87E-09
11	2608021	0	G/A	0.014	0.67	0.11	1.87E-09
11	2608180	0	G/T	0.014	0.67	0.11	1.87E-09
11	2608245	0	C/T	0.014	0.67	0.11	1.87E-09
11	2608445	0	C/T	0.014	0.67	0.11	1.87E-09
11	2608582	0	T/C	0.014	0.67	0.11	1.87E-09
11	2608877	0	T/C	0.014	0.67	0.11	1.87E-09
11	2608895	0	G/A	0.014	0.67	0.11	1.87E-09
11	2608943	0	A/T	0.014	0.67	0.11	1.87E-09
11	2609128	0	C/T	0.014	0.67	0.11	1.87E-09
11	2609166	0	G/A	0.014	0.67	0.11	1.87E-09
11	2609419	0	G/A	0.014	0.67	0.11	1.87E-09
11	2609483	0	A/C	0.014	0.67	0.11	1.87E-09
11	2609502	0	G/T	0.014	0.67	0.11	1.87E-09
11	2609528	0	G/T	0.014	0.67	0.11	1.87E-09
11	2609628	0	C/A	0.014	0.67	0.11	1.87E-09
11	2609640	0	A/C	0.014	0.67	0.11	1.87E-09
11	2609668	0	A/G	0.014	0.67	0.11	1.87E-09

11	2609916	0	G/A	0.014	0.67	0.11	1.87E-09
11	2609940	0	T/C	0.014	0.67	0.11	1.87E-09
11	2609961	0	G/T	0.014	0.67	0.11	1.87E-09
11	2610138	0	T/C	0.014	0.67	0.11	1.87E-09
11	2610160	0	G/A	0.014	0.67	0.11	1.87E-09
11	2610167	0	A/G	0.014	0.67	0.11	1.87E-09
11	2610232	0	A/T	0.014	0.67	0.11	1.87E-09
11	2610283	0	T/G	0.014	0.67	0.11	1.87E-09
11	2610322	0	C/T	0.014	0.67	0.11	1.87E-09
11	2610380	0	C/T	0.014	0.67	0.11	1.87E-09
11	2610422	0	C/G	0.014	0.67	0.11	1.87E-09
11	2610450	0	A/C	0.014	0.67	0.11	1.87E-09
11	2610457	0	T/A	0.014	0.67	0.11	1.87E-09
11	2610490	0	A/G	0.014	0.67	0.11	1.87E-09
11	2610536	0	G/A	0.014	0.67	0.11	1.87E-09
11	2610678	0	A/G	0.014	0.67	0.11	1.87E-09
11	2610698	0	G/C	0.014	0.67	0.11	1.87E-09
11	2610729	0	A/G	0.014	0.67	0.11	1.87E-09
11	2610792	0	T/C	0.014	0.67	0.11	1.87E-09
11	2610811	0	A/G	0.014	0.67	0.11	1.87E-09
11	2610854	0	T/C	0.014	0.67	0.11	1.87E-09
11	2610908	0	A/C	0.014	0.67	0.11	1.87E-09
11	2610921	0	T/G	0.014	0.67	0.11	1.87E-09
11	2610925	0	A/G	0.014	0.67	0.11	1.87E-09
11	2610933	0	T/C	0.014	0.67	0.11	1.87E-09
11	2610948	0	A/G	0.014	0.67	0.11	1.87E-09
11	2610957	0	T/C	0.014	0.67	0.11	1.87E-09
11	2610958	0	A/T	0.014	0.67	0.11	1.87E-09
11	2610996	0	A/T	0.014	0.67	0.11	1.87E-09
11	2610997	0	G/A	0.014	0.67	0.11	1.87E-09
11	2611045	0	A/T	0.014	0.67	0.11	1.87E-09
11	2611293	0	G/C	0.014	0.67	0.11	1.87E-09
11	2611414	0	G/A	0.014	0.67	0.11	1.87E-09
11	2611428	0	T/C	0.014	0.67	0.11	1.87E-09
11	2611463	0	A/T	0.014	0.67	0.11	1.87E-09
11	2611521	0	T/A	0.014	0.67	0.11	1.87E-09
11	2611539	0	T/C	0.014	0.67	0.11	1.87E-09
11	2611544	0	G/A	0.014	0.67	0.11	1.87E-09
11	2612160	0	C/G	0.014	0.67	0.11	1.87E-09
11	2612348	0	C/G	0.014	0.67	0.11	1.87E-09

11	2614523	0	T/A	0.014	0.67	0.11	1.87E-09
11	2614681	0	G/C	0.014	0.67	0.11	1.87E-09
11	2614859	0	T/C	0.014	0.67	0.11	1.87E-09
11	2614905	0	C/T	0.014	0.67	0.11	1.87E-09
11	2614910	0	A/G	0.014	0.67	0.11	1.87E-09
11	2614926	0	A/G	0.014	0.67	0.11	1.87E-09
11	2615037	0	T/C	0.014	0.67	0.11	1.87E-09
11	2615055	0	T/C	0.014	0.67	0.11	1.87E-09
11	2615075	0	C/T	0.014	0.67	0.11	1.87E-09
11	2615273	0	T/C	0.014	0.67	0.11	1.87E-09
11	2615293	0	C/T	0.014	0.67	0.11	1.87E-09
11	2615321	0	G/A	0.014	0.67	0.11	1.87E-09
11	2615325	0	C/T	0.014	0.67	0.11	1.87E-09
11	2615351	0	T/C	0.014	0.67	0.11	1.87E-09
11	2615375	0	G/C	0.014	0.67	0.11	1.87E-09
11	2615534	0	T/C	0.014	0.67	0.11	1.87E-09
11	2615579	0	T/C	0.014	0.67	0.11	1.87E-09
11	2615604	0	T/C	0.014	0.67	0.11	1.87E-09
11	2615614	0	T/C	0.014	0.67	0.11	1.87E-09
11	2615619	0	T/C	0.014	0.67	0.11	1.87E-09
11	2615691	0	C/A	0.014	0.67	0.11	1.87E-09
11	2615763	0	T/G	0.014	0.67	0.11	1.87E-09
11	2615814	0	C/T	0.014	0.67	0.11	1.87E-09
11	2615827	0	G/A	0.014	0.67	0.11	1.87E-09
11	2615913	0	G/A	0.014	0.67	0.11	1.87E-09
11	2615952	0	G/T	0.014	0.67	0.11	1.87E-09
11	2616007	0	A/C	0.014	0.67	0.11	1.87E-09
11	2616008	0	G/A	0.014	0.67	0.11	1.87E-09
11	2616038	0	G/A	0.014	0.67	0.11	1.87E-09
11	2616042	0	G/T	0.014	0.67	0.11	1.87E-09
11	2616050	0	G/A	0.014	0.67	0.11	1.87E-09
11	2616082	0	T/C	0.014	0.67	0.11	1.87E-09
11	2617597	0	A/G	0.014	0.67	0.11	1.87E-09
11	2617660	0	A/G	0.014	0.67	0.11	1.87E-09
11	2617683	0	T/C	0.014	0.67	0.11	1.87E-09
11	2618019	0	C/T	0.014	0.67	0.11	1.87E-09
11	2618093	0	C/T	0.014	0.67	0.11	1.87E-09
11	2618157	0	T/A	0.014	0.67	0.11	1.87E-09
11	2618556	0	A/G	0.014	0.67	0.11	1.87E-09
11	2618573	0	C/T	0.014	0.67	0.11	1.87E-09

11	2618607	0	T/A	0.014	0.67	0.11	1.87E-09
11	2618641	0	A/G	0.014	0.67	0.11	1.87E-09
11	2618697	0	C/A	0.014	0.67	0.11	1.87E-09
11	2618737	0	C/T	0.014	0.67	0.11	1.87E-09
11	2618783	0	T/C	0.014	0.67	0.11	1.87E-09
11	2618819	0	T/C	0.014	0.67	0.11	1.87E-09
11	2618837	0	T/C	0.014	0.67	0.11	1.87E-09
11	2618918	0	G/A	0.014	0.67	0.11	1.87E-09
11	2618950	0	T/C	0.014	0.67	0.11	1.87E-09
11	2619228	0	A/G	0.014	0.67	0.11	1.87E-09
11	2619486	0	A/G	0.014	0.67	0.11	1.87E-09
11	2619701	0	T/C	0.014	0.67	0.11	1.87E-09
11	2619946	0	A/G	0.014	0.67	0.11	1.87E-09
11	2619976	0	C/A	0.014	0.67	0.11	1.87E-09
11	2620085	0	C/T	0.014	0.67	0.11	1.87E-09
11	2620087	0	A/G	0.014	0.67	0.11	1.87E-09
11	2620107	0	T/C	0.014	0.67	0.11	1.87E-09
11	2620496	0	C/G	0.014	0.67	0.11	1.87E-09
11	2620538	0	A/G	0.014	0.67	0.11	1.87E-09
11	2620549	0	A/G	0.014	0.67	0.11	1.87E-09
11	2620888	0	A/G	0.014	0.67	0.11	1.87E-09
11	2621034	0	T/G	0.014	0.67	0.11	1.87E-09
11	2621232	0	T/C	0.014	0.67	0.11	1.87E-09
11	2622019	0	A/G	0.014	0.67	0.11	1.87E-09
11	2622183	0	C/T	0.014	0.67	0.11	1.87E-09
11	2622184	0	A/G	0.014	0.67	0.11	1.87E-09
11	2622187	0	A/G	0.014	0.67	0.11	1.87E-09
11	2622387	0	T/G	0.014	0.67	0.11	1.87E-09
11	2622388	0	G/T	0.014	0.67	0.11	1.87E-09
11	2622502	0	C/T	0.014	0.67	0.11	1.87E-09
11	2622960	0	G/A	0.014	0.67	0.11	1.87E-09
11	2623105	0	G/A	0.014	0.67	0.11	1.87E-09
11	2623467	0	A/G	0.014	0.67	0.11	1.87E-09
11	2623544	0	C/T	0.014	0.67	0.11	1.87E-09
11	2623719	0	T/C	0.014	0.67	0.11	1.87E-09
11	2623799	0	C/T	0.014	0.67	0.11	1.87E-09
11	2623853	0	T/G	0.014	0.67	0.11	1.87E-09
11	2623888	0	G/C	0.014	0.67	0.11	1.87E-09
11	2623947	0	A/C	0.014	0.67	0.11	1.87E-09
11	2624010	0	T/C	0.014	0.67	0.11	1.87E-09

11	2624013	0	T/G	0.014	0.67	0.11	1.87E-09
11	2624035	0	G/T	0.014	0.67	0.11	1.87E-09
11	2624068	0	A/G	0.014	0.67	0.11	1.87E-09
11	2624070	0	T/G	0.014	0.67	0.11	1.87E-09
11	2624237	0	A/G	0.014	0.67	0.11	1.87E-09
11	2624300	0	T/C	0.014	0.67	0.11	1.87E-09
11	2624353	0	G/T	0.014	0.67	0.11	1.87E-09
11	2624538	0	C/T	0.014	0.67	0.11	1.87E-09
11	2624624	0	A/G	0.014	0.67	0.11	1.87E-09
11	2625200	0	A/G	0.014	0.67	0.11	1.87E-09
11	2625202	0	A/G	0.014	0.67	0.11	1.87E-09
11	2625203	0	T/C	0.014	0.67	0.11	1.87E-09
11	2625222	0	C/T	0.014	0.67	0.11	1.87E-09
11	2625242	0	C/T	0.014	0.67	0.11	1.87E-09
11	2625268	0	T/C	0.014	0.67	0.11	1.87E-09
11	2625323	0	G/T	0.014	0.67	0.11	1.87E-09
11	2625352	0	G/C	0.014	0.67	0.11	1.87E-09
11	2625364	0	T/C	0.014	0.67	0.11	1.87E-09
11	2625542	0	G/C	0.014	0.67	0.11	1.87E-09
11	2625543	0	G/T	0.014	0.67	0.11	1.87E-09
11	2625567	0	G/C	0.014	0.67	0.11	1.87E-09
11	2625647	0	A/G	0.014	0.67	0.11	1.87E-09
11	2625654	0	C/T	0.014	0.67	0.11	1.87E-09
11	2625716	0	A/C	0.014	0.67	0.11	1.87E-09
11	2625807	0	T/C	0.014	0.67	0.11	1.87E-09
11	2625854	0	A/G	0.014	0.67	0.11	1.87E-09
11	2625987	0	C/T	0.014	0.67	0.11	1.87E-09
11	2626069	0	T/C	0.014	0.67	0.11	1.87E-09
11	2626167	0	T/C	0.014	0.67	0.11	1.87E-09
11	2626318	0	C/A	0.014	0.67	0.11	1.87E-09
11	2626321	0	A/C	0.014	0.67	0.11	1.87E-09
11	2626334	0	A/G	0.014	0.67	0.11	1.87E-09
11	2626346	0	T/G	0.014	0.67	0.11	1.87E-09
11	2626428	0	G/A	0.014	0.67	0.11	1.87E-09
11	2626484	0	G/C	0.014	0.67	0.11	1.87E-09
11	2626578	0	T/G	0.014	0.67	0.11	1.87E-09
11	2626627	0	A/G	0.014	0.67	0.11	1.87E-09
11	2626735	0	G/A	0.014	0.67	0.11	1.87E-09
11	2627012	0	T/C	0.014	0.67	0.11	1.87E-09
11	2627013	0	G/A	0.014	0.67	0.11	1.87E-09

11	2627025	0	A/G	0.014	0.67	0.11	1.87E-09
11	2627049	0	G/T	0.014	0.67	0.11	1.87E-09
11	2627054	0	G/A	0.014	0.67	0.11	1.87E-09
11	2627168	0	G/A	0.014	0.67	0.11	1.87E-09
11	2627254	0	C/G	0.014	0.67	0.11	1.87E-09
11	2627280	0	G/A	0.014	0.67	0.11	1.87E-09
11	2627292	0	A/G	0.014	0.67	0.11	1.87E-09
11	2627342	0	T/C	0.014	0.67	0.11	1.87E-09
11	2627392	0	G/T	0.014	0.67	0.11	1.87E-09
11	2627484	0	G/A	0.014	0.67	0.11	1.87E-09
11	2627524	0	A/C	0.014	0.67	0.11	1.87E-09
11	2627540	0	T/G	0.014	0.67	0.11	1.87E-09
11	2627544	0	A/G	0.014	0.67	0.11	1.87E-09
11	2627563	0	G/A	0.014	0.67	0.11	1.87E-09
11	2627567	0	A/C	0.014	0.67	0.11	1.87E-09
11	2627568	0	C/T	0.014	0.67	0.11	1.87E-09
11	2627571	0	A/G	0.014	0.67	0.11	1.87E-09
11	2627576	0	T/C	0.014	0.67	0.11	1.87E-09
11	2627578	0	T/G	0.014	0.67	0.11	1.87E-09
11	2627598	0	A/C	0.014	0.67	0.11	1.87E-09
11	2627602	0	T/C	0.014	0.67	0.11	1.87E-09
11	2627658	0	G/T	0.014	0.67	0.11	1.87E-09
11	2627675	0	T/C	0.014	0.67	0.11	1.87E-09
11	2627687	0	G/C	0.014	0.67	0.11	1.87E-09
11	2627696	0	T/C	0.014	0.67	0.11	1.87E-09
11	2627697	0	T/C	0.014	0.67	0.11	1.87E-09
11	2627700	0	C/T	0.014	0.67	0.11	1.87E-09
11	2627718	0	C/A	0.014	0.67	0.11	1.87E-09
11	2627722	0	C/A	0.014	0.67	0.11	1.87E-09
11	2627730	0	A/G	0.014	0.67	0.11	1.87E-09
11	2627737	0	T/C	0.014	0.67	0.11	1.87E-09
11	2627775	0	T/C	0.014	0.67	0.11	1.87E-09
11	2627776	0	C/T	0.014	0.67	0.11	1.87E-09
11	2627817	0	C/T	0.014	0.67	0.11	1.87E-09
11	2627828	0	T/C	0.014	0.67	0.11	1.87E-09
11	2627866	0	T/G	0.014	0.67	0.11	1.87E-09
11	2627871	0	A/G	0.014	0.67	0.11	1.87E-09
11	2627877	0	A/T	0.014	0.67	0.11	1.87E-09
11	2627992	0	A/G	0.014	0.67	0.11	1.87E-09
11	2628006	0	G/T	0.014	0.67	0.11	1.87E-09

11	2628058	0	C/T	0.014	0.67	0.11	1.87E-09
11	2628106	0	A/T	0.014	0.67	0.11	1.87E-09
11	2628137	0	C/T	0.014	0.67	0.11	1.87E-09
11	2628206	0	G/A	0.014	0.67	0.11	1.87E-09
11	2628373	0	A/G	0.014	0.67	0.11	1.87E-09
11	2628378	0	T/C	0.014	0.67	0.11	1.87E-09
11	2628502	0	A/G	0.014	0.67	0.11	1.87E-09
11	2628537	0	C/A	0.014	0.67	0.11	1.87E-09
11	2628566	0	C/A	0.014	0.67	0.11	1.87E-09
11	2628632	0	A/G	0.014	0.67	0.11	1.87E-09
11	2628633	0	A/G	0.014	0.67	0.11	1.87E-09
11	2628638	0	A/C	0.014	0.67	0.11	1.87E-09
11	2628669	0	C/T	0.014	0.67	0.11	1.87E-09
11	2628712	0	A/C	0.014	0.67	0.11	1.87E-09
11	2628740	0	T/C	0.014	0.67	0.11	1.87E-09
11	2629149	0	A/T	0.014	0.67	0.11	1.87E-09
11	2629152	0	T/C	0.014	0.67	0.11	1.87E-09
11	2629601	0	C/A	0.014	0.67	0.11	1.87E-09
11	2629626	0	C/T	0.014	0.67	0.11	1.87E-09
11	2629628	0	C/T	0.014	0.67	0.11	1.87E-09
11	2629629	0	T/G	0.014	0.67	0.11	1.87E-09
11	2629666	0	C/T	0.014	0.67	0.11	1.87E-09
11	2629684	0	G/A	0.014	0.67	0.11	1.87E-09
11	2629759	0	A/G	0.014	0.67	0.11	1.87E-09
11	2629762	0	C/T	0.014	0.67	0.11	1.87E-09
11	2629781	0	A/G	0.014	0.67	0.11	1.87E-09
11	2629812	0	A/G	0.014	0.67	0.11	1.87E-09
11	2629868	0	C/G	0.014	0.67	0.11	1.87E-09
11	2629897	0	C/T	0.014	0.67	0.11	1.87E-09
11	2629915	0	G/T	0.014	0.67	0.11	1.87E-09
11	2629925	0	A/G	0.014	0.67	0.11	1.87E-09
11	2629977	0	A/G	0.014	0.67	0.11	1.87E-09
11	2629978	0	C/A	0.014	0.67	0.11	1.87E-09
11	2629989	0	G/A	0.014	0.67	0.11	1.87E-09
11	2630057	0	C/G	0.014	0.67	0.11	1.87E-09
11	2630079	0	G/A	0.014	0.67	0.11	1.87E-09
11	2630116	0	A/T	0.014	0.67	0.11	1.87E-09
11	2630125	0	A/G	0.014	0.67	0.11	1.87E-09
11	2630369	0	A/G	0.014	0.67	0.11	1.87E-09
11	2630608	0	G/A	0.014	0.67	0.11	1.87E-09

11	2630630	0	T/C	0.014	0.67	0.11	1.87E-09
11	2630636	0	C/T	0.014	0.67	0.11	1.87E-09
11	2630651	0	G/A	0.014	0.67	0.11	1.87E-09
11	2630681	0	G/T	0.014	0.67	0.11	1.87E-09
11	2630688	0	A/T	0.014	0.67	0.11	1.87E-09
11	2630689	0	A/T	0.014	0.67	0.11	1.87E-09
11	2630774	0	T/C	0.014	0.67	0.11	1.87E-09
11	2630779	0	T/C	0.014	0.67	0.11	1.87E-09
11	2630947	0	C/T	0.014	0.67	0.11	1.87E-09
11	2631030	0	T/C	0.014	0.67	0.11	1.87E-09
11	2631079	0	T/C	0.014	0.67	0.11	1.87E-09
11	2631283	0	G/A	0.014	0.67	0.11	1.87E-09
11	2631339	0	A/G	0.014	0.67	0.11	1.87E-09
11	2631429	0	A/C	0.014	0.67	0.11	1.87E-09
11	2631687	0	A/G	0.014	0.67	0.11	1.87E-09
11	2632256	0	T/A	0.014	0.67	0.11	1.87E-09
11	2632259	0	A/G	0.014	0.67	0.11	1.87E-09
11	2632609	0	T/C	0.014	0.67	0.11	1.87E-09
11	2632707	0	A/G	0.014	0.67	0.11	1.87E-09
11	2632861	0	A/G	0.014	0.67	0.11	1.87E-09
11	2633196	0	T/C	0.014	0.67	0.11	1.87E-09
11	2633651	0	G/A	0.014	0.67	0.11	1.87E-09
11	2633854	0	T/C	0.014	0.67	0.11	1.87E-09
11	2633942	0	G/A	0.014	0.67	0.11	1.87E-09
11	2634145	0	A/G	0.014	0.67	0.11	1.87E-09
11	2634215	0	C/A	0.014	0.67	0.11	1.87E-09
11	2634216	0	A/G	0.014	0.67	0.11	1.87E-09
11	2634271	0	G/A	0.014	0.67	0.11	1.87E-09
11	2634282	0	C/T	0.014	0.67	0.11	1.87E-09
11	2634374	0	C/G	0.014	0.67	0.11	1.87E-09
11	2634444	0	T/A	0.014	0.67	0.11	1.87E-09
11	2634482	0	T/C	0.014	0.67	0.11	1.87E-09
11	2634578	0	C/A	0.014	0.67	0.11	1.87E-09
11	2634702	0	G/A	0.014	0.67	0.11	1.87E-09
11	2634746	0	G/A	0.014	0.67	0.11	1.87E-09
11	2634837	0	C/T	0.014	0.67	0.11	1.87E-09
11	2634867	0	T/C	0.014	0.67	0.11	1.87E-09
11	2634891	0	A/G	0.014	0.67	0.11	1.87E-09
11	2635020	0	T/C	0.014	0.67	0.11	1.87E-09
11	2635045	0	C/G	0.014	0.67	0.11	1.87E-09

11	2635340	0	C/T	0.014	0.67	0.11	1.87E-09
11	2635420	0	T/C	0.014	0.67	0.11	1.87E-09
11	2635617	0	A/G	0.014	0.67	0.11	1.87E-09
11	2635877	0	A/G	0.014	0.67	0.11	1.87E-09
11	2635881	0	T/C	0.014	0.67	0.11	1.87E-09
11	2635900	0	T/C	0.014	0.67	0.11	1.87E-09
11	2635954	0	G/A	0.014	0.67	0.11	1.87E-09
11	2635956	0	A/T	0.014	0.67	0.11	1.87E-09
11	2635983	0	C/T	0.014	0.67	0.11	1.87E-09
11	2635990	0	A/G	0.014	0.67	0.11	1.87E-09
11	2636017	0	C/A	0.014	0.67	0.11	1.87E-09
11	2636030	0	T/C	0.014	0.67	0.11	1.87E-09
11	2636107	0	G/C	0.014	0.67	0.11	1.87E-09
11	2636198	0	C/T	0.014	0.67	0.11	1.87E-09
11	2636226	0	C/T	0.014	0.67	0.11	1.87E-09
11	2636265	0	C/T	0.014	0.67	0.11	1.87E-09
11	2636331	0	A/C	0.014	0.67	0.11	1.87E-09
11	2636823	0	T/C	0.014	0.67	0.11	1.87E-09
11	2637354	0	C/T	0.014	0.67	0.11	1.87E-09
11	2638010	0	A/G	0.014	0.67	0.11	1.87E-09
11	2638453	0	G/A	0.014	0.67	0.11	1.87E-09
11	2638473	0	G/A	0.014	0.67	0.11	1.87E-09
11	2638475	0	G/A	0.014	0.67	0.11	1.87E-09
11	2638482	0	A/C	0.014	0.67	0.11	1.87E-09
11	2638997	0	T/G	0.014	0.67	0.11	1.87E-09
11	2639016	0	T/G	0.014	0.67	0.11	1.87E-09
11	2639070	0	A/G	0.014	0.67	0.11	1.87E-09
11	2639166	0	T/G	0.014	0.67	0.11	1.87E-09
11	2639277	0	G/A	0.014	0.67	0.11	1.87E-09
11	2639282	0	T/C	0.014	0.67	0.11	1.87E-09
11	2639332	0	G/A	0.014	0.67	0.11	1.87E-09
11	2639343	0	A/G	0.014	0.67	0.11	1.87E-09
11	2639372	0	C/T	0.014	0.67	0.11	1.87E-09
11	2639373	0	A/G	0.014	0.67	0.11	1.87E-09
11	2639402	0	A/G	0.014	0.67	0.11	1.87E-09
11	2639404	0	T/C	0.014	0.67	0.11	1.87E-09
11	2639498	0	T/A	0.014	0.67	0.11	1.87E-09
11	2639500	0	T/C	0.014	0.67	0.11	1.87E-09
11	2639531	0	C/T	0.014	0.67	0.11	1.87E-09
11	2639546	0	C/T	0.014	0.67	0.11	1.87E-09

11	2639643	0	G/A	0.014	0.67	0.11	1.87E-09
11	2639647	0	G/T	0.014	0.67	0.11	1.87E-09
11	2640401	0	G/A	0.014	0.67	0.11	1.87E-09
11	2640946	0	T/C	0.014	0.67	0.11	1.87E-09
11	2641350	0	A/G	0.014	0.67	0.11	1.87E-09
11	2641510	0	A/G	0.014	0.67	0.11	1.87E-09
11	2641577	0	A/C	0.014	0.67	0.11	1.87E-09
11	2641578	0	C/T	0.014	0.67	0.11	1.87E-09
11	2641950	0	C/G	0.014	0.67	0.11	1.87E-09
11	2641959	0	G/A	0.014	0.67	0.11	1.87E-09
11	2642060	0	T/C	0.014	0.67	0.11	1.87E-09
11	2642122	0	T/C	0.014	0.67	0.11	1.87E-09
11	2642631	0	T/C	0.014	0.67	0.11	1.87E-09
11	2642891	0	A/G	0.014	0.67	0.11	1.87E-09
11	2643466	0	T/C	0.014	0.67	0.11	1.87E-09
11	2643855	0	T/C	0.014	0.67	0.11	1.87E-09
11	2644394	0	T/C	0.014	0.67	0.11	1.87E-09
11	2644430	0	T/G	0.014	0.67	0.11	1.87E-09
11	2646400	0	T/C	0.014	0.67	0.11	1.87E-09
11	2646668	0	T/G	0.014	0.67	0.11	1.87E-09
11	2647440	0	C/T	0.014	0.67	0.11	1.87E-09
11	2648047	0	T/C	0.014	0.67	0.11	1.87E-09
11	2648134	0	G/A	0.014	0.67	0.11	1.87E-09
11	2648679	0	T/A	0.014	0.67	0.11	1.87E-09
11	2648745	0	A/G	0.014	0.67	0.11	1.87E-09
11	2648787	0	A/C	0.014	0.67	0.11	1.87E-09
11	2648857	0	A/G	0.014	0.67	0.11	1.87E-09
11	2648866	0	T/C	0.014	0.67	0.11	1.87E-09
11	2649642	0	T/C	0.014	0.67	0.11	1.87E-09
11	2652472	0	G/A	0.014	0.67	0.11	1.87E-09
11	2658815	0	C/G	0.014	0.67	0.11	1.87E-09
11	2658826	0	G/A	0.014	0.67	0.11	1.87E-09
11	2667081	0	A/C	0.014	0.67	0.11	1.87E-09
11	2667313	0	C/A	0.014	0.67	0.11	1.87E-09
11	2667843	0	C/G	0.014	0.67	0.11	1.87E-09
11	2668618	0	A/C	0.014	0.67	0.11	1.87E-09
11	2669931	0	T/C	0.014	0.67	0.11	1.87E-09
11	2670253	0	T/C	0.014	0.67	0.11	1.87E-09
11	2671546	0	T/C	0.014	0.67	0.11	1.87E-09
11	2673036	0	C/A	0.014	0.67	0.11	1.87E-09

11	2673465	0	C/G	0.014	0.67	0.11	1.87E-09
11	2674014	0	T/C	0.014	0.67	0.11	1.87E-09
11	2674261	0	A/G	0.014	0.67	0.11	1.87E-09
11	2674263	0	T/G	0.014	0.67	0.11	1.87E-09
11	2675500	0	C/G	0.014	0.67	0.11	1.87E-09
11	2675583	0	A/G	0.014	0.67	0.11	1.87E-09
11	2678211	0	A/G	0.014	0.67	0.11	1.87E-09
11	2678525	0	G/A	0.014	0.67	0.11	1.87E-09
11	2678847	0	G/T	0.014	0.67	0.11	1.87E-09
11	2678855	0	A/G	0.014	0.67	0.11	1.87E-09
11	2678866	0	T/C	0.014	0.67	0.11	1.87E-09
11	2678871	0	C/T	0.014	0.67	0.11	1.87E-09
11	2678928	0	C/T	0.014	0.67	0.11	1.87E-09
11	2678946	0	G/A	0.014	0.67	0.11	1.87E-09
11	2679138	0	T/C	0.014	0.67	0.11	1.87E-09
11	2679179	0	A/G	0.014	0.67	0.11	1.87E-09
11	2679340	0	C/G	0.014	0.67	0.11	1.87E-09
11	2679492	0	A/G	0.014	0.67	0.11	1.87E-09
11	2679957	0	T/C	0.014	0.67	0.11	1.87E-09
11	2680576	0	A/G	0.014	0.67	0.11	1.87E-09
11	2680924	0	C/T	0.014	0.67	0.11	1.87E-09
11	2680926	0	A/G	0.014	0.67	0.11	1.87E-09
11	2680927	0	C/G	0.014	0.67	0.11	1.87E-09
11	2680953	0	C/T	0.014	0.67	0.11	1.87E-09
11	2681031	0	T/C	0.014	0.67	0.11	1.87E-09
11	2681069	0	G/A	0.014	0.67	0.11	1.87E-09
11	2681307	0	C/G	0.014	0.67	0.11	1.87E-09
11	2681900	0	C/T	0.014	0.67	0.11	1.87E-09
11	2682365	0	T/C	0.014	0.67	0.11	1.87E-09
11	2683040	0	T/C	0.014	0.67	0.11	1.87E-09
11	2683066	0	T/A	0.014	0.67	0.11	1.87E-09
11	2683071	0	C/A	0.014	0.67	0.11	1.87E-09
11	2683767	0	T/A	0.014	0.67	0.11	1.87E-09
11	2683942	0	G/A	0.014	0.67	0.11	1.87E-09
11	2683962	0	T/G	0.014	0.67	0.11	1.87E-09
11	2684057	0	C/T	0.014	0.67	0.11	1.87E-09
11	2686345	0	T/C	0.014	0.67	0.11	1.87E-09
11	2686404	0	A/C	0.014	0.67	0.11	1.87E-09
11	2686928	0	A/G	0.014	0.67	0.11	1.87E-09
11	2687162	0	A/G	0.014	0.67	0.11	1.87E-09

11	2687363	0	A/G	0.014	0.67	0.11	1.87E-09
11	2687984	0	A/G	0.014	0.67	0.11	1.87E-09
11	2688240	0	A/C	0.014	0.67	0.11	1.87E-09
11	2688471	0	G/A	0.014	0.67	0.11	1.87E-09
11	2688548	0	G/T	0.014	0.67	0.11	1.87E-09
11	2688626	0	T/C	0.014	0.67	0.11	1.87E-09
11	2688630	0	G/A	0.014	0.67	0.11	1.87E-09
11	2688890	0	C/G	0.014	0.67	0.11	1.87E-09
11	2688914	0	G/A	0.014	0.67	0.11	1.87E-09
11	2689120	0	G/C	0.014	0.67	0.11	1.87E-09
11	2689132	0	C/T	0.014	0.67	0.11	1.87E-09
11	2689339	0	G/C	0.014	0.67	0.11	1.87E-09
11	2689346	0	C/T	0.014	0.67	0.11	1.87E-09
11	2689846	0	A/G	0.014	0.67	0.11	1.87E-09
11	2689901	0	G/C	0.014	0.67	0.11	1.87E-09
11	2690025	0	T/C	0.014	0.67	0.11	1.87E-09
11	2690298	0	C/T	0.014	0.67	0.11	1.87E-09
11	2690306	0	G/A	0.014	0.67	0.11	1.87E-09
11	2690311	0	G/A	0.014	0.67	0.11	1.87E-09
11	2690421	0	C/T	0.014	0.67	0.11	1.87E-09
11	2690564	0	G/C	0.014	0.67	0.11	1.87E-09
11	2690647	0	A/C	0.014	0.67	0.11	1.87E-09
11	2690661	0	T/A	0.014	0.67	0.11	1.87E-09
11	2690665	0	G/A	0.014	0.67	0.11	1.87E-09
11	2690670	0	G/A	0.014	0.67	0.11	1.87E-09
11	2690677	0	A/C	0.014	0.67	0.11	1.87E-09
11	2690687	0	T/C	0.014	0.67	0.11	1.87E-09
11	2690797	0	G/A	0.014	0.67	0.11	1.87E-09
11	2691454	0	A/G	0.014	0.67	0.11	1.87E-09
11	2691654	0	G/A	0.014	0.67	0.11	1.87E-09
11	2691655	0	A/G	0.014	0.67	0.11	1.87E-09
11	2693007	0	T/A	0.014	0.67	0.11	1.87E-09
11	2693400	0	T/C	0.014	0.67	0.11	1.87E-09
11	2693735	0	G/A	0.014	0.67	0.11	1.87E-09
11	2693844	0	A/G	0.014	0.67	0.11	1.87E-09
11	2694123	0	T/A	0.014	0.67	0.11	1.87E-09
11	2694438	0	A/G	0.014	0.67	0.11	1.87E-09
11	2696976	0	A/G	0.014	0.67	0.11	1.87E-09
11	2697511	0	T/C	0.014	0.67	0.11	1.87E-09
11	2697718	0	A/G	0.014	0.67	0.11	1.87E-09

11	2697854	0	T/C	0.014	0.67	0.11	1.87E-09
11	2698613	0	G/A	0.014	0.67	0.11	1.87E-09
11	2698721	0	C/G	0.014	0.67	0.11	1.87E-09
11	2698901	0	G/C	0.014	0.67	0.11	1.87E-09
11	2699028	0	G/A	0.014	0.67	0.11	1.87E-09
11	2699398	0	A/G	0.014	0.67	0.11	1.87E-09
11	2699542	0	A/G	0.014	0.67	0.11	1.87E-09
11	2699654	0	A/G	0.014	0.67	0.11	1.87E-09
11	2699873	0	G/A	0.014	0.67	0.11	1.87E-09
11	2699961	0	A/C	0.014	0.67	0.11	1.87E-09
11	2700167	0	T/C	0.014	0.67	0.11	1.87E-09
11	2700678	0	A/T	0.014	0.67	0.11	1.87E-09
11	2700681	0	T/C	0.014	0.67	0.11	1.87E-09
11	2700791	0	A/G	0.014	0.67	0.11	1.87E-09
11	2700792	0	G/C	0.014	0.67	0.11	1.87E-09
11	2700833	0	A/G	0.014	0.67	0.11	1.87E-09
11	2701213	0	G/A	0.014	0.67	0.11	1.87E-09
11	2701242	0	A/G	0.014	0.67	0.11	1.87E-09
11	2701251	0	C/T	0.014	0.67	0.11	1.87E-09
11	2701319	0	T/G	0.014	0.67	0.11	1.87E-09
11	2701354	0	C/G	0.014	0.67	0.11	1.87E-09
11	2701540	0	A/G	0.014	0.67	0.11	1.87E-09
11	2701704	0	C/T	0.014	0.67	0.11	1.87E-09
11	2701726	0	C/T	0.014	0.67	0.11	1.87E-09
11	2702040	0	T/C	0.014	0.67	0.11	1.87E-09
11	2702115	0	T/C	0.014	0.67	0.11	1.87E-09
11	2702227	0	T/C	0.014	0.67	0.11	1.87E-09
11	2702299	0	T/C	0.014	0.67	0.11	1.87E-09
11	2702317	0	T/G	0.014	0.67	0.11	1.87E-09
11	2702340	0	A/G	0.014	0.67	0.11	1.87E-09
11	2702724	0	C/G	0.014	0.67	0.11	1.87E-09
11	2702807	0	G/T	0.014	0.67	0.11	1.87E-09
11	2702840	0	T/C	0.014	0.67	0.11	1.87E-09
11	2702878	0	C/T	0.014	0.67	0.11	1.87E-09
11	2702895	0	A/C	0.014	0.67	0.11	1.87E-09
11	2702988	0	C/T	0.014	0.67	0.11	1.87E-09
11	2703142	0	T/C	0.014	0.67	0.11	1.87E-09
11	2703171	0	A/G	0.014	0.67	0.11	1.87E-09
11	2703243	0	A/G	0.014	0.67	0.11	1.87E-09
11	2703283	0	T/C	0.014	0.67	0.11	1.87E-09

11	2703379	0	G/A	0.014	0.67	0.11	1.87E-09
11	2703420	0	A/G	0.014	0.67	0.11	1.87E-09
11	2703423	0	T/C	0.014	0.67	0.11	1.87E-09
11	2703429	0	C/T	0.014	0.67	0.11	1.87E-09
11	2703615	0	C/G	0.014	0.67	0.11	1.87E-09
11	2704487	0	C/A	0.014	0.67	0.11	1.87E-09
11	2704556	0	A/G	0.014	0.67	0.11	1.87E-09
11	2704584	0	G/A	0.014	0.67	0.11	1.87E-09
11	2704594	0	C/G	0.014	0.67	0.11	1.87E-09
11	2704678	0	A/C	0.014	0.67	0.11	1.87E-09
11	2704805	0	T/A	0.014	0.67	0.11	1.87E-09
11	2704856	0	G/A	0.014	0.67	0.11	1.87E-09
11	2704859	0	C/G	0.014	0.67	0.11	1.87E-09
11	2705407	0	G/A	0.014	0.67	0.11	1.87E-09
11	2705872	0	T/G	0.014	0.67	0.11	1.87E-09
11	2705954	0	T/C	0.014	0.67	0.11	1.87E-09
11	2705962	0	C/G	0.014	0.67	0.11	1.87E-09
11	2705973	0	A/G	0.014	0.67	0.11	1.87E-09
11	2705994	0	C/A	0.014	0.67	0.11	1.87E-09
11	2706064	0	A/G	0.014	0.67	0.11	1.87E-09
11	2706078	0	G/A	0.014	0.67	0.11	1.87E-09
11	2706103	0	T/G	0.014	0.67	0.11	1.87E-09
11	2706144	0	A/G	0.014	0.67	0.11	1.87E-09
11	2706167	0	T/A	0.014	0.67	0.11	1.87E-09
11	2706168	0	C/T	0.014	0.67	0.11	1.87E-09
11	2706268	0	T/G	0.014	0.67	0.11	1.87E-09
11	2706395	0	A/C	0.014	0.67	0.11	1.87E-09
11	2707007	0	G/T	0.014	0.67	0.11	1.87E-09
11	2707081	0	C/T	0.014	0.67	0.11	1.87E-09
11	2707337	0	T/C	0.014	0.67	0.11	1.87E-09
11	2707434	0	G/C	0.014	0.67	0.11	1.87E-09
11	2707691	0	A/G	0.014	0.67	0.11	1.87E-09
11	2707919	0	A/G	0.014	0.67	0.11	1.87E-09
11	2708663	0	T/C	0.014	0.67	0.11	1.87E-09
11	2709363	0	T/C	0.014	0.67	0.11	1.87E-09
11	2709383	0	A/G	0.014	0.67	0.11	1.87E-09
11	2709386	0	A/G	0.014	0.67	0.11	1.87E-09
11	2709401	0	T/C	0.014	0.67	0.11	1.87E-09
11	2709633	0	G/C	0.014	0.67	0.11	1.87E-09
11	2709669	0	T/G	0.014	0.67	0.11	1.87E-09

11	2709740	0	A/G	0.014	0.67	0.11	1.87E-09
11	2709787	0	A/G	0.014	0.67	0.11	1.87E-09
11	2710109	0	A/C	0.014	0.67	0.11	1.87E-09
11	2710124	0	C/T	0.014	0.67	0.11	1.87E-09
11	2710363	0	T/C	0.014	0.67	0.11	1.87E-09
11	2710418	0	C/T	0.014	0.67	0.11	1.87E-09
11	2710440	0	T/C	0.014	0.67	0.11	1.87E-09
11	2710457	0	C/T	0.014	0.67	0.11	1.87E-09
11	2710670	0	T/C	0.014	0.67	0.11	1.87E-09
11	2710699	0	A/G	0.014	0.67	0.11	1.87E-09
11	2710708	0	A/G	0.014	0.67	0.11	1.87E-09
11	2710710	0	G/A	0.014	0.67	0.11	1.87E-09
11	2710742	0	A/G	0.014	0.67	0.11	1.87E-09
11	2710780	0	A/G	0.014	0.67	0.11	1.87E-09
11	2710842	0	A/G	0.014	0.67	0.11	1.87E-09
11	2710865	0	A/G	0.014	0.67	0.11	1.87E-09
11	2711049	0	A/C	0.014	0.67	0.11	1.87E-09
11	2711051	0	A/C	0.014	0.67	0.11	1.87E-09
11	2711188	0	A/T	0.014	0.67	0.11	1.87E-09
11	2711645	0	A/T	0.014	0.67	0.11	1.87E-09
11	2711648	0	C/G	0.014	0.67	0.11	1.87E-09
11	2711690	0	T/C	0.014	0.67	0.11	1.87E-09
11	2713391	0	A/G	0.014	0.67	0.11	1.87E-09
11	2713497	0	C/T	0.014	0.67	0.11	1.87E-09
11	2713818	0	A/C	0.014	0.67	0.11	1.87E-09
11	2713992	0	G/A	0.014	0.67	0.11	1.87E-09
11	2714478	0	T/G	0.014	0.67	0.11	1.87E-09
11	2714493	0	A/C	0.014	0.67	0.11	1.87E-09
11	2714760	0	A/C	0.014	0.67	0.11	1.87E-09
11	2715116	0	T/C	0.014	0.67	0.11	1.87E-09
11	2715208	0	T/G	0.014	0.67	0.11	1.87E-09
11	2715708	0	A/G	0.014	0.67	0.11	1.87E-09
11	2716177	0	A/G	0.014	0.67	0.11	1.87E-09
11	2716694	0	G/A	0.014	0.67	0.11	1.87E-09
11	2716697	0	A/G	0.014	0.67	0.11	1.87E-09
11	2716803	0	A/G	0.014	0.67	0.11	1.87E-09
11	2717554	0	C/G	0.014	0.67	0.11	1.87E-09
11	2718303	0	C/A	0.014	0.67	0.11	1.87E-09
11	2718433	0	C/T	0.014	0.67	0.11	1.87E-09
11	2718486	0	T/C	0.014	0.67	0.11	1.87E-09

11	2718497	0	G/A	0.014	0.67	0.11	1.87E-09
11	2718564	0	T/C	0.014	0.67	0.11	1.87E-09
11	2718597	0	A/G	0.014	0.67	0.11	1.87E-09
11	2718737	0	A/C	0.014	0.67	0.11	1.87E-09
11	2718748	0	T/C	0.014	0.67	0.11	1.87E-09
11	2718757	0	G/A	0.014	0.67	0.11	1.87E-09
11	2719151	0	G/A	0.014	0.67	0.11	1.87E-09
11	2719157	0	C/T	0.014	0.67	0.11	1.87E-09
11	2719229	0	G/A	0.014	0.67	0.11	1.87E-09
11	2719344	0	G/T	0.014	0.67	0.11	1.87E-09
11	2719361	0	C/G	0.014	0.67	0.11	1.87E-09
11	2719430	0	T/C	0.014	0.67	0.11	1.87E-09
11	2719453	0	G/A	0.014	0.67	0.11	1.87E-09
11	2719480	0	A/C	0.014	0.67	0.11	1.87E-09
11	2719497	0	A/G	0.014	0.67	0.11	1.87E-09
11	2719609	0	C/T	0.014	0.67	0.11	1.87E-09
11	2719610	0	A/G	0.014	0.67	0.11	1.87E-09
11	2719697	0	A/G	0.014	0.67	0.11	1.87E-09
11	2719702	0	G/C	0.014	0.67	0.11	1.87E-09
11	2719710	0	T/C	0.014	0.67	0.11	1.87E-09
11	2719803	0	T/C	0.014	0.67	0.11	1.87E-09
11	2744988	0	A/G	0.014	0.67	0.11	1.87E-09
11	2745006	0	C/T	0.014	0.67	0.11	1.87E-09
11	2745053	0	G/T	0.014	0.67	0.11	1.87E-09
11	2745071	0	G/A	0.014	0.67	0.11	1.87E-09
11	2745773	0	G/A	0.014	0.67	0.11	1.87E-09
11	2746369	0	A/C	0.014	0.67	0.11	1.87E-09
11	2746671	0	G/A	0.014	0.67	0.11	1.87E-09
11	2747012	0	A/G	0.014	0.67	0.11	1.87E-09
11	2747025	0	C/T	0.014	0.67	0.11	1.87E-09
11	2747128	0	A/G	0.014	0.67	0.11	1.87E-09
11	2747169	0	A/G	0.014	0.67	0.11	1.87E-09
11	2747202	0	G/A	0.014	0.67	0.11	1.87E-09
11	2747723	0	T/C	0.014	0.67	0.11	1.87E-09
11	2747762	0	C/A	0.014	0.67	0.11	1.87E-09
11	2747923	0	A/G	0.014	0.67	0.11	1.87E-09
11	2748077	0	C/A	0.014	0.67	0.11	1.87E-09
11	2748118	0	T/G	0.014	0.67	0.11	1.87E-09
11	2748147	0	T/C	0.014	0.67	0.11	1.87E-09
11	2748152	0	A/T	0.014	0.67	0.11	1.87E-09

11	2748166	0	G/C	0.014	0.67	0.11	1.87E-09
11	2748260	0	C/T	0.014	0.67	0.11	1.87E-09
11	2748396	0	T/G	0.014	0.67	0.11	1.87E-09
11	2749203	0	G/A	0.014	0.67	0.11	1.87E-09
11	2749795	0	A/G	0.014	0.67	0.11	1.87E-09
11	2749971	0	G/T	0.014	0.67	0.11	1.87E-09
11	2749976	0	A/G	0.014	0.67	0.11	1.87E-09
11	2750359	0	C/T	0.014	0.67	0.11	1.87E-09
11	2751202	0	G/A	0.014	0.67	0.11	1.87E-09
11	2751420	0	A/G	0.014	0.67	0.11	1.87E-09
11	2751428	0	A/G	0.014	0.67	0.11	1.87E-09
11	2751435	0	C/A	0.014	0.67	0.11	1.87E-09
11	2751511	0	A/G	0.014	0.67	0.11	1.87E-09
11	2751515	0	C/A	0.014	0.67	0.11	1.87E-09
11	2751522	0	C/A	0.014	0.67	0.11	1.87E-09
11	2751802	0	T/C	0.014	0.67	0.11	1.87E-09
11	2751837	0	G/A	0.014	0.67	0.11	1.87E-09
11	2767477	0	G/A	0.014	0.67	0.11	1.87E-09
11	2768746	0	G/A	0.014	0.67	0.11	1.87E-09
11	2768962	0	T/C	0.014	0.67	0.11	1.87E-09
11	2768975	0	T/C	0.014	0.67	0.11	1.87E-09
11	2769034	0	G/T	0.014	0.67	0.11	1.87E-09
11	2769039	0	T/G	0.014	0.67	0.11	1.87E-09
11	2769052	0	G/A	0.014	0.67	0.11	1.87E-09
11	2769129	0	G/A	0.014	0.67	0.11	1.87E-09
11	2769134	0	A/G	0.014	0.67	0.11	1.87E-09
11	2769135	0	T/A	0.014	0.67	0.11	1.87E-09
11	2769141	0	C/T	0.014	0.67	0.11	1.87E-09
11	2769153	0	C/T	0.014	0.67	0.11	1.87E-09
11	2769160	0	C/G	0.014	0.67	0.11	1.87E-09
11	2769162	0	A/G	0.014	0.67	0.11	1.87E-09
11	2769167	0	G/A	0.014	0.67	0.11	1.87E-09
11	2769203	0	A/G	0.014	0.67	0.11	1.87E-09
11	2769242	0	T/C	0.014	0.67	0.11	1.87E-09
11	2769250	0	G/C	0.014	0.67	0.11	1.87E-09
11	2769252	0	T/C	0.014	0.67	0.11	1.87E-09
11	2769256	0	A/G	0.014	0.67	0.11	1.87E-09
11	2769287	0	G/C	0.014	0.67	0.11	1.87E-09
11	2769374	0	T/G	0.014	0.67	0.11	1.87E-09
11	2769440	0	C/T	0.014	0.67	0.11	1.87E-09

11	2769441	0	C/T	0.014	0.67	0.11	1.87E-09
11	2769518	0	G/A	0.014	0.67	0.11	1.87E-09
11	2769674	0	C/T	0.014	0.67	0.11	1.87E-09
11	2769683	0	G/A	0.014	0.67	0.11	1.87E-09
11	2769792	0	A/G	0.014	0.67	0.11	1.87E-09
11	2770030	0	C/A	0.014	0.67	0.11	1.87E-09
11	2770038	0	A/G	0.014	0.67	0.11	1.87E-09
11	2770207	0	T/G	0.014	0.67	0.11	1.87E-09
11	2770416	0	T/C	0.014	0.67	0.11	1.87E-09
11	2771270	0	A/G	0.014	0.67	0.11	1.87E-09
11	2775748	0	G/A	0.014	0.67	0.11	1.87E-09
11	2775973	0	A/G	0.014	0.67	0.11	1.87E-09
11	2785612	0	C/G	0.014	0.67	0.11	1.87E-09
11	2786568	0	A/C	0.014	0.67	0.11	1.87E-09
11	2786921	0	A/G	0.014	0.67	0.11	1.87E-09
11	2787851	0	T/G	0.014	0.67	0.11	1.87E-09
11	2787856	0	T/C	0.014	0.67	0.11	1.87E-09
11	2788416	0	C/A	0.014	0.67	0.11	1.87E-09
11	2788657	0	T/C	0.014	0.67	0.11	1.87E-09
11	2788960	0	G/A	0.014	0.67	0.11	1.87E-09
11	2789301	0	T/G	0.014	0.67	0.11	1.87E-09
11	2789344	0	G/A	0.014	0.67	0.11	1.87E-09
11	2790753	0	T/C	0.014	0.67	0.11	1.87E-09
11	2793269	0	T/A	0.014	0.67	0.11	1.87E-09
11	2793451	0	T/C	0.014	0.67	0.11	1.87E-09
11	2793704	0	G/C	0.014	0.67	0.11	1.87E-09
11	2793871	0	T/C	0.014	0.67	0.11	1.87E-09
11	2794186	0	A/G	0.014	0.67	0.11	1.87E-09
11	2794212	0	C/G	0.014	0.67	0.11	1.87E-09
11	2794983	0	G/A	0.014	0.67	0.11	1.87E-09
11	2794998	0	A/G	0.014	0.67	0.11	1.87E-09
11	2795029	0	A/G	0.014	0.67	0.11	1.87E-09
11	2795380	0	A/G	0.014	0.67	0.11	1.87E-09
11	2796052	0	G/C	0.014	0.67	0.11	1.87E-09
11	2796339	0	A/G	0.014	0.67	0.11	1.87E-09
11	2797042	0	G/A	0.014	0.67	0.11	1.87E-09
11	2797193	0	T/G	0.014	0.67	0.11	1.87E-09
11	2797223	0	T/G	0.014	0.67	0.11	1.87E-09
11	2797296	0	T/C	0.014	0.67	0.11	1.87E-09
11	2797297	0	G/C	0.014	0.67	0.11	1.87E-09

11	2797523	0	A/G	0.014	0.67	0.11	1.87E-09
11	2797782	0	C/T	0.014	0.67	0.11	1.87E-09
11	2797888	0	T/G	0.014	0.67	0.11	1.87E-09
11	2798080	0	C/T	0.014	0.67	0.11	1.87E-09
11	2798192	0	G/C	0.014	0.67	0.11	1.87E-09
11	2798262	0	A/G	0.014	0.67	0.11	1.87E-09
11	2798313	0	T/C	0.014	0.67	0.11	1.87E-09
11	2798372	0	C/G	0.014	0.67	0.11	1.87E-09
11	2798382	0	T/C	0.014	0.67	0.11	1.87E-09
11	2798383	0	C/G	0.014	0.67	0.11	1.87E-09
11	2798405	0	T/C	0.014	0.67	0.11	1.87E-09
11	2798429	0	C/G	0.014	0.67	0.11	1.87E-09
11	2798466	0	G/A	0.014	0.67	0.11	1.87E-09
11	2798476	0	T/C	0.014	0.67	0.11	1.87E-09
11	2798492	0	T/C	0.014	0.67	0.11	1.87E-09
11	2798533	0	G/A	0.014	0.67	0.11	1.87E-09
11	2798548	0	G/A	0.014	0.67	0.11	1.87E-09
11	2798551	0	A/G	0.014	0.67	0.11	1.87E-09
11	2798572	0	A/G	0.014	0.67	0.11	1.87E-09
11	2798704	0	G/A	0.014	0.67	0.11	1.87E-09
11	2798775	0	C/A	0.014	0.67	0.11	1.87E-09
11	2798890	0	G/A	0.014	0.67	0.11	1.87E-09
11	2799048	0	T/C	0.014	0.67	0.11	1.87E-09
11	2799173	0	C/T	0.014	0.67	0.11	1.87E-09
11	2799181	0	T/G	0.014	0.67	0.11	1.87E-09
11	2799226	0	T/C	0.014	0.67	0.11	1.87E-09
11	2799257	0	C/T	0.014	0.67	0.11	1.87E-09
11	2799303	0	G/A	0.014	0.67	0.11	1.87E-09
11	2799634	0	A/G	0.014	0.67	0.11	1.87E-09
11	2800044	0	A/G	0.014	0.67	0.11	1.87E-09
11	2800176	0	T/C	0.014	0.67	0.11	1.87E-09
11	2800401	0	T/C	0.014	0.67	0.11	1.87E-09
11	2800501	0	C/T	0.014	0.67	0.11	1.87E-09
11	2800516	0	C/G	0.014	0.67	0.11	1.87E-09
11	2800597	0	T/C	0.014	0.67	0.11	1.87E-09
11	2800735	0	C/T	0.014	0.67	0.11	1.87E-09
11	2800843	0	A/G	0.014	0.67	0.11	1.87E-09
11	2801172	0	A/G	0.014	0.67	0.11	1.87E-09
11	2801368	0	T/C	0.014	0.67	0.11	1.87E-09
11	2801579	0	T/C	0.014	0.67	0.11	1.87E-09

11	2801687	0	C/G	0.014	0.67	0.11	1.87E-09
11	2801962	0	T/G	0.014	0.67	0.11	1.87E-09
11	2802096	0	G/C	0.014	0.67	0.11	1.87E-09
11	2802186	0	G/C	0.014	0.67	0.11	1.87E-09
11	2802238	0	T/C	0.014	0.67	0.11	1.87E-09
11	2802260	0	T/C	0.014	0.67	0.11	1.87E-09
11	2802276	0	G/A	0.014	0.67	0.11	1.87E-09
11	2802298	0	T/G	0.014	0.67	0.11	1.87E-09
11	2802299	0	A/C	0.014	0.67	0.11	1.87E-09
11	2802330	0	A/G	0.014	0.67	0.11	1.87E-09
11	2802552	0	A/G	0.014	0.67	0.11	1.87E-09
11	2802849	0	G/A	0.014	0.67	0.11	1.87E-09
11	2802880	0	G/C	0.014	0.67	0.11	1.87E-09
11	2803154	0	T/A	0.014	0.67	0.11	1.87E-09
11	2803214	0	G/A	0.014	0.67	0.11	1.87E-09
11	2803222	0	A/G	0.014	0.67	0.11	1.87E-09
11	2803287	0	G/C	0.014	0.67	0.11	1.87E-09
11	2803371	0	T/G	0.014	0.67	0.11	1.87E-09
11	2803863	0	T/C	0.014	0.67	0.11	1.87E-09
11	2803999	0	T/C	0.014	0.67	0.11	1.87E-09
11	2804021	0	T/C	0.014	0.67	0.11	1.87E-09
11	2804035	0	C/A	0.014	0.67	0.11	1.87E-09
11	2804072	0	C/T	0.014	0.67	0.11	1.87E-09
11	2804082	0	T/C	0.014	0.67	0.11	1.87E-09
11	2804224	0	A/G	0.014	0.67	0.11	1.87E-09
11	2804226	0	T/C	0.014	0.67	0.11	1.87E-09
11	2805263	0	G/A	0.014	0.67	0.11	1.87E-09
11	2805266	0	C/G	0.014	0.67	0.11	1.87E-09
11	2805267	0	C/G	0.014	0.67	0.11	1.87E-09
11	2805314	0	C/T	0.014	0.67	0.11	1.87E-09
11	2805509	0	A/G	0.014	0.67	0.11	1.87E-09
11	2805569	0	T/G	0.014	0.67	0.11	1.87E-09
11	2805648	0	T/C	0.014	0.67	0.11	1.87E-09
11	2805710	0	T/A	0.014	0.67	0.11	1.87E-09
11	2805729	0	A/G	0.014	0.67	0.11	1.87E-09
11	2805880	0	T/C	0.014	0.67	0.11	1.87E-09
11	2806597	0	G/A	0.014	0.67	0.11	1.87E-09
11	2806750	0	T/C	0.014	0.67	0.11	1.87E-09
11	2808388	0	G/A	0.014	0.67	0.11	1.87E-09
11	2808401	0	G/A	0.014	0.67	0.11	1.87E-09

11	2808766	0	C/T	0.014	0.67	0.11	1.87E-09
11	2810456	0	G/T	0.014	0.67	0.11	1.87E-09
11	2810980	0	T/C	0.014	0.67	0.11	1.87E-09
11	2811188	0	T/C	0.014	0.67	0.11	1.87E-09
11	2811932	0	G/T	0.014	0.67	0.11	1.87E-09
11	2812637	0	G/A	0.014	0.67	0.11	1.87E-09
11	2813395	0	G/T	0.014	0.67	0.11	1.87E-09
11	2814008	0	A/G	0.014	0.67	0.11	1.87E-09
11	2814054	0	C/T	0.014	0.67	0.11	1.87E-09
11	2815089	0	G/C	0.014	0.67	0.11	1.87E-09
11	2815250	0	T/C	0.014	0.67	0.11	1.87E-09
11	2815649	0	C/A	0.014	0.67	0.11	1.87E-09
11	2815853	0	G/C	0.014	0.67	0.11	1.87E-09
11	2816161	0	T/G	0.014	0.67	0.11	1.87E-09
11	2816187	0	A/G	0.014	0.67	0.11	1.87E-09
11	2816378	0	A/G	0.014	0.67	0.11	1.87E-09
11	2816450	0	T/C	0.014	0.67	0.11	1.87E-09
11	2816496	0	A/G	0.014	0.67	0.11	1.87E-09
11	2816783	0	C/A	0.014	0.67	0.11	1.87E-09
11	2816824	0	A/G	0.014	0.67	0.11	1.87E-09
11	2816841	0	T/C	0.014	0.67	0.11	1.87E-09
11	2816883	0	A/G	0.014	0.67	0.11	1.87E-09
11	2816905	0	A/G	0.014	0.67	0.11	1.87E-09
11	2816908	0	G/C	0.014	0.67	0.11	1.87E-09
11	2817050	0	G/A	0.014	0.67	0.11	1.87E-09
11	2817222	0	G/A	0.014	0.67	0.11	1.87E-09
11	2817236	0	C/T	0.014	0.67	0.11	1.87E-09
11	2817400	0	T/C	0.014	0.67	0.11	1.87E-09
11	2817746	0	A/G	0.014	0.67	0.11	1.87E-09
11	2817943	0	T/A	0.014	0.67	0.11	1.87E-09
11	2818000	0	G/T	0.014	0.67	0.11	1.87E-09
11	2818232	0	C/T	0.014	0.67	0.11	1.87E-09
11	2819218	0	G/A	0.014	0.67	0.11	1.87E-09
11	2820601	0	A/C	0.014	0.67	0.11	1.87E-09
11	2821757	0	T/C	0.014	0.67	0.11	1.87E-09
11	2822082	0	T/C	0.014	0.67	0.11	1.87E-09
11	2823888	0	G/T	0.014	0.67	0.11	1.87E-09
11	2824576	0	T/C	0.014	0.67	0.11	1.87E-09
11	2824794	0	G/A	0.014	0.67	0.11	1.87E-09
11	2825534	0	A/G	0.014	0.67	0.11	1.87E-09

11	2825727	0	G/A	0.014	0.67	0.11	1.87E-09
11	2825775	0	A/C	0.014	0.67	0.11	1.87E-09
11	2825836	0	C/G	0.014	0.67	0.11	1.87E-09
11	2825876	0	C/T	0.014	0.67	0.11	1.87E-09
11	2825933	0	A/G	0.014	0.67	0.11	1.87E-09
11	2826136	0	G/A	0.014	0.67	0.11	1.87E-09
11	2826714	0	G/C	0.014	0.67	0.11	1.87E-09
11	2826903	0	A/C	0.014	0.67	0.11	1.87E-09
11	2826957	0	C/A	0.014	0.67	0.11	1.87E-09
11	2827098	0	A/C	0.014	0.67	0.11	1.87E-09
11	2827135	0	A/G	0.014	0.67	0.11	1.87E-09
11	2827348	0	G/C	0.014	0.67	0.11	1.87E-09
11	2827499	0	A/G	0.014	0.67	0.11	1.87E-09
11	2827532	0	A/T	0.014	0.67	0.11	1.87E-09
11	2827533	0	T/C	0.014	0.67	0.11	1.87E-09
11	2827626	0	G/T	0.014	0.67	0.11	1.87E-09
11	2828075	0	A/G	0.014	0.67	0.11	1.87E-09
11	2829444	0	C/T	0.014	0.67	0.11	1.87E-09
11	2830436	0	G/C	0.014	0.67	0.11	1.87E-09
11	2830550	0	T/G	0.014	0.67	0.11	1.87E-09
11	2831131	0	T/G	0.014	0.67	0.11	1.87E-09
11	2831291	0	A/G	0.014	0.67	0.11	1.87E-09
11	2831293	0	A/C	0.014	0.67	0.11	1.87E-09
11	2831329	0	T/C	0.014	0.67	0.11	1.87E-09
11	2831373	0	T/C	0.014	0.67	0.11	1.87E-09
11	2831418	0	G/C	0.014	0.67	0.11	1.87E-09
11	2832143	0	T/C	0.014	0.67	0.11	1.87E-09
11	2832625	0	A/G	0.014	0.67	0.11	1.87E-09
11	2833573	0	T/C	0.014	0.67	0.11	1.87E-09
11	2833698	0	A/G	0.014	0.67	0.11	1.87E-09
11	2833725	0	A/G	0.014	0.67	0.11	1.87E-09
11	2834464	0	T/C	0.014	0.67	0.11	1.87E-09
11	2834472	0	G/A	0.014	0.67	0.11	1.87E-09
11	2834502	0	T/C	0.014	0.67	0.11	1.87E-09
11	2834826	0	A/G	0.014	0.67	0.11	1.87E-09
11	2834829	0	T/C	0.014	0.67	0.11	1.87E-09
11	2834862	0	G/T	0.014	0.67	0.11	1.87E-09
11	2834922	0	C/T	0.014	0.67	0.11	1.87E-09
11	2835160	0	C/T	0.014	0.67	0.11	1.87E-09
11	2835188	0	T/C	0.014	0.67	0.11	1.87E-09

11	2835263	0	T/C	0.014	0.67	0.11	1.87E-09
11	2835297	0	A/G	0.014	0.67	0.11	1.87E-09
11	2835353	0	T/C	0.014	0.67	0.11	1.87E-09
11	2835358	0	T/C	0.014	0.67	0.11	1.87E-09
11	2835407	0	T/C	0.014	0.67	0.11	1.87E-09
11	2835861	0	T/C	0.014	0.67	0.11	1.87E-09
11	2835938	0	A/G	0.014	0.67	0.11	1.87E-09
11	2835962	0	A/G	0.014	0.67	0.11	1.87E-09
11	2836212	0	T/C	0.014	0.67	0.11	1.87E-09
11	2836382	0	C/T	0.014	0.67	0.11	1.87E-09
11	2837154	0	T/C	0.014	0.67	0.11	1.87E-09
11	2837312	0	A/G	0.014	0.67	0.11	1.87E-09
11	2837325	0	A/T	0.014	0.67	0.11	1.87E-09
11	2837400	0	A/T	0.014	0.67	0.11	1.87E-09
11	2837599	0	A/G	0.014	0.67	0.11	1.87E-09
11	2837770	0	G/A	0.014	0.67	0.11	1.87E-09
11	2837911	0	A/G	0.014	0.67	0.11	1.87E-09
11	2837920	0	A/G	0.014	0.67	0.11	1.87E-09
11	2838864	0	A/C	0.014	0.67	0.11	1.87E-09
11	2839126	0	C/G	0.014	0.67	0.11	1.87E-09
11	2839134	0	T/G	0.014	0.67	0.11	1.87E-09
11	2839256	0	C/T	0.014	0.67	0.11	1.87E-09
11	2839386	0	G/A	0.014	0.67	0.11	1.87E-09
11	2839501	0	G/C	0.014	0.67	0.11	1.87E-09
11	2839574	0	A/G	0.014	0.67	0.11	1.87E-09
11	2839580	0	A/G	0.014	0.67	0.11	1.87E-09
11	2839588	0	T/C	0.014	0.67	0.11	1.87E-09
11	2839598	0	G/C	0.014	0.67	0.11	1.87E-09
11	2839601	0	G/C	0.014	0.67	0.11	1.87E-09
11	2839663	0	G/A	0.014	0.67	0.11	1.87E-09
11	2839671	0	T/G	0.014	0.67	0.11	1.87E-09
11	2839730	0	A/G	0.014	0.67	0.11	1.87E-09
11	2839735	0	T/C	0.014	0.67	0.11	1.87E-09
11	2839888	0	G/A	0.014	0.67	0.11	1.87E-09
11	2840765	0	G/C	0.014	0.67	0.11	1.87E-09
11	2841176	0	A/G	0.014	0.67	0.11	1.87E-09
11	2841185	0	T/C	0.014	0.67	0.11	1.87E-09
11	2841332	0	C/T	0.014	0.67	0.11	1.87E-09
11	2841336	0	A/G	0.014	0.67	0.11	1.87E-09
11	2841657	0	A/G	0.014	0.67	0.11	1.87E-09

11	2841663	0	A/G	0.014	0.67	0.11	1.87E-09
11	2841665	0	T/C	0.014	0.67	0.11	1.87E-09
11	2841675	0	A/G	0.014	0.67	0.11	1.87E-09
11	2841701	0	A/T	0.014	0.67	0.11	1.87E-09
11	2841985	0	A/G	0.014	0.67	0.11	1.87E-09
11	2842002	0	C/G	0.014	0.67	0.11	1.87E-09
11	2842155	0	A/G	0.014	0.67	0.11	1.87E-09
11	2842249	0	T/A	0.014	0.67	0.11	1.87E-09
11	2842350	0	A/G	0.014	0.67	0.11	1.87E-09
11	2842464	0	A/G	0.014	0.67	0.11	1.87E-09
11	2842500	0	A/G	0.014	0.67	0.11	1.87E-09
11	2842607	0	T/C	0.014	0.67	0.11	1.87E-09
11	2842625	0	G/C	0.014	0.67	0.11	1.87E-09
11	2842626	0	G/A	0.014	0.67	0.11	1.87E-09
11	2842902	0	T/C	0.014	0.67	0.11	1.87E-09
11	2842935	0	C/T	0.014	0.67	0.11	1.87E-09
11	2842944	0	A/C	0.014	0.67	0.11	1.87E-09
11	2843031	0	T/C	0.014	0.67	0.11	1.87E-09
11	2843044	0	A/G	0.014	0.67	0.11	1.87E-09
11	2843049	0	G/C	0.014	0.67	0.11	1.87E-09
11	2843083	0	C/T	0.014	0.67	0.11	1.87E-09
11	2843312	0	C/T	0.014	0.67	0.11	1.87E-09
11	2843422	0	A/G	0.014	0.67	0.11	1.87E-09
11	2843624	0	G/A	0.014	0.67	0.11	1.87E-09
11	2843645	0	A/T	0.014	0.67	0.11	1.87E-09
11	2843670	0	T/G	0.014	0.67	0.11	1.87E-09
11	2843676	0	G/A	0.014	0.67	0.11	1.87E-09
11	2843746	0	C/T	0.014	0.67	0.11	1.87E-09
11	2843772	0	T/C	0.014	0.67	0.11	1.87E-09
11	2843773	0	G/A	0.014	0.67	0.11	1.87E-09
11	2843797	0	C/T	0.014	0.67	0.11	1.87E-09
11	2843826	0	C/G	0.014	0.67	0.11	1.87E-09
11	2843888	0	G/C	0.014	0.67	0.11	1.87E-09
11	2844447	0	T/C	0.014	0.67	0.11	1.87E-09
11	2844668	0	A/G	0.014	0.67	0.11	1.87E-09
11	2845333	0	T/C	0.014	0.67	0.11	1.87E-09
11	2845749	0	T/C	0.014	0.67	0.11	1.87E-09
11	2846644	0	A/G	0.014	0.67	0.11	1.87E-09
11	2847170	0	C/G	0.014	0.67	0.11	1.87E-09
11	2848266	0	A/C	0.014	0.67	0.11	1.87E-09

11	2848274	0	T/G	0.014	0.67	0.11	1.87E-09
11	2849478	0	T/C	0.014	0.67	0.11	1.87E-09
11	2849877	0	A/G	0.014	0.67	0.11	1.87E-09
11	2849952	0	A/G	0.014	0.67	0.11	1.87E-09
11	2850469	0	T/C	0.014	0.67	0.11	1.87E-09
11	2850575	0	A/G	0.014	0.67	0.11	1.87E-09
11	2850665	0	C/A	0.014	0.67	0.11	1.87E-09
11	2850887	0	G/T	0.014	0.67	0.11	1.87E-09
11	2851331	0	C/T	0.014	0.67	0.11	1.87E-09
11	2851879	0	T/C	0.014	0.67	0.11	1.87E-09
11	2851911	0	C/G	0.014	0.67	0.11	1.87E-09
11	2851919	0	A/G	0.014	0.67	0.11	1.87E-09
11	2852076	0	G/T	0.014	0.67	0.11	1.87E-09
11	2852191	0	T/C	0.014	0.67	0.11	1.87E-09
11	2852278	0	T/A	0.014	0.67	0.11	1.87E-09
11	2852561	0	A/G	0.014	0.67	0.11	1.87E-09
11	2852567	0	A/C	0.014	0.67	0.11	1.87E-09
11	2852575	0	A/T	0.014	0.67	0.11	1.87E-09
11	2852587	0	T/C	0.014	0.67	0.11	1.87E-09
11	2852642	0	A/C	0.014	0.67	0.11	1.87E-09
11	2852657	0	G/A	0.014	0.67	0.11	1.87E-09
11	2852660	0	T/G	0.014	0.67	0.11	1.87E-09
11	2852799	0	C/T	0.014	0.67	0.11	1.87E-09
11	2852844	0	C/T	0.014	0.67	0.11	1.87E-09
11	2852845	0	A/G	0.014	0.67	0.11	1.87E-09
11	2852917	0	A/G	0.014	0.67	0.11	1.87E-09
11	2852935	0	T/C	0.014	0.67	0.11	1.87E-09
11	2853393	0	T/C	0.014	0.67	0.11	1.87E-09
11	2853669	0	G/A	0.014	0.67	0.11	1.87E-09
11	2853820	0	T/C	0.014	0.67	0.11	1.87E-09
11	2853884	0	T/C	0.014	0.67	0.11	1.87E-09
11	2853965	0	G/A	0.014	0.67	0.11	1.87E-09
11	2854012	0	A/G	0.014	0.67	0.11	1.87E-09
11	2854939	0	A/G	0.014	0.67	0.11	1.87E-09
11	2854941	0	G/A	0.014	0.67	0.11	1.87E-09
11	2856074	0	T/C	0.014	0.67	0.11	1.87E-09
11	2856303	0	A/G	0.014	0.67	0.11	1.87E-09
11	2856612	0	T/C	0.014	0.67	0.11	1.87E-09
11	2859041	0	A/G	0.014	0.67	0.11	1.87E-09
11	2859908	0	T/C	0.014	0.67	0.11	1.87E-09

11	2860381	0	A/G	0.014	0.67	0.11	1.87E-09
11	2860494	0	T/C	0.014	0.67	0.11	1.87E-09
11	2860495	0	G/A	0.014	0.67	0.11	1.87E-09
11	2860506	0	C/T	0.014	0.67	0.11	1.87E-09
11	2860887	0	T/C	0.014	0.67	0.11	1.87E-09
11	2861201	0	G/A	0.014	0.67	0.11	1.87E-09
11	2861267	0	A/G	0.014	0.67	0.11	1.87E-09
11	2861343	0	T/G	0.014	0.67	0.11	1.87E-09
11	2861516	0	A/G	0.014	0.67	0.11	1.87E-09
11	2861612	0	G/T	0.014	0.67	0.11	1.87E-09
11	2862246	0	T/C	0.014	0.67	0.11	1.87E-09
11	2863322	0	C/T	0.014	0.67	0.11	1.87E-09
11	2863323	0	A/G	0.014	0.67	0.11	1.87E-09
11	2864368	0	T/C	0.014	0.67	0.11	1.87E-09
11	2864377	0	T/C	0.014	0.67	0.11	1.87E-09
11	2864795	0	A/G	0.014	0.67	0.11	1.87E-09
11	2864981	0	T/C	0.014	0.67	0.11	1.87E-09
11	2866040	0	C/T	0.014	0.67	0.11	1.87E-09
11	2866983	0	C/A	0.014	0.67	0.11	1.87E-09
11	2867106	0	C/T	0.014	0.67	0.11	1.87E-09
11	2869188	0	C/G	0.014	0.67	0.11	1.87E-09
11	2869864	0	G/A	0.014	0.67	0.11	1.87E-09
11	2870034	0	A/G	0.014	0.67	0.11	1.87E-09
11	2870742	0	C/T	0.014	0.67	0.11	1.87E-09
11	2870874	0	C/T	0.014	0.67	0.11	1.87E-09
11	2871003	0	A/G	0.014	0.67	0.11	1.87E-09
11	2871314	0	C/G	0.014	0.67	0.11	1.87E-09
11	2871650	0	T/C	0.014	0.67	0.11	1.87E-09
11	2871744	0	C/T	0.014	0.67	0.11	1.87E-09
11	2872006	0	A/G	0.014	0.67	0.11	1.87E-09
11	2872216	0	T/C	0.014	0.67	0.11	1.87E-09
11	2872491	0	A/G	0.014	0.67	0.11	1.87E-09
11	2873432	0	T/C	0.014	0.67	0.11	1.87E-09
11	2873908	0	G/A	0.014	0.67	0.11	1.87E-09
11	2873935	0	A/G	0.014	0.67	0.11	1.87E-09
11	2874203	0	A/G	0.014	0.67	0.11	1.87E-09
11	2874212	0	C/G	0.014	0.67	0.11	1.87E-09
11	2874392	0	A/G	0.014	0.67	0.11	1.87E-09
11	2874527	0	A/G	0.014	0.67	0.11	1.87E-09
11	2874856	0	T/C	0.014	0.67	0.11	1.87E-09

11	2875173	0	T/C	0.014	0.67	0.11	1.87E-09
11	2876218	0	A/G	0.014	0.67	0.11	1.87E-09
11	2876772	0	T/G	0.014	0.67	0.11	1.87E-09
11	2877037	0	A/G	0.014	0.67	0.11	1.87E-09
11	2877349	0	C/T	0.014	0.67	0.11	1.87E-09
11	2877414	0	T/C	0.014	0.67	0.11	1.87E-09
11	2877623	0	G/A	0.014	0.67	0.11	1.87E-09
11	2878017	0	A/G	0.014	0.67	0.11	1.87E-09
11	2878019	0	A/G	0.014	0.67	0.11	1.87E-09
11	2878075	0	G/A	0.014	0.67	0.11	1.87E-09
11	2878219	0	G/A	0.014	0.67	0.11	1.87E-09
11	2878245	0	T/G	0.014	0.67	0.11	1.87E-09
11	2878247	0	T/C	0.014	0.67	0.11	1.87E-09
11	2878248	0	A/T	0.014	0.67	0.11	1.87E-09
11	2878253	0	T/G	0.014	0.67	0.11	1.87E-09
11	2878616	0	C/T	0.014	0.67	0.11	1.87E-09
11	2878658	0	G/A	0.014	0.67	0.11	1.87E-09
11	2878677	0	T/C	0.014	0.67	0.11	1.87E-09
11	2878690	0	T/G	0.014	0.67	0.11	1.87E-09
11	2878727	0	C/A	0.014	0.67	0.11	1.87E-09
11	2878742	0	C/T	0.014	0.67	0.11	1.87E-09
11	2878753	0	A/G	0.014	0.67	0.11	1.87E-09
11	2879662	0	G/A	0.014	0.67	0.11	1.87E-09
11	2879767	0	C/T	0.014	0.67	0.11	1.87E-09
11	2879770	0	G/A	0.014	0.67	0.11	1.87E-09
11	2879790	0	C/T	0.014	0.67	0.11	1.87E-09
11	2879826	0	G/T	0.014	0.67	0.11	1.87E-09
11	2879834	0	C/A	0.014	0.67	0.11	1.87E-09
11	2879843	0	G/A	0.014	0.67	0.11	1.87E-09
11	2879858	0	G/A	0.014	0.67	0.11	1.87E-09
11	2879880	0	A/G	0.014	0.67	0.11	1.87E-09
11	2879904	0	C/T	0.014	0.67	0.11	1.87E-09
11	2879923	0	G/C	0.014	0.67	0.11	1.87E-09
11	2879941	0	T/C	0.014	0.67	0.11	1.87E-09
11	2880246	0	G/A	0.014	0.67	0.11	1.87E-09
11	2880498	0	A/T	0.014	0.67	0.11	1.87E-09
11	2880536	0	C/T	0.014	0.67	0.11	1.87E-09
11	2880672	0	G/T	0.014	0.67	0.11	1.87E-09
11	2880898	0	T/C	0.014	0.67	0.11	1.87E-09
11	2880911	0	T/C	0.014	0.67	0.11	1.87E-09

11	2880940	0	C/T	0.014	0.67	0.11	1.87E-09
11	2880941	0	A/G	0.014	0.67	0.11	1.87E-09
11	2880961	0	G/A	0.014	0.67	0.11	1.87E-09
11	2881055	0	C/T	0.014	0.67	0.11	1.87E-09
11	2881073	0	A/G	0.014	0.67	0.11	1.87E-09
11	2881157	0	C/T	0.014	0.67	0.11	1.87E-09
11	2881240	0	T/A	0.014	0.67	0.11	1.87E-09
11	2881332	0	A/G	0.014	0.67	0.11	1.87E-09
11	2881825	0	C/G	0.014	0.67	0.11	1.87E-09
11	2881902	0	C/T	0.014	0.67	0.11	1.87E-09
11	2881964	0	G/A	0.014	0.67	0.11	1.87E-09
11	2882216	0	T/C	0.014	0.67	0.11	1.87E-09
11	2882224	0	A/C	0.014	0.67	0.11	1.87E-09
11	2882255	0	T/C	0.014	0.67	0.11	1.87E-09
11	2882278	0	G/A	0.014	0.67	0.11	1.87E-09
11	2882286	0	T/G	0.014	0.67	0.11	1.87E-09
11	2882356	0	C/G	0.014	0.67	0.11	1.87E-09
11	2882365	0	G/C	0.014	0.67	0.11	1.87E-09
11	2882643	0	C/G	0.014	0.67	0.11	1.87E-09
11	2882681	0	C/A	0.014	0.67	0.11	1.87E-09
11	2882804	0	C/T	0.014	0.67	0.11	1.87E-09
11	2883073	0	G/T	0.014	0.67	0.11	1.87E-09
11	2883215	0	C/G	0.014	0.67	0.11	1.87E-09
11	2883344	0	T/C	0.014	0.67	0.11	1.87E-09
11	2883888	0	C/T	0.014	0.67	0.11	1.87E-09
11	2883997	0	T/C	0.014	0.67	0.11	1.87E-09
11	2884114	0	T/C	0.014	0.67	0.11	1.87E-09
11	2884873	0	T/C	0.014	0.67	0.11	1.87E-09
11	2885345	0	A/G	0.014	0.67	0.11	1.87E-09
11	2886432	0	G/A	0.014	0.67	0.11	1.87E-09
11	2887027	0	T/C	0.014	0.67	0.11	1.87E-09
11	2887054	0	G/A	0.014	0.67	0.11	1.87E-09
11	2887204	0	T/C	0.014	0.67	0.11	1.87E-09
11	2887601	0	T/C	0.014	0.67	0.11	1.87E-09
11	2887808	0	A/C	0.014	0.67	0.11	1.87E-09
11	2887872	0	C/T	0.014	0.67	0.11	1.87E-09
11	2888049	0	T/C	0.014	0.67	0.11	1.87E-09
11	2888312	0	A/G	0.014	0.67	0.11	1.87E-09
11	2888321	0	C/G	0.014	0.67	0.11	1.87E-09
11	2888329	0	A/G	0.014	0.67	0.11	1.87E-09

11	2888383	0	C/T	0.014	0.67	0.11	1.87E-09
11	2888594	0	G/A	0.014	0.67	0.11	1.87E-09
11	2888642	0	A/G	0.014	0.67	0.11	1.87E-09
11	2888682	0	G/A	0.014	0.67	0.11	1.87E-09
11	2888697	0	T/C	0.014	0.67	0.11	1.87E-09
11	2888839	0	C/T	0.014	0.67	0.11	1.87E-09
11	2888850	0	G/A	0.014	0.67	0.11	1.87E-09
11	2888966	0	G/C	0.014	0.67	0.11	1.87E-09
11	2889008	0	T/C	0.014	0.67	0.11	1.87E-09
11	2889036	0	A/C	0.014	0.67	0.11	1.87E-09
11	2889046	0	A/T	0.014	0.67	0.11	1.87E-09
11	2889053	0	C/G	0.014	0.67	0.11	1.87E-09
11	2889104	0	A/G	0.014	0.67	0.11	1.87E-09
11	2889122	0	A/G	0.014	0.67	0.11	1.87E-09
11	2889160	0	C/G	0.014	0.67	0.11	1.87E-09
11	2889167	0	A/G	0.014	0.67	0.11	1.87E-09
11	2889191	0	G/T	0.014	0.67	0.11	1.87E-09
11	2889199	0	A/G	0.014	0.67	0.11	1.87E-09
11	2889218	0	G/A	0.014	0.67	0.11	1.87E-09
11	2889235	0	T/C	0.014	0.67	0.11	1.87E-09
11	2889266	0	A/G	0.014	0.67	0.11	1.87E-09
11	2889311	0	C/T	0.014	0.67	0.11	1.87E-09
11	2889347	0	A/G	0.014	0.67	0.11	1.87E-09
11	2889371	0	G/A	0.014	0.67	0.11	1.87E-09
11	2889422	0	A/G	0.014	0.67	0.11	1.87E-09
11	2889504	0	T/C	0.014	0.67	0.11	1.87E-09
11	2889588	0	A/G	0.014	0.67	0.11	1.87E-09
11	2890073	0	G/C	0.014	0.67	0.11	1.87E-09
11	2890107	0	G/C	0.014	0.67	0.11	1.87E-09
11	2890301	0	T/C	0.014	0.67	0.11	1.87E-09
11	2890430	0	C/T	0.014	0.67	0.11	1.87E-09
11	2890434	0	C/A	0.014	0.67	0.11	1.87E-09
11	2890466	0	C/T	0.014	0.67	0.11	1.87E-09
11	2890745	0	C/T	0.014	0.67	0.11	1.87E-09
11	2890826	0	T/G	0.014	0.67	0.11	1.87E-09
11	2890899	0	C/T	0.014	0.67	0.11	1.87E-09
11	2890967	0	G/T	0.014	0.67	0.11	1.87E-09
11	2891018	0	G/A	0.014	0.67	0.11	1.87E-09
11	2891032	0	G/A	0.014	0.67	0.11	1.87E-09
11	2891071	0	T/C	0.014	0.67	0.11	1.87E-09

11	2891122	0	C/T	0.014	0.67	0.11	1.87E-09
11	2891123	0	T/C	0.014	0.67	0.11	1.87E-09
11	2891147	0	G/A	0.014	0.67	0.11	1.87E-09
11	2891243	0	C/G	0.014	0.67	0.11	1.87E-09
11	2891244	0	C/T	0.014	0.67	0.11	1.87E-09
11	2891316	0	G/A	0.014	0.67	0.11	1.87E-09
11	2891441	0	C/T	0.014	0.67	0.11	1.87E-09
11	2891460	0	A/G	0.014	0.67	0.11	1.87E-09
11	2891502	0	G/A	0.014	0.67	0.11	1.87E-09
11	2891506	0	A/G	0.014	0.67	0.11	1.87E-09
11	2891515	0	T/C	0.014	0.67	0.11	1.87E-09
11	2891568	0	C/T	0.014	0.67	0.11	1.87E-09
11	2891575	0	A/T	0.014	0.67	0.11	1.87E-09
11	2891595	0	A/G	0.014	0.67	0.11	1.87E-09
11	2891628	0	A/G	0.014	0.67	0.11	1.87E-09
11	2891717	0	T/C	0.014	0.67	0.11	1.87E-09
11	2891744	0	A/G	0.014	0.67	0.11	1.87E-09
11	2891751	0	T/A	0.014	0.67	0.11	1.87E-09
11	2891752	0	G/T	0.014	0.67	0.11	1.87E-09
11	2891754	0	G/T	0.014	0.67	0.11	1.87E-09
11	2891860	0	G/T	0.014	0.67	0.11	1.87E-09
11	2891887	0	G/A	0.014	0.67	0.11	1.87E-09
11	2891961	0	C/T	0.014	0.67	0.11	1.87E-09
11	2891980	0	G/T	0.014	0.67	0.11	1.87E-09
11	2891995	0	T/C	0.014	0.67	0.11	1.87E-09
11	2892009	0	C/G	0.014	0.67	0.11	1.87E-09
11	2892040	0	G/C	0.014	0.67	0.11	1.87E-09
11	2892210	0	C/T	0.014	0.67	0.11	1.87E-09
11	2892224	0	C/A	0.014	0.67	0.11	1.87E-09
11	2892242	0	A/C	0.014	0.67	0.11	1.87E-09
11	2892280	0	C/T	0.014	0.67	0.11	1.87E-09
11	2892281	0	C/G	0.014	0.67	0.11	1.87E-09
11	2892302	0	G/A	0.014	0.67	0.11	1.87E-09
11	2892354	0	G/A	0.014	0.67	0.11	1.87E-09
11	2892357	0	C/T	0.014	0.67	0.11	1.87E-09
11	2892398	0	G/A	0.014	0.67	0.11	1.87E-09
11	2892460	0	A/G	0.014	0.67	0.11	1.87E-09
11	2892493	0	G/A	0.014	0.67	0.11	1.87E-09
11	2892899	0	A/G	0.014	0.67	0.11	1.87E-09
11	2892935	0	A/T	0.014	0.67	0.11	1.87E-09

11	2893369	0	A/C	0.014	0.67	0.11	1.87E-09
11	2893392	0	C/T	0.014	0.67	0.11	1.87E-09
11	2893571	0	T/C	0.014	0.67	0.11	1.87E-09
11	2893585	0	T/C	0.014	0.67	0.11	1.87E-09
11	2893586	0	G/C	0.014	0.67	0.11	1.87E-09
11	2893671	0	A/G	0.014	0.67	0.11	1.87E-09
11	2893772	0	T/C	0.014	0.67	0.11	1.87E-09
11	2893780	0	A/G	0.014	0.67	0.11	1.87E-09
11	2893814	0	G/T	0.014	0.67	0.11	1.87E-09
11	2893842	0	A/G	0.014	0.67	0.11	1.87E-09
11	2893881	0	G/A	0.014	0.67	0.11	1.87E-09
11	2894015	0	A/G	0.014	0.67	0.11	1.87E-09
11	2894030	0	A/G	0.014	0.67	0.11	1.87E-09
11	2894045	0	C/T	0.014	0.67	0.11	1.87E-09
11	2894159	0	A/G	0.014	0.67	0.11	1.87E-09
11	2894235	0	A/G	0.014	0.67	0.11	1.87E-09
11	2894371	0	G/A	0.014	0.67	0.11	1.87E-09
11	2894433	0	A/G	0.014	0.67	0.11	1.87E-09
11	2894539	0	G/A	0.014	0.67	0.11	1.87E-09
11	2894569	0	G/A	0.014	0.67	0.11	1.87E-09
11	2894616	0	T/C	0.014	0.67	0.11	1.87E-09
11	2894758	0	C/T	0.014	0.67	0.11	1.87E-09
11	2894940	0	C/A	0.014	0.67	0.11	1.87E-09
11	2895002	0	A/G	0.014	0.67	0.11	1.87E-09
11	2895029	0	A/G	0.014	0.67	0.11	1.87E-09
11	2895143	0	A/G	0.014	0.67	0.11	1.87E-09
11	2895389	0	C/T	0.014	0.67	0.11	1.87E-09
11	2895873	0	C/T	0.014	0.67	0.11	1.87E-09
11	2896550	0	G/A	0.014	0.67	0.11	1.87E-09
11	2896591	0	T/C	0.014	0.67	0.11	1.87E-09
11	2897245	0	T/C	0.014	0.67	0.11	1.87E-09
11	2897395	0	G/T	0.014	0.67	0.11	1.87E-09
11	2897624	0	A/G	0.014	0.67	0.11	1.87E-09
11	2897750	0	T/C	0.014	0.67	0.11	1.87E-09
11	2898065	0	G/A	0.014	0.67	0.11	1.87E-09
11	2898066	0	C/T	0.014	0.67	0.11	1.87E-09
11	2898069	0	T/A	0.014	0.67	0.11	1.87E-09
11	2898137	0	A/G	0.014	0.67	0.11	1.87E-09
11	2898167	0	A/G	0.014	0.67	0.11	1.87E-09
11	2898454	0	T/C	0.014	0.67	0.11	1.87E-09

11	2898975	0	A/G	0.014	0.67	0.11	1.87E-09
11	2899025	0	T/C	0.014	0.67	0.11	1.87E-09
11	2901715	0	G/C	0.014	0.67	0.11	1.87E-09
11	2903271	0	A/C	0.014	0.67	0.11	1.87E-09
11	2903311	0	C/G	0.014	0.67	0.11	1.87E-09
11	2903356	0	A/G	0.014	0.67	0.11	1.87E-09
11	2903765	0	C/G	0.014	0.67	0.11	1.87E-09
11	2903785	0	C/T	0.014	0.67	0.11	1.87E-09
11	2903864	0	G/T	0.014	0.67	0.11	1.87E-09
11	2903892	0	T/A	0.014	0.67	0.11	1.87E-09
11	2903894	0	G/A	0.014	0.67	0.11	1.87E-09
11	2904025	0	T/C	0.014	0.67	0.11	1.87E-09
11	2904033	0	C/T	0.014	0.67	0.11	1.87E-09
11	2904059	0	G/A	0.014	0.67	0.11	1.87E-09
11	2904097	0	G/A	0.014	0.67	0.11	1.87E-09
11	2904151	0	A/G	0.014	0.67	0.11	1.87E-09
11	2904287	0	A/G	0.014	0.67	0.11	1.87E-09
11	2904429	0	A/G	0.014	0.67	0.11	1.87E-09
11	2904685	0	A/G	0.014	0.67	0.11	1.87E-09
11	2904716	0	A/G	0.014	0.67	0.11	1.87E-09
11	2905330	0	A/G	0.014	0.67	0.11	1.87E-09
11	2905339	0	C/T	0.014	0.67	0.11	1.87E-09
11	2905381	0	A/T	0.014	0.67	0.11	1.87E-09
11	2905402	0	G/A	0.014	0.67	0.11	1.87E-09
11	2905516	0	T/C	0.014	0.67	0.11	1.87E-09
11	2905518	0	A/G	0.014	0.67	0.11	1.87E-09
11	2905737	0	C/T	0.014	0.67	0.11	1.87E-09
11	2906246	0	C/G	0.014	0.67	0.11	1.87E-09
11	2906314	0	A/G	0.014	0.67	0.11	1.87E-09
11	2906356	0	T/C	0.014	0.67	0.11	1.87E-09
11	2906630	0	G/C	0.014	0.67	0.11	1.87E-09
11	2907543	0	G/A	0.014	0.67	0.11	1.87E-09
11	2909801	0	A/C	0.014	0.67	0.11	1.87E-09
11	2909877	0	A/G	0.014	0.67	0.11	1.87E-09
11	2909921	0	G/T	0.014	0.67	0.11	1.87E-09
11	2910112	0	G/C	0.014	0.67	0.11	1.87E-09
11	2910122	0	A/G	0.014	0.67	0.11	1.87E-09
11	2911047	0	G/A	0.014	0.67	0.11	1.87E-09
11	2911270	0	G/T	0.014	0.67	0.11	1.87E-09
11	2911271	0	A/T	0.014	0.67	0.11	1.87E-09

11	2911284	0	T/C	0.014	0.67	0.11	1.87E-09
11	2911489	0	T/C	0.014	0.67	0.11	1.87E-09
11	2911502	0	C/T	0.014	0.67	0.11	1.87E-09
11	2911565	0	T/A	0.014	0.67	0.11	1.87E-09
11	2911566	0	T/C	0.014	0.67	0.11	1.87E-09
11	2911601	0	G/A	0.014	0.67	0.11	1.87E-09
11	2911613	0	T/A	0.014	0.67	0.11	1.87E-09
11	2911638	0	A/G	0.014	0.67	0.11	1.87E-09
11	2911721	0	G/A	0.014	0.67	0.11	1.87E-09
11	2913118	0	C/T	0.014	0.67	0.11	1.87E-09
11	2913120	0	A/G	0.014	0.67	0.11	1.87E-09
11	2913283	0	C/T	0.014	0.67	0.11	1.87E-09
11	2913319	0	G/C	0.014	0.67	0.11	1.87E-09
11	2913441	0	A/G	0.014	0.67	0.11	1.87E-09
11	2913670	0	G/A	0.014	0.67	0.11	1.87E-09
11	2914314	0	C/A	0.014	0.67	0.11	1.87E-09
11	2914325	0	C/T	0.014	0.67	0.11	1.87E-09
11	2914351	0	G/A	0.014	0.67	0.11	1.87E-09
11	2914493	0	T/G	0.014	0.67	0.11	1.87E-09
11	2914568	0	T/C	0.014	0.67	0.11	1.87E-09
11	2914663	0	G/T	0.014	0.67	0.11	1.87E-09
11	2914674	0	C/T	0.014	0.67	0.11	1.87E-09
11	2914696	0	T/C	0.014	0.67	0.11	1.87E-09
11	2914807	0	A/G	0.014	0.67	0.11	1.87E-09
11	2914811	0	A/G	0.014	0.67	0.11	1.87E-09
11	2914862	0	T/C	0.014	0.67	0.11	1.87E-09
11	2914869	0	T/C	0.014	0.67	0.11	1.87E-09
11	2914875	0	C/A	0.014	0.67	0.11	1.87E-09
11	2914876	0	T/G	0.014	0.67	0.11	1.87E-09
11	2915128	0	A/C	0.014	0.67	0.11	1.87E-09
11	2915138	0	G/C	0.014	0.67	0.11	1.87E-09
11	2915164	0	T/C	0.014	0.67	0.11	1.87E-09
11	2915182	0	A/G	0.014	0.67	0.11	1.87E-09
11	2915196	0	A/G	0.014	0.67	0.11	1.87E-09
11	2915198	0	A/G	0.014	0.67	0.11	1.87E-09
11	2915220	0	G/T	0.014	0.67	0.11	1.87E-09
11	2915241	0	A/G	0.014	0.67	0.11	1.87E-09
11	2915271	0	A/G	0.014	0.67	0.11	1.87E-09
11	2915288	0	A/G	0.014	0.67	0.11	1.87E-09
11	2915301	0	A/G	0.014	0.67	0.11	1.87E-09

11	2915413	0	C/T	0.014	0.67	0.11	1.87E-09
11	2915414	0	C/G	0.014	0.67	0.11	1.87E-09
11	2915473	0	A/G	0.014	0.67	0.11	1.87E-09
11	2915550	0	A/T	0.014	0.67	0.11	1.87E-09
11	2915601	0	T/C	0.014	0.67	0.11	1.87E-09
11	2915620	0	A/G	0.014	0.67	0.11	1.87E-09
11	2915680	0	A/G	0.014	0.67	0.11	1.87E-09
11	2915785	0	A/G	0.014	0.67	0.11	1.87E-09
11	2915819	0	G/A	0.014	0.67	0.11	1.87E-09
11	2916011	0	A/G	0.014	0.67	0.11	1.87E-09
11	2916073	0	T/C	0.014	0.67	0.11	1.87E-09
11	2916174	0	T/C	0.014	0.67	0.11	1.87E-09
11	2916249	0	C/T	0.014	0.67	0.11	1.87E-09
11	2916313	0	G/A	0.014	0.67	0.11	1.87E-09
11	2916449	0	G/T	0.014	0.67	0.11	1.87E-09
11	2916474	0	C/T	0.014	0.67	0.11	1.87E-09
11	2916479	0	G/T	0.014	0.67	0.11	1.87E-09
11	2916497	0	G/T	0.014	0.67	0.11	1.87E-09
11	2916548	0	C/T	0.014	0.67	0.11	1.87E-09
11	2916611	0	C/G	0.014	0.67	0.11	1.87E-09
11	2916612	0	A/G	0.014	0.67	0.11	1.87E-09
11	2916661	0	G/A	0.014	0.67	0.11	1.87E-09
11	2916685	0	C/T	0.014	0.67	0.11	1.87E-09
11	2916703	0	G/T	0.014	0.67	0.11	1.87E-09
11	2916727	0	T/C	0.014	0.67	0.11	1.87E-09
11	2916737	0	A/G	0.014	0.67	0.11	1.87E-09
11	2916754	0	G/T	0.014	0.67	0.11	1.87E-09
11	2916763	0	T/C	0.014	0.67	0.11	1.87E-09
11	2916775	0	T/G	0.014	0.67	0.11	1.87E-09
11	2916781	0	G/C	0.014	0.67	0.11	1.87E-09
11	2916800	0	A/C	0.014	0.67	0.11	1.87E-09
11	2916901	0	T/G	0.014	0.67	0.11	1.87E-09
11	2917487	0	A/G	0.014	0.67	0.11	1.87E-09
11	2917732	0	G/A	0.014	0.67	0.11	1.87E-09
11	2918277	0	T/A	0.014	0.67	0.11	1.87E-09
11	2918375	0	G/A	0.014	0.67	0.11	1.87E-09
11	2918376	0	C/T	0.014	0.67	0.11	1.87E-09
11	2918409	0	A/G	0.014	0.67	0.11	1.87E-09
11	2918412	0	G/C	0.014	0.67	0.11	1.87E-09
11	2918472	0	G/A	0.014	0.67	0.11	1.87E-09

11	2918671	0	A/G	0.014	0.67	0.11	1.87E-09
11	2918708	0	T/C	0.014	0.67	0.11	1.87E-09
11	2918730	0	T/C	0.014	0.67	0.11	1.87E-09
11	2918970	0	C/T	0.014	0.67	0.11	1.87E-09
11	2919011	0	G/T	0.014	0.67	0.11	1.87E-09
11	2919043	0	T/G	0.014	0.67	0.11	1.87E-09
11	2919222	0	G/A	0.014	0.67	0.11	1.87E-09
11	2919249	0	A/G	0.014	0.67	0.11	1.87E-09
11	2920098	0	C/A	0.014	0.67	0.11	1.87E-09
11	2920385	0	G/A	0.014	0.67	0.11	1.87E-09
11	2920492	0	A/C	0.014	0.67	0.11	1.87E-09
11	2920533	0	C/T	0.014	0.67	0.11	1.87E-09
11	2920610	0	T/G	0.014	0.67	0.11	1.87E-09
11	2920613	0	G/C	0.014	0.67	0.11	1.87E-09
11	2920858	0	C/T	0.014	0.67	0.11	1.87E-09
11	2920862	0	C/G	0.014	0.67	0.11	1.87E-09
11	2920876	0	C/T	0.014	0.67	0.11	1.87E-09
11	2920884	0	A/G	0.014	0.67	0.11	1.87E-09
11	2921098	0	A/G	0.014	0.67	0.11	1.87E-09
11	2921141	0	G/A	0.014	0.67	0.11	1.87E-09
11	2921158	0	A/G	0.014	0.67	0.11	1.87E-09
11	2921222	0	G/T	0.014	0.67	0.11	1.87E-09
11	2921363	0	T/C	0.014	0.67	0.11	1.87E-09
11	2921383	0	C/G	0.014	0.67	0.11	1.87E-09
11	2921477	0	T/C	0.014	0.67	0.11	1.87E-09
11	2921663	0	T/C	0.014	0.67	0.11	1.87E-09
11	2921743	0	G/A	0.014	0.67	0.11	1.87E-09
11	2922130	0	G/A	0.014	0.67	0.11	1.87E-09
11	2922138	0	A/G	0.014	0.67	0.11	1.87E-09
11	2922139	0	G/A	0.014	0.67	0.11	1.87E-09
11	2922652	0	A/G	0.014	0.67	0.11	1.87E-09
11	2923515	0	T/C	0.014	0.67	0.11	1.87E-09
11	2923526	0	T/A	0.014	0.67	0.11	1.87E-09
11	2924104	0	A/G	0.014	0.67	0.11	1.87E-09
11	2924515	0	A/G	0.014	0.67	0.11	1.87E-09
11	2925328	0	C/A	0.014	0.67	0.11	1.87E-09
11	2925464	0	C/T	0.014	0.67	0.11	1.87E-09
11	2925600	0	T/C	0.014	0.67	0.11	1.87E-09
11	2925768	0	G/A	0.014	0.67	0.11	1.87E-09
11	2926155	0	C/T	0.014	0.67	0.11	1.87E-09

11	2926190	0	G/A	0.014	0.67	0.11	1.87E-09
11	2926348	0	G/A	0.014	0.67	0.11	1.87E-09
11	2926352	0	A/G	0.014	0.67	0.11	1.87E-09
11	2926432	0	T/C	0.014	0.67	0.11	1.87E-09
11	2926507	0	G/A	0.014	0.67	0.11	1.87E-09
11	2926602	0	A/T	0.014	0.67	0.11	1.87E-09
11	2926605	0	A/G	0.014	0.67	0.11	1.87E-09
11	2926870	0	G/A	0.014	0.67	0.11	1.87E-09
11	2926874	0	T/G	0.014	0.67	0.11	1.87E-09
11	2926942	0	T/C	0.014	0.67	0.11	1.87E-09
11	2926994	0	G/C	0.014	0.67	0.11	1.87E-09
11	2928516	0	C/G	0.014	0.67	0.11	1.87E-09
11	2929015	0	T/C	0.014	0.67	0.11	1.87E-09
11	2930426	0	G/C	0.014	0.67	0.11	1.87E-09
11	2931148	0	A/G	0.014	0.67	0.11	1.87E-09
11	2931304	0	A/T	0.014	0.67	0.11	1.87E-09
11	2931818	0	T/G	0.014	0.67	0.11	1.87E-09
11	2932375	0	G/C	0.014	0.67	0.11	1.87E-09
11	2934201	0	T/C	0.014	0.67	0.11	1.87E-09
11	2934409	0	A/T	0.014	0.67	0.11	1.87E-09
11	2934589	0	C/A	0.014	0.67	0.11	1.87E-09
11	2934622	0	A/G	0.014	0.67	0.11	1.87E-09
11	2935173	0	C/A	0.014	0.67	0.11	1.87E-09
11	2935606	0	T/C	0.014	0.67	0.11	1.87E-09
11	2936280	0	T/G	0.014	0.67	0.11	1.87E-09
11	2936321	0	C/G	0.014	0.67	0.11	1.87E-09
11	2936412	0	T/A	0.014	0.67	0.11	1.87E-09
11	2936450	0	G/C	0.014	0.67	0.11	1.87E-09
11	2936476	0	A/T	0.014	0.67	0.11	1.87E-09
11	2936478	0	C/T	0.014	0.67	0.11	1.87E-09
11	2936479	0	A/G	0.014	0.67	0.11	1.87E-09
11	2936484	0	G/A	0.014	0.67	0.11	1.87E-09
11	2936505	0	G/A	0.014	0.67	0.11	1.87E-09
11	2936602	0	T/G	0.014	0.67	0.11	1.87E-09
11	2936633	0	G/C	0.014	0.67	0.11	1.87E-09
11	2936720	0	G/T	0.014	0.67	0.11	1.87E-09
11	2936849	0	T/C	0.014	0.67	0.11	1.87E-09
11	2937233	0	A/G	0.014	0.67	0.11	1.87E-09
11	2937236	0	T/G	0.014	0.67	0.11	1.87E-09
11	2937583	0	T/G	0.014	0.67	0.11	1.87E-09

11	2937813	0	G/C	0.014	0.67	0.11	1.87E-09
11	2937832	0	T/C	0.014	0.67	0.11	1.87E-09
11	2938379	0	C/T	0.014	0.67	0.11	1.87E-09
11	2938532	0	T/G	0.014	0.67	0.11	1.87E-09
11	2938574	0	T/C	0.014	0.67	0.11	1.87E-09
11	2938865	0	G/C	0.014	0.67	0.11	1.87E-09
11	2939027	0	A/G	0.014	0.67	0.11	1.87E-09
11	2939054	0	T/A	0.014	0.67	0.11	1.87E-09
11	2939154	0	G/A	0.014	0.67	0.11	1.87E-09
11	2940157	0	T/C	0.014	0.67	0.11	1.87E-09
11	2940178	0	T/C	0.014	0.67	0.11	1.87E-09
11	2940207	0	T/C	0.014	0.67	0.11	1.87E-09
11	2940214	0	A/C	0.014	0.67	0.11	1.87E-09
11	2940257	0	C/T	0.014	0.67	0.11	1.87E-09
11	2940263	0	G/A	0.014	0.67	0.11	1.87E-09
11	2940278	0	A/G	0.014	0.67	0.11	1.87E-09
11	2940317	0	C/A	0.014	0.67	0.11	1.87E-09
11	2940887	0	T/C	0.014	0.67	0.11	1.87E-09
11	2942877	0	A/G	0.014	0.67	0.11	1.87E-09
11	2943546	0	A/G	0.014	0.67	0.11	1.87E-09
11	2943642	0	A/G	0.014	0.67	0.11	1.87E-09
11	2945363	0	G/A	0.014	0.67	0.11	1.87E-09
11	2946578	0	T/C	0.014	0.67	0.11	1.87E-09
11	2947230	0	A/G	0.014	0.67	0.11	1.87E-09
11	2947306	0	G/T	0.014	0.67	0.11	1.87E-09
11	2947700	0	C/G	0.014	0.67	0.11	1.87E-09
11	2947739	0	T/C	0.014	0.67	0.11	1.87E-09
11	2947740	0	A/C	0.014	0.67	0.11	1.87E-09
11	2947986	0	T/C	0.014	0.67	0.11	1.87E-09
11	2948113	0	A/G	0.014	0.67	0.11	1.87E-09
11	2948192	0	T/C	0.014	0.67	0.11	1.87E-09
11	2948197	0	T/G	0.014	0.67	0.11	1.87E-09
11	2948247	0	A/G	0.014	0.67	0.11	1.87E-09
11	2948314	0	C/G	0.014	0.67	0.11	1.87E-09
11	2948344	0	T/C	0.014	0.67	0.11	1.87E-09
11	2948424	0	T/C	0.014	0.67	0.11	1.87E-09
11	2948618	0	T/C	0.014	0.67	0.11	1.87E-09
11	2950061	0	A/G	0.014	0.67	0.11	1.87E-09
11	2950075	0	A/G	0.014	0.67	0.11	1.87E-09
11	2950336	0	G/A	0.014	0.67	0.11	1.87E-09

11	2950340	0	G/A	0.014	0.67	0.11	1.87E-09
11	2951697	0	A/C	0.014	0.67	0.11	1.87E-09
11	2951806	0	C/T	0.014	0.67	0.11	1.87E-09
11	2951879	0	G/T	0.014	0.67	0.11	1.87E-09
11	2952482	0	C/A	0.014	0.67	0.11	1.87E-09
11	2952499	0	G/A	0.014	0.67	0.11	1.87E-09
11	2952507	0	A/G	0.014	0.67	0.11	1.87E-09
11	2957244	0	G/A	0.014	0.67	0.11	1.87E-09
11	2958909	0	C/T	0.014	0.67	0.11	1.87E-09
11	2959622	0	T/C	0.014	0.67	0.11	1.87E-09
11	2959924	0	G/A	0.014	0.67	0.11	1.87E-09
11	2959926	0	G/A	0.014	0.67	0.11	1.87E-09
11	2959927	0	G/A	0.014	0.67	0.11	1.87E-09
11	2960268	0	T/G	0.014	0.67	0.11	1.87E-09
11	2960424	0	A/G	0.014	0.67	0.11	1.87E-09
11	2960504	0	A/G	0.014	0.67	0.11	1.87E-09
11	2960525	0	A/G	0.014	0.67	0.11	1.87E-09
11	2960706	0	C/T	0.014	0.67	0.11	1.87E-09
11	2960957	0	G/A	0.014	0.67	0.11	1.87E-09
11	2962411	0	G/A	0.014	0.67	0.11	1.87E-09
11	2962834	0	T/C	0.014	0.67	0.11	1.87E-09
11	2962973	0	A/G	0.014	0.67	0.11	1.87E-09
11	2963057	0	T/C	0.014	0.67	0.11	1.87E-09
11	2963081	0	G/T	0.014	0.67	0.11	1.87E-09
11	2963772	0	G/T	0.014	0.67	0.11	1.87E-09
11	2963776	0	G/C	0.014	0.67	0.11	1.87E-09
11	2963815	0	A/G	0.014	0.67	0.11	1.87E-09
11	2964281	0	T/C	0.014	0.67	0.11	1.87E-09
11	2966235	0	T/C	0.014	0.67	0.11	1.87E-09
11	2966396	0	A/C	0.014	0.67	0.11	1.87E-09
11	2966558	0	A/G	0.014	0.67	0.11	1.87E-09
11	2967417	0	G/T	0.014	0.67	0.11	1.87E-09
11	2967871	0	C/T	0.014	0.67	0.11	1.87E-09
11	2968203	0	T/C	0.014	0.67	0.11	1.87E-09
11	2968454	0	C/T	0.014	0.67	0.11	1.87E-09
11	2968570	0	G/T	0.014	0.67	0.11	1.87E-09
11	2969111	0	A/C	0.014	0.67	0.11	1.87E-09
11	3038526	0	A/G	0.014	0.67	0.11	1.87E-09
11	3039333	0	A/G	0.014	0.67	0.11	1.87E-09
11	3039560	0	A/C	0.014	0.67	0.11	1.87E-09

11	3039614	0	A/G	0.014	0.67	0.11	1.87E-09
11	3040423	0	C/G	0.014	0.67	0.11	1.87E-09
11	3040614	0	A/G	0.014	0.67	0.11	1.87E-09
11	3040620	0	G/C	0.014	0.67	0.11	1.87E-09
11	3040621	0	G/C	0.014	0.67	0.11	1.87E-09
11	3040645	0	C/G	0.014	0.67	0.11	1.87E-09
11	3041394	0	G/C	0.014	0.67	0.11	1.87E-09
11	3041395	0	T/C	0.014	0.67	0.11	1.87E-09
11	3041756	0	A/C	0.014	0.67	0.11	1.87E-09
11	3041761	0	C/A	0.014	0.67	0.11	1.87E-09
11	3041911	0	T/C	0.014	0.67	0.11	1.87E-09
11	3041966	0	A/G	0.014	0.67	0.11	1.87E-09
11	3042110	0	C/G	0.014	0.67	0.11	1.87E-09
11	3042162	0	A/G	0.014	0.67	0.11	1.87E-09
11	3042336	0	C/G	0.014	0.67	0.11	1.87E-09
11	3042428	0	G/A	0.014	0.67	0.11	1.87E-09
11	3042447	0	C/T	0.014	0.67	0.11	1.87E-09
11	3042450	0	C/G	0.014	0.67	0.11	1.87E-09
11	3042458	0	C/T	0.014	0.67	0.11	1.87E-09
11	3042542	0	A/G	0.014	0.67	0.11	1.87E-09
11	3042565	0	T/G	0.014	0.67	0.11	1.87E-09
11	3042661	0	A/G	0.014	0.67	0.11	1.87E-09
11	3042796	0	A/G	0.014	0.67	0.11	1.87E-09
11	3043007	0	C/G	0.014	0.67	0.11	1.87E-09
11	3043042	0	C/T	0.014	0.67	0.11	1.87E-09
11	3043070	0	C/T	0.014	0.67	0.11	1.87E-09
11	3043236	0	C/T	0.014	0.67	0.11	1.87E-09
11	3043309	0	C/G	0.014	0.67	0.11	1.87E-09
11	3043328	0	G/T	0.014	0.67	0.11	1.87E-09
11	3043476	0	G/T	0.014	0.67	0.11	1.87E-09
11	3043480	0	C/A	0.014	0.67	0.11	1.87E-09
11	3043482	0	G/A	0.014	0.67	0.11	1.87E-09
11	3043585	0	A/T	0.014	0.67	0.11	1.87E-09
11	3043630	0	C/A	0.014	0.67	0.11	1.87E-09
11	3043910	0	C/T	0.014	0.67	0.11	1.87E-09
11	3043918	0	T/G	0.014	0.67	0.11	1.87E-09
11	3044539	0	C/G	0.014	0.67	0.11	1.87E-09
11	3045641	0	G/C	0.014	0.67	0.11	1.87E-09
11	3045733	0	C/T	0.014	0.67	0.11	1.87E-09
11	3045781	0	G/A	0.014	0.67	0.11	1.87E-09

11	3045792	0	C/T	0.014	0.67	0.11	1.87E-09
11	3045883	0	T/C	0.014	0.67	0.11	1.87E-09
11	3046462	0	T/C	0.014	0.67	0.11	1.87E-09
11	3046684	0	T/G	0.014	0.67	0.11	1.87E-09
11	3046741	0	G/A	0.014	0.67	0.11	1.87E-09
11	3046907	0	A/C	0.014	0.67	0.11	1.87E-09
11	3046932	0	T/C	0.014	0.67	0.11	1.87E-09
11	3047041	0	A/T	0.014	0.67	0.11	1.87E-09
11	3047207	0	C/T	0.014	0.67	0.11	1.87E-09
11	3047282	0	G/A	0.014	0.67	0.11	1.87E-09
11	3047296	0	G/A	0.014	0.67	0.11	1.87E-09
11	3047303	0	C/T	0.014	0.67	0.11	1.87E-09
11	3047352	0	C/A	0.014	0.67	0.11	1.87E-09
11	3047614	0	C/T	0.014	0.67	0.11	1.87E-09
11	3047744	0	G/A	0.014	0.67	0.11	1.87E-09
11	3047843	0	T/G	0.014	0.67	0.11	1.87E-09
11	3048004	0	A/G	0.014	0.67	0.11	1.87E-09
11	3048072	0	C/G	0.014	0.67	0.11	1.87E-09
11	3049553	0	A/G	0.014	0.67	0.11	1.87E-09
11	3049767	0	G/A	0.014	0.67	0.11	1.87E-09
11	3049943	0	G/A	0.014	0.67	0.11	1.87E-09
11	3051192	0	C/T	0.014	0.67	0.11	1.87E-09
11	3051415	0	G/A	0.014	0.67	0.11	1.87E-09
11	3051465	0	G/A	0.014	0.67	0.11	1.87E-09
11	3051528	0	G/A	0.014	0.67	0.11	1.87E-09
11	3051539	0	G/A	0.014	0.67	0.11	1.87E-09
11	3051563	0	A/G	0.014	0.67	0.11	1.87E-09
11	3051569	0	G/A	0.014	0.67	0.11	1.87E-09
11	3051709	0	G/A	0.014	0.67	0.11	1.87E-09
11	3051955	0	G/A	0.014	0.67	0.11	1.87E-09
11	3052060	0	G/A	0.014	0.67	0.11	1.87E-09
11	3052187	0	A/T	0.014	0.67	0.11	1.87E-09
11	3052210	0	C/T	0.014	0.67	0.11	1.87E-09
11	3052557	0	G/A	0.014	0.67	0.11	1.87E-09
11	3052658	0	T/A	0.014	0.67	0.11	1.87E-09
11	3052713	0	C/T	0.014	0.67	0.11	1.87E-09
11	3052768	0	C/G	0.014	0.67	0.11	1.87E-09
11	3052773	0	T/C	0.014	0.67	0.11	1.87E-09
11	3052775	0	C/G	0.014	0.67	0.11	1.87E-09
11	3052967	0	T/C	0.014	0.67	0.11	1.87E-09

11	3053019	0	A/G	0.014	0.67	0.11	1.87E-09
11	3053211	0	A/G	0.014	0.67	0.11	1.87E-09
11	3053238	0	T/C	0.014	0.67	0.11	1.87E-09
11	3053475	0	A/C	0.014	0.67	0.11	1.87E-09
11	3053679	0	T/G	0.014	0.67	0.11	1.87E-09
11	3053685	0	G/A	0.014	0.67	0.11	1.87E-09
11	3053713	0	T/C	0.014	0.67	0.11	1.87E-09
11	3053729	0	T/C	0.014	0.67	0.11	1.87E-09
11	3053750	0	A/G	0.014	0.67	0.11	1.87E-09
11	3054014	0	C/A	0.014	0.67	0.11	1.87E-09
11	3054039	0	A/G	0.014	0.67	0.11	1.87E-09
11	3054175	0	A/C	0.014	0.67	0.11	1.87E-09
11	3057497	0	G/A	0.014	0.67	0.11	1.87E-09
11	3057771	0	G/A	0.014	0.67	0.11	1.87E-09
11	3058298	0	C/T	0.014	0.67	0.11	1.87E-09
11	3058330	0	G/A	0.014	0.67	0.11	1.87E-09
11	3058331	0	G/A	0.014	0.67	0.11	1.87E-09
11	3058454	0	G/A	0.014	0.67	0.11	1.87E-09
11	3058576	0	G/A	0.014	0.67	0.11	1.87E-09
11	3058699	0	G/A	0.014	0.67	0.11	1.87E-09
11	3059068	0	G/A	0.014	0.67	0.11	1.87E-09
11	3059206	0	G/A	0.014	0.67	0.11	1.87E-09
11	3059287	0	C/A	0.014	0.67	0.11	1.87E-09
11	3059754	0	C/T	0.014	0.67	0.11	1.87E-09
11	3059928	0	G/T	0.014	0.67	0.11	1.87E-09
11	3060109	0	G/A	0.014	0.67	0.11	1.87E-09
11	3060139	0	G/A	0.014	0.67	0.11	1.87E-09
11	3060529	0	G/A	0.014	0.67	0.11	1.87E-09
11	3060542	0	G/T	0.014	0.67	0.11	1.87E-09
11	3060551	0	T/C	0.014	0.67	0.11	1.87E-09
11	3060651	0	C/T	0.014	0.67	0.11	1.87E-09
11	3060663	0	C/G	0.014	0.67	0.11	1.87E-09
11	3060697	0	G/A	0.014	0.67	0.11	1.87E-09
11	3060732	0	T/C	0.014	0.67	0.11	1.87E-09
11	3060818	0	G/A	0.014	0.67	0.11	1.87E-09
11	3062127	0	C/T	0.014	0.67	0.11	1.87E-09
11	3062306	0	G/A	0.014	0.67	0.11	1.87E-09
11	3062436	0	C/T	0.014	0.67	0.11	1.87E-09
11	3063011	0	C/T	0.014	0.67	0.11	1.87E-09
11	3064707	0	G/A	0.014	0.67	0.11	1.87E-09

11	3066699	0	A/G	0.014	0.67	0.11	1.87E-09
11	3067039	0	A/G	0.014	0.67	0.11	1.87E-09
11	3067410	0	A/G	0.014	0.67	0.11	1.87E-09
11	3067445	0	A/G	0.014	0.67	0.11	1.87E-09
11	3067449	0	A/G	0.014	0.67	0.11	1.87E-09
11	3067542	0	C/T	0.014	0.67	0.11	1.87E-09
11	3068020	0	A/G	0.014	0.67	0.11	1.87E-09
11	3068098	0	A/G	0.014	0.67	0.11	1.87E-09
11	3068145	0	T/C	0.014	0.67	0.11	1.87E-09
11	3068163	0	C/T	0.014	0.67	0.11	1.87E-09
11	3068352	0	T/G	0.014	0.67	0.11	1.87E-09
11	3068382	0	G/A	0.014	0.67	0.11	1.87E-09
11	3068847	0	C/G	0.014	0.67	0.11	1.87E-09
11	3069386	0	C/A	0.014	0.67	0.11	1.87E-09
11	3069788	0	T/C	0.014	0.67	0.11	1.87E-09
11	3070010	0	A/G	0.014	0.67	0.11	1.87E-09
11	3070111	0	C/G	0.014	0.67	0.11	1.87E-09
11	3070666	0	G/A	0.014	0.67	0.11	1.87E-09
11	3070671	0	A/G	0.014	0.67	0.11	1.87E-09
11	3071246	0	C/T	0.014	0.67	0.11	1.87E-09
11	3073570	0	A/G	0.014	0.67	0.11	1.87E-09
11	3074513	0	G/A	0.014	0.67	0.11	1.87E-09
11	3075781	0	A/G	0.014	0.67	0.11	1.87E-09
11	3075894	0	A/G	0.014	0.67	0.11	1.87E-09
11	3076688	0	T/C	0.014	0.67	0.11	1.87E-09
11	3076911	0	A/G	0.014	0.67	0.11	1.87E-09
11	3077210	0	C/T	0.014	0.67	0.11	1.87E-09
11	3077216	0	A/C	0.014	0.67	0.11	1.87E-09
11	3077693	0	A/G	0.014	0.67	0.11	1.87E-09
11	3077889	0	T/A	0.014	0.67	0.11	1.87E-09
11	3077941	0	C/A	0.014	0.67	0.11	1.87E-09
11	3077971	0	A/G	0.014	0.67	0.11	1.87E-09
11	3077982	0	T/C	0.014	0.67	0.11	1.87E-09
11	3078009	0	C/T	0.014	0.67	0.11	1.87E-09
11	3078014	0	T/C	0.014	0.67	0.11	1.87E-09
11	3078708	0	A/G	0.014	0.67	0.11	1.87E-09
11	3078753	0	G/C	0.014	0.67	0.11	1.87E-09
11	3078812	0	G/C	0.014	0.67	0.11	1.87E-09
11	3081695	0	C/A	0.014	0.67	0.11	1.87E-09
11	3082216	0	C/T	0.014	0.67	0.11	1.87E-09

11	3082812	0	C/T	0.014	0.67	0.11	1.87E-09
11	3082944	0	C/A	0.014	0.67	0.11	1.87E-09
11	3082949	0	C/T	0.014	0.67	0.11	1.87E-09
11	3083144	0	G/A	0.014	0.67	0.11	1.87E-09
11	3083147	0	G/T	0.014	0.67	0.11	1.87E-09
11	3083193	0	C/A	0.014	0.67	0.11	1.87E-09
11	3083201	0	G/A	0.014	0.67	0.11	1.87E-09
11	3086566	0	T/C	0.014	0.67	0.11	1.87E-09
11	3086572	0	C/T	0.014	0.67	0.11	1.87E-09
11	3086618	0	G/A	0.014	0.67	0.11	1.87E-09
11	3087180	0	A/T	0.014	0.67	0.11	1.87E-09
11	3087242	0	T/C	0.014	0.67	0.11	1.87E-09
11	3087278	0	A/G	0.014	0.67	0.11	1.87E-09
11	3087527	0	A/G	0.014	0.67	0.11	1.87E-09
11	3087579	0	T/C	0.014	0.67	0.11	1.87E-09
11	3087592	0	T/C	0.014	0.67	0.11	1.87E-09
11	3087629	0	C/T	0.014	0.67	0.11	1.87E-09
11	3087657	0	A/C	0.014	0.67	0.11	1.87E-09
11	3087726	0	T/C	0.014	0.67	0.11	1.87E-09
11	3087913	0	A/G	0.014	0.67	0.11	1.87E-09
11	3087974	0	A/G	0.014	0.67	0.11	1.87E-09
11	3088064	0	C/T	0.014	0.67	0.11	1.87E-09
11	3092774	0	T/C	0.014	0.67	0.11	1.87E-09
11	3093051	0	C/T	0.014	0.67	0.11	1.87E-09
11	3093756	0	G/A	0.014	0.67	0.11	1.87E-09
11	3093824	0	G/C	0.014	0.67	0.11	1.87E-09
11	3093836	0	G/A	0.014	0.67	0.11	1.87E-09
11	3094213	0	C/T	0.014	0.67	0.11	1.87E-09
11	3094220	0	G/A	0.014	0.67	0.11	1.87E-09
11	3094238	0	G/A	0.014	0.67	0.11	1.87E-09
11	3094245	0	G/A	0.014	0.67	0.11	1.87E-09
11	3094382	0	A/G	0.014	0.67	0.11	1.87E-09
11	3094413	0	T/C	0.014	0.67	0.11	1.87E-09
11	3094437	0	C/A	0.014	0.67	0.11	1.87E-09
11	3094474	0	G/A	0.014	0.67	0.11	1.87E-09
11	3094489	0	G/A	0.014	0.67	0.11	1.87E-09
11	3094501	0	G/A	0.014	0.67	0.11	1.87E-09
11	3094527	0	A/G	0.014	0.67	0.11	1.87E-09
11	3094529	0	T/C	0.014	0.67	0.11	1.87E-09
11	3094546	0	C/T	0.014	0.67	0.11	1.87E-09

11	3094732	0	G/C	0.014	0.67	0.11	1.87E-09
11	3094750	0	G/T	0.014	0.67	0.11	1.87E-09
11	3094758	0	G/T	0.014	0.67	0.11	1.87E-09
11	3095449	0	T/C	0.014	0.67	0.11	1.87E-09
11	3095787	0	G/A	0.014	0.67	0.11	1.87E-09
11	3095814	0	G/A	0.014	0.67	0.11	1.87E-09
11	3096263	0	T/C	0.014	0.67	0.11	1.87E-09
11	3096722	0	T/C	0.014	0.67	0.11	1.87E-09
11	3096775	0	T/C	0.014	0.67	0.11	1.87E-09
11	3097203	0	T/C	0.014	0.67	0.11	1.87E-09
11	3097215	0	C/T	0.014	0.67	0.11	1.87E-09
11	3097279	0	C/A	0.014	0.67	0.11	1.87E-09
11	3097521	0	C/T	0.014	0.67	0.11	1.87E-09
11	3097541	0	C/G	0.014	0.67	0.11	1.87E-09
11	3097729	0	C/T	0.014	0.67	0.11	1.87E-09
11	3097902	0	T/C	0.014	0.67	0.11	1.87E-09
11	3097998	0	C/T	0.014	0.67	0.11	1.87E-09
11	3098005	0	T/C	0.014	0.67	0.11	1.87E-09
11	3098203	0	C/A	0.014	0.67	0.11	1.87E-09
11	3098442	0	T/C	0.014	0.67	0.11	1.87E-09
11	3098573	0	G/A	0.014	0.67	0.11	1.87E-09
11	3098668	0	T/G	0.014	0.67	0.11	1.87E-09
11	3098699	0	A/G	0.014	0.67	0.11	1.87E-09
11	3098738	0	C/T	0.014	0.67	0.11	1.87E-09
11	3099245	0	G/A	0.014	0.67	0.11	1.87E-09
11	3099276	0	T/A	0.014	0.67	0.11	1.87E-09
11	3099284	0	T/C	0.014	0.67	0.11	1.87E-09
11	3099791	0	C/T	0.014	0.67	0.11	1.87E-09
11	3100248	0	T/C	0.014	0.67	0.11	1.87E-09
11	3100408	0	G/T	0.014	0.67	0.11	1.87E-09
11	3100426	0	T/G	0.014	0.67	0.11	1.87E-09
11	3100430	0	G/A	0.014	0.67	0.11	1.87E-09
11	3100901	0	T/C	0.014	0.67	0.11	1.87E-09
11	3101436	0	T/C	0.014	0.67	0.11	1.87E-09
11	3101491	0	T/C	0.014	0.67	0.11	1.87E-09
11	3101523	0	G/C	0.014	0.67	0.11	1.87E-09
11	3101536	0	G/T	0.014	0.67	0.11	1.87E-09
11	3101545	0	C/T	0.014	0.67	0.11	1.87E-09
11	3127156	0	C/T	0.014	0.67	0.11	1.87E-09
11	3128928	0	C/A	0.014	0.67	0.11	1.87E-09

11	3128929	0	G/T	0.014	0.67	0.11	1.87E-09
11	3129342	0	C/T	0.014	0.67	0.11	1.87E-09
11	3129954	0	C/T	0.014	0.67	0.11	1.87E-09
11	3130023	0	G/A	0.014	0.67	0.11	1.87E-09
11	3131029	0	G/A	0.014	0.67	0.11	1.87E-09
11	3131108	0	G/C	0.014	0.67	0.11	1.87E-09
11	3131111	0	C/G	0.014	0.67	0.11	1.87E-09
11	3131112	0	G/T	0.014	0.67	0.11	1.87E-09
11	3131205	0	C/T	0.014	0.67	0.11	1.87E-09
11	3131213	0	C/T	0.014	0.67	0.11	1.87E-09
11	3131346	0	A/C	0.014	0.67	0.11	1.87E-09
11	3131349	0	G/A	0.014	0.67	0.11	1.87E-09
11	3131417	0	G/A	0.014	0.67	0.11	1.87E-09
11	3131424	0	C/T	0.014	0.67	0.11	1.87E-09
11	3131620	0	C/T	0.014	0.67	0.11	1.87E-09
11	3131645	0	C/T	0.014	0.67	0.11	1.87E-09
11	3132298	0	C/T	0.014	0.67	0.11	1.87E-09
11	3134817	0	A/G	0.014	0.67	0.11	1.87E-09
11	3135150	0	A/T	0.014	0.67	0.11	1.87E-09
11	3135329	0	T/C	0.014	0.67	0.11	1.87E-09
11	3135359	0	C/T	0.014	0.67	0.11	1.87E-09
11	3135551	0	G/A	0.014	0.67	0.11	1.87E-09
11	3135579	0	C/T	0.014	0.67	0.11	1.87E-09
11	3135659	0	G/C	0.014	0.67	0.11	1.87E-09
11	3135702	0	A/G	0.014	0.67	0.11	1.87E-09
11	3135773	0	C/A	0.014	0.67	0.11	1.87E-09
11	3135826	0	G/A	0.014	0.67	0.11	1.87E-09
11	3136127	0	C/T	0.014	0.67	0.11	1.87E-09
11	3136291	0	T/C	0.014	0.67	0.11	1.87E-09
11	3136349	0	A/C	0.014	0.67	0.11	1.87E-09
11	3136361	0	G/T	0.014	0.67	0.11	1.87E-09
11	3136621	0	A/G	0.014	0.67	0.11	1.87E-09
11	3137303	0	A/G	0.014	0.67	0.11	1.87E-09
11	3137524	0	A/G	0.014	0.67	0.11	1.87E-09
11	3138862	0	T/C	0.014	0.67	0.11	1.87E-09
11	3139461	0	C/A	0.014	0.67	0.11	1.87E-09
11	3139567	0	G/A	0.014	0.67	0.11	1.87E-09
11	3139633	0	T/C	0.014	0.67	0.11	1.87E-09
11	3139728	0	T/C	0.014	0.67	0.11	1.87E-09
11	3139765	0	T/C	0.014	0.67	0.11	1.87E-09

11	3139930	0	G/A	0.014	0.67	0.11	1.87E-09
11	3139955	0	C/T	0.014	0.67	0.11	1.87E-09
11	3139956	0	G/A	0.014	0.67	0.11	1.87E-09
11	3140004	0	T/G	0.014	0.67	0.11	1.87E-09
11	3140010	0	A/G	0.014	0.67	0.11	1.87E-09
11	3140080	0	A/G	0.014	0.67	0.11	1.87E-09
11	3140168	0	G/A	0.014	0.67	0.11	1.87E-09
11	3140201	0	C/G	0.014	0.67	0.11	1.87E-09
11	3140319	0	A/G	0.014	0.67	0.11	1.87E-09
11	3140388	0	G/A	0.014	0.67	0.11	1.87E-09
11	3140395	0	A/C	0.014	0.67	0.11	1.87E-09
11	3140420	0	G/A	0.014	0.67	0.11	1.87E-09
11	3140435	0	C/T	0.014	0.67	0.11	1.87E-09
11	3140570	0	A/G	0.014	0.67	0.11	1.87E-09
11	3140696	0	A/G	0.014	0.67	0.11	1.87E-09
11	3140717	0	C/A	0.014	0.67	0.11	1.87E-09
11	3140754	0	G/A	0.014	0.67	0.11	1.87E-09
11	3141826	0	C/A	0.014	0.67	0.11	1.87E-09
11	3143194	0	T/C	0.014	0.67	0.11	1.87E-09
11	3149107	0	T/C	0.014	0.67	0.11	1.87E-09
11	3149118	0	A/G	0.014	0.67	0.11	1.87E-09
11	3149131	0	A/G	0.014	0.67	0.11	1.87E-09
11	3149133	0	T/C	0.014	0.67	0.11	1.87E-09
11	3149136	0	T/C	0.014	0.67	0.11	1.87E-09
11	3149148	0	T/A	0.014	0.67	0.11	1.87E-09
11	3149149	0	C/A	0.014	0.67	0.11	1.87E-09
11	3149171	0	T/A	0.014	0.67	0.11	1.87E-09
11	3149173	0	G/A	0.014	0.67	0.11	1.87E-09
11	3149247	0	A/G	0.014	0.67	0.11	1.87E-09
11	3149269	0	C/G	0.014	0.67	0.11	1.87E-09
11	3149398	0	C/T	0.014	0.67	0.11	1.87E-09
11	3149437	0	A/G	0.014	0.67	0.11	1.87E-09
11	3149466	0	T/G	0.014	0.67	0.11	1.87E-09
11	3149485	0	C/T	0.014	0.67	0.11	1.87E-09
11	3149486	0	A/G	0.014	0.67	0.11	1.87E-09
11	3149492	0	C/T	0.014	0.67	0.11	1.87E-09
11	3149678	0	C/T	0.014	0.67	0.11	1.87E-09
11	3149691	0	T/C	0.014	0.67	0.11	1.87E-09
11	3149839	0	T/C	0.014	0.67	0.11	1.87E-09
11	3149857	0	C/T	0.014	0.67	0.11	1.87E-09

11	3150018	0	G/C	0.014	0.67	0.11	1.87E-09
11	3150090	0	C/T	0.014	0.67	0.11	1.87E-09
11	3150192	0	T/C	0.014	0.67	0.11	1.87E-09
11	3150271	0	T/G	0.014	0.67	0.11	1.87E-09
11	3150384	0	G/A	0.014	0.67	0.11	1.87E-09
11	3150412	0	T/A	0.014	0.67	0.11	1.87E-09
11	3150467	0	T/C	0.014	0.67	0.11	1.87E-09
11	3150468	0	G/C	0.014	0.67	0.11	1.87E-09
11	3150474	0	T/C	0.014	0.67	0.11	1.87E-09
11	3150521	0	T/C	0.014	0.67	0.11	1.87E-09
11	3150586	0	T/C	0.014	0.67	0.11	1.87E-09
11	3150591	0	T/C	0.014	0.67	0.11	1.87E-09
11	3150592	0	G/A	0.014	0.67	0.11	1.87E-09
11	3150681	0	C/T	0.014	0.67	0.11	1.87E-09
11	3150753	0	C/T	0.014	0.67	0.11	1.87E-09
11	3150859	0	C/G	0.014	0.67	0.11	1.87E-09
11	3150864	0	A/G	0.014	0.67	0.11	1.87E-09
11	3150865	0	T/C	0.014	0.67	0.11	1.87E-09
11	3150943	0	C/T	0.014	0.67	0.11	1.87E-09
11	3150983	0	T/C	0.014	0.67	0.11	1.87E-09
11	3151019	0	C/T	0.014	0.67	0.11	1.87E-09
11	3151067	0	G/A	0.014	0.67	0.11	1.87E-09
11	3151237	0	G/A	0.014	0.67	0.11	1.87E-09
11	3151283	0	A/G	0.014	0.67	0.11	1.87E-09
11	3151335	0	G/A	0.014	0.67	0.11	1.87E-09
11	3151356	0	G/A	0.014	0.67	0.11	1.87E-09
11	3151376	0	G/A	0.014	0.67	0.11	1.87E-09
11	3151427	0	C/T	0.014	0.67	0.11	1.87E-09
11	3151465	0	A/G	0.014	0.67	0.11	1.87E-09
11	3151468	0	T/G	0.014	0.67	0.11	1.87E-09
11	3151477	0	T/G	0.014	0.67	0.11	1.87E-09
11	3151552	0	A/G	0.014	0.67	0.11	1.87E-09
11	3152071	0	T/C	0.014	0.67	0.11	1.87E-09
11	3152103	0	T/C	0.014	0.67	0.11	1.87E-09
11	3152195	0	C/T	0.014	0.67	0.11	1.87E-09
11	3152203	0	C/T	0.014	0.67	0.11	1.87E-09
11	3152805	0	C/T	0.014	0.67	0.11	1.87E-09
11	3152831	0	G/C	0.014	0.67	0.11	1.87E-09
11	3153480	0	T/C	0.014	0.67	0.11	1.87E-09
11	3153481	0	T/C	0.014	0.67	0.11	1.87E-09

11	3153495	0	T/C	0.014	0.67	0.11	1.87E-09
11	3153502	0	A/C	0.014	0.67	0.11	1.87E-09
11	3153540	0	T/C	0.014	0.67	0.11	1.87E-09
11	3153553	0	A/G	0.014	0.67	0.11	1.87E-09
11	3153699	0	A/G	0.014	0.67	0.11	1.87E-09
11	3153901	0	C/T	0.014	0.67	0.11	1.87E-09
11	3154785	0	C/T	0.014	0.67	0.11	1.87E-09
11	3154978	0	G/A	0.014	0.67	0.11	1.87E-09
11	3155023	0	A/G	0.014	0.67	0.11	1.87E-09
11	3155066	0	T/C	0.014	0.67	0.11	1.87E-09
11	3155250	0	T/G	0.014	0.67	0.11	1.87E-09
11	3155501	0	G/C	0.014	0.67	0.11	1.87E-09
11	3155727	0	T/C	0.014	0.67	0.11	1.87E-09
11	3155787	0	C/T	0.014	0.67	0.11	1.87E-09
11	3156330	0	G/C	0.014	0.67	0.11	1.87E-09
11	3156482	0	T/A	0.014	0.67	0.11	1.87E-09
11	3168561	0	T/C	0.014	0.67	0.11	1.87E-09
11	3170981	0	G/A	0.014	0.67	0.11	1.87E-09
11	3171127	0	C/G	0.014	0.67	0.11	1.87E-09
11	3171141	0	T/A	0.014	0.67	0.11	1.87E-09
11	3171186	0	C/G	0.014	0.67	0.11	1.87E-09
11	3171289	0	A/C	0.014	0.67	0.11	1.87E-09
11	3171311	0	C/G	0.014	0.67	0.11	1.87E-09
11	3171312	0	A/G	0.014	0.67	0.11	1.87E-09
11	3171398	0	A/G	0.014	0.67	0.11	1.87E-09
11	3171621	0	G/C	0.014	0.67	0.11	1.87E-09
11	3171638	0	T/C	0.014	0.67	0.11	1.87E-09
11	3171648	0	T/C	0.014	0.67	0.11	1.87E-09
11	3171738	0	C/G	0.014	0.67	0.11	1.87E-09
11	3171947	0	A/G	0.014	0.67	0.11	1.87E-09
11	3171959	0	A/G	0.014	0.67	0.11	1.87E-09
11	3171972	0	G/A	0.014	0.67	0.11	1.87E-09
11	3172017	0	G/A	0.014	0.67	0.11	1.87E-09
11	3172076	0	T/G	0.014	0.67	0.11	1.87E-09
11	3172149	0	A/G	0.014	0.67	0.11	1.87E-09
11	3172166	0	A/G	0.014	0.67	0.11	1.87E-09
11	3172198	0	C/A	0.014	0.67	0.11	1.87E-09
11	3172267	0	G/A	0.014	0.67	0.11	1.87E-09
11	3172421	0	C/T	0.014	0.67	0.11	1.87E-09
11	3172563	0	C/T	0.014	0.67	0.11	1.87E-09

11	3172851	0	G/A	0.014	0.67	0.11	1.87E-09
11	3172900	0	C/T	0.014	0.67	0.11	1.87E-09
11	3172982	0	T/C	0.014	0.67	0.11	1.87E-09
11	3173046	0	C/T	0.014	0.67	0.11	1.87E-09
11	3173090	0	G/C	0.014	0.67	0.11	1.87E-09
11	3173141	0	C/T	0.014	0.67	0.11	1.87E-09
11	3173147	0	G/A	0.014	0.67	0.11	1.87E-09
11	3173217	0	T/C	0.014	0.67	0.11	1.87E-09
11	3173325	0	A/G	0.014	0.67	0.11	1.87E-09
11	3173380	0	C/T	0.014	0.67	0.11	1.87E-09
11	3173397	0	G/T	0.014	0.67	0.11	1.87E-09
11	3173561	0	C/T	0.014	0.67	0.11	1.87E-09
11	3173571	0	T/C	0.014	0.67	0.11	1.87E-09
11	3173610	0	T/C	0.014	0.67	0.11	1.87E-09
11	3173711	0	T/A	0.014	0.67	0.11	1.87E-09
11	3173762	0	A/G	0.014	0.67	0.11	1.87E-09
11	3173872	0	G/A	0.014	0.67	0.11	1.87E-09
11	3174053	0	A/C	0.014	0.67	0.11	1.87E-09
11	3174065	0	G/A	0.014	0.67	0.11	1.87E-09
11	3174100	0	G/C	0.014	0.67	0.11	1.87E-09
11	3174104	0	A/G	0.014	0.67	0.11	1.87E-09
11	3174426	0	C/A	0.014	0.67	0.11	1.87E-09
11	3174443	0	C/T	0.014	0.67	0.11	1.87E-09
11	3174447	0	A/G	0.014	0.67	0.11	1.87E-09
11	3174524	0	A/G	0.014	0.67	0.11	1.87E-09
11	3174578	0	C/T	0.014	0.67	0.11	1.87E-09
11	3174878	0	T/G	0.014	0.67	0.11	1.87E-09
11	3174935	0	G/C	0.014	0.67	0.11	1.87E-09
11	3174945	0	C/T	0.014	0.67	0.11	1.87E-09
11	3174994	0	G/A	0.014	0.67	0.11	1.87E-09
11	3175035	0	C/T	0.014	0.67	0.11	1.87E-09
11	3175090	0	T/C	0.014	0.67	0.11	1.87E-09
11	3175102	0	G/A	0.014	0.67	0.11	1.87E-09
11	3175115	0	T/C	0.014	0.67	0.11	1.87E-09
11	3175120	0	G/C	0.014	0.67	0.11	1.87E-09
11	3175136	0	G/T	0.014	0.67	0.11	1.87E-09
11	3175146	0	G/A	0.014	0.67	0.11	1.87E-09
11	3175156	0	A/G	0.014	0.67	0.11	1.87E-09
11	3175173	0	T/C	0.014	0.67	0.11	1.87E-09
11	3175223	0	C/T	0.014	0.67	0.11	1.87E-09

11	3175236	0	T/C	0.014	0.67	0.11	1.87E-09
11	3175241	0	T/C	0.014	0.67	0.11	1.87E-09
11	3175255	0	A/G	0.014	0.67	0.11	1.87E-09
11	3175262	0	T/C	0.014	0.67	0.11	1.87E-09
11	3175267	0	A/G	0.014	0.67	0.11	1.87E-09
11	3175268	0	T/C	0.014	0.67	0.11	1.87E-09
11	3175281	0	T/C	0.014	0.67	0.11	1.87E-09
11	3175303	0	A/G	0.014	0.67	0.11	1.87E-09
11	3175328	0	C/T	0.014	0.67	0.11	1.87E-09
11	3175329	0	G/A	0.014	0.67	0.11	1.87E-09
11	3175432	0	T/C	0.014	0.67	0.11	1.87E-09
11	3175469	0	C/T	0.014	0.67	0.11	1.87E-09
11	3175570	0	A/G	0.014	0.67	0.11	1.87E-09
11	3175575	0	A/T	0.014	0.67	0.11	1.87E-09
11	3175715	0	C/T	0.014	0.67	0.11	1.87E-09
11	3175719	0	T/C	0.014	0.67	0.11	1.87E-09
11	3176451	0	G/A	0.014	0.67	0.11	1.87E-09
11	3176544	0	C/T	0.014	0.67	0.11	1.87E-09
11	3176652	0	G/T	0.014	0.67	0.11	1.87E-09
11	3176661	0	G/C	0.014	0.67	0.11	1.87E-09
11	3176819	0	T/C	0.014	0.67	0.11	1.87E-09
11	3176824	0	G/A	0.014	0.67	0.11	1.87E-09
11	3176922	0	T/C	0.014	0.67	0.11	1.87E-09
11	3176923	0	G/A	0.014	0.67	0.11	1.87E-09
11	3176926	0	T/C	0.014	0.67	0.11	1.87E-09
11	3176943	0	T/G	0.014	0.67	0.11	1.87E-09
11	3176965	0	G/A	0.014	0.67	0.11	1.87E-09
11	3176997	0	C/T	0.014	0.67	0.11	1.87E-09
11	3178112	0	C/T	0.014	0.67	0.11	1.87E-09
11	3178178	0	G/A	0.014	0.67	0.11	1.87E-09
11	3178232	0	A/C	0.014	0.67	0.11	1.87E-09
11	3178307	0	G/A	0.014	0.67	0.11	1.87E-09
11	3178333	0	A/C	0.014	0.67	0.11	1.87E-09
11	3178406	0	C/G	0.014	0.67	0.11	1.87E-09
11	3178416	0	C/T	0.014	0.67	0.11	1.87E-09
11	3178418	0	C/T	0.014	0.67	0.11	1.87E-09
11	3178429	0	G/C	0.014	0.67	0.11	1.87E-09
11	3178537	0	C/A	0.014	0.67	0.11	1.87E-09
11	3178607	0	A/G	0.014	0.67	0.11	1.87E-09
11	3178658	0	G/A	0.014	0.67	0.11	1.87E-09

11	3178688	0	G/A	0.014	0.67	0.11	1.87E-09
11	3178987	0	G/A	0.014	0.67	0.11	1.87E-09
11	3178994	0	T/C	0.014	0.67	0.11	1.87E-09
11	3179004	0	G/A	0.014	0.67	0.11	1.87E-09
11	3179212	0	G/A	0.014	0.67	0.11	1.87E-09
11	3179238	0	C/G	0.014	0.67	0.11	1.87E-09
11	3179429	0	A/T	0.014	0.67	0.11	1.87E-09
11	3179512	0	T/C	0.014	0.67	0.11	1.87E-09
11	3179544	0	G/A	0.014	0.67	0.11	1.87E-09
11	3179639	0	C/T	0.014	0.67	0.11	1.87E-09
11	3179771	0	T/C	0.014	0.67	0.11	1.87E-09
11	3179788	0	G/A	0.014	0.67	0.11	1.87E-09
11	3179805	0	G/A	0.014	0.67	0.11	1.87E-09
11	3179829	0	C/T	0.014	0.67	0.11	1.87E-09
11	3179834	0	T/G	0.014	0.67	0.11	1.87E-09
11	3183128	0	G/A	0.014	0.67	0.11	1.87E-09
11	3183210	0	G/T	0.014	0.67	0.11	1.87E-09
11	3183571	0	A/G	0.014	0.67	0.11	1.87E-09
11	3183576	0	C/T	0.014	0.67	0.11	1.87E-09
11	3183853	0	G/A	0.014	0.67	0.11	1.87E-09
11	3240402	0	G/A	0.014	0.67	0.11	1.87E-09
11	3240413	0	A/G	0.014	0.67	0.11	1.87E-09
11	3240577	0	T/G	0.014	0.67	0.11	1.87E-09
11	3240955	0	G/A	0.014	0.67	0.11	1.87E-09
11	3240986	0	G/A	0.014	0.67	0.11	1.87E-09
11	3240999	0	C/A	0.014	0.67	0.11	1.87E-09
11	3241149	0	A/C	0.014	0.67	0.11	1.87E-09
11	3241156	0	C/T	0.014	0.67	0.11	1.87E-09
11	3241163	0	C/T	0.014	0.67	0.11	1.87E-09
11	3241315	0	A/G	0.014	0.67	0.11	1.87E-09
11	3241333	0	A/G	0.014	0.67	0.11	1.87E-09
11	3241429	0	A/G	0.014	0.67	0.11	1.87E-09
11	3241470	0	T/C	0.014	0.67	0.11	1.87E-09
11	3241501	0	T/C	0.014	0.67	0.11	1.87E-09
11	3241532	0	G/A	0.014	0.67	0.11	1.87E-09
11	3241808	0	T/C	0.014	0.67	0.11	1.87E-09
11	3242164	0	G/A	0.014	0.67	0.11	1.87E-09
11	3242210	0	G/A	0.014	0.67	0.11	1.87E-09
11	3242265	0	C/A	0.014	0.67	0.11	1.87E-09
11	3242285	0	A/G	0.014	0.67	0.11	1.87E-09

11	3242302	0	G/A	0.014	0.67	0.11	1.87E-09
11	3242328	0	C/T	0.014	0.67	0.11	1.87E-09
11	3242357	0	C/A	0.014	0.67	0.11	1.87E-09
11	3242620	0	A/G	0.014	0.67	0.11	1.87E-09
11	3242650	0	G/A	0.014	0.67	0.11	1.87E-09
11	3242760	0	A/G	0.014	0.67	0.11	1.87E-09
11	3242864	0	C/A	0.014	0.67	0.11	1.87E-09
11	3242885	0	G/A	0.014	0.67	0.11	1.87E-09
11	3242903	0	A/G	0.014	0.67	0.11	1.87E-09
11	3242920	0	T/C	0.014	0.67	0.11	1.87E-09
11	3242950	0	A/G	0.014	0.67	0.11	1.87E-09
11	3242977	0	G/A	0.014	0.67	0.11	1.87E-09
11	3243042	0	T/G	0.014	0.67	0.11	1.87E-09
11	3243062	0	T/C	0.014	0.67	0.11	1.87E-09
11	3243063	0	G/A	0.014	0.67	0.11	1.87E-09
11	3243138	0	A/G	0.014	0.67	0.11	1.87E-09
11	3243149	0	C/T	0.014	0.67	0.11	1.87E-09
11	3243276	0	A/T	0.014	0.67	0.11	1.87E-09
11	3243277	0	A/T	0.014	0.67	0.11	1.87E-09
11	3243337	0	A/T	0.014	0.67	0.11	1.87E-09
11	3243412	0	C/T	0.014	0.67	0.11	1.87E-09
11	3243484	0	G/C	0.014	0.67	0.11	1.87E-09
11	3243568	0	T/C	0.014	0.67	0.11	1.87E-09
11	3243671	0	C/T	0.014	0.67	0.11	1.87E-09
11	3243697	0	A/G	0.014	0.67	0.11	1.87E-09
11	3243706	0	A/T	0.014	0.67	0.11	1.87E-09
11	3243815	0	A/G	0.014	0.67	0.11	1.87E-09
11	3243852	0	C/T	0.014	0.67	0.11	1.87E-09
11	3243864	0	G/T	0.014	0.67	0.11	1.87E-09
11	3243876	0	T/C	0.014	0.67	0.11	1.87E-09
11	3244498	0	A/G	0.014	0.67	0.11	1.87E-09
11	3244763	0	A/C	0.014	0.67	0.11	1.87E-09
11	3244774	0	T/C	0.014	0.67	0.11	1.87E-09
11	3254060	0	T/C	0.014	0.67	0.11	1.87E-09
11	3254113	0	C/T	0.014	0.67	0.11	1.87E-09
11	3254274	0	T/C	0.014	0.67	0.11	1.87E-09
11	3254330	0	A/C	0.014	0.67	0.11	1.87E-09
11	3254436	0	A/C	0.014	0.67	0.11	1.87E-09
11	3254730	0	A/G	0.014	0.67	0.11	1.87E-09
11	3255159	0	A/G	0.014	0.67	0.11	1.87E-09

11	3255226	0	G/A	0.014	0.67	0.11	1.87E-09
11	3255820	0	C/G	0.014	0.67	0.11	1.87E-09
11	3255886	0	C/A	0.014	0.67	0.11	1.87E-09
11	3255887	0	C/T	0.014	0.67	0.11	1.87E-09
11	3256112	0	A/G	0.014	0.67	0.11	1.87E-09
11	3256202	0	G/A	0.014	0.67	0.11	1.87E-09
11	3257914	0	A/G	0.014	0.67	0.11	1.87E-09
11	3258441	0	T/C	0.014	0.67	0.11	1.87E-09
11	3259598	0	T/A	0.014	0.67	0.11	1.87E-09
11	3259611	0	T/C	0.014	0.67	0.11	1.87E-09
11	3259620	0	A/G	0.014	0.67	0.11	1.87E-09
11	3259688	0	T/C	0.014	0.67	0.11	1.87E-09
11	3259779	0	T/C	0.014	0.67	0.11	1.87E-09
11	3259851	0	G/A	0.014	0.67	0.11	1.87E-09
11	3259902	0	A/C	0.014	0.67	0.11	1.87E-09
11	3259904	0	A/T	0.014	0.67	0.11	1.87E-09
11	3259905	0	C/G	0.014	0.67	0.11	1.87E-09
11	3259942	0	T/C	0.014	0.67	0.11	1.87E-09
11	3259961	0	A/G	0.014	0.67	0.11	1.87E-09
11	3261152	0	C/G	0.014	0.67	0.11	1.87E-09
11	3261188	0	C/T	0.014	0.67	0.11	1.87E-09
11	3261241	0	C/A	0.014	0.67	0.11	1.87E-09
11	3261728	0	T/G	0.014	0.67	0.11	1.87E-09
11	3261998	0	G/T	0.014	0.67	0.11	1.87E-09
11	3262972	0	A/G	0.014	0.67	0.11	1.87E-09
11	3268269	0	C/A	0.014	0.67	0.11	1.87E-09
11	3268465	0	G/T	0.014	0.67	0.11	1.87E-09
11	3269350	0	A/C	0.014	0.67	0.11	1.87E-09
11	3269814	0	A/G	0.014	0.67	0.11	1.87E-09
11	3269854	0	T/C	0.014	0.67	0.11	1.87E-09
11	3269976	0	C/T	0.014	0.67	0.11	1.87E-09
11	3270073	0	A/C	0.014	0.67	0.11	1.87E-09
11	3270206	0	C/T	0.014	0.67	0.11	1.87E-09
11	3270248	0	G/A	0.014	0.67	0.11	1.87E-09
11	3270322	0	A/C	0.014	0.67	0.11	1.87E-09
11	3270356	0	A/G	0.014	0.67	0.11	1.87E-09
11	3270359	0	C/T	0.014	0.67	0.11	1.87E-09
11	3270455	0	T/C	0.014	0.67	0.11	1.87E-09
11	3270463	0	C/T	0.014	0.67	0.11	1.87E-09
11	3270498	0	C/T	0.014	0.67	0.11	1.87E-09

11	3270510	0	T/C	0.014	0.67	0.11	1.87E-09
11	3270530	0	T/C	0.014	0.67	0.11	1.87E-09
11	3270532	0	G/A	0.014	0.67	0.11	1.87E-09
11	3270597	0	A/T	0.014	0.67	0.11	1.87E-09
11	3270608	0	G/T	0.014	0.67	0.11	1.87E-09
11	3270740	0	T/G	0.014	0.67	0.11	1.87E-09
11	3270797	0	A/G	0.014	0.67	0.11	1.87E-09
11	3270807	0	T/C	0.014	0.67	0.11	1.87E-09
11	3270888	0	C/T	0.014	0.67	0.11	1.87E-09
11	3270889	0	A/G	0.014	0.67	0.11	1.87E-09
11	3270913	0	C/T	0.014	0.67	0.11	1.87E-09
11	3271461	0	T/C	0.014	0.67	0.11	1.87E-09
11	3271828	0	C/A	0.014	0.67	0.11	1.87E-09
11	3271932	0	C/G	0.014	0.67	0.11	1.87E-09
11	3272166	0	A/G	0.014	0.67	0.11	1.87E-09
11	3272377	0	T/C	0.014	0.67	0.11	1.87E-09
11	3273656	0	G/A	0.014	0.67	0.11	1.87E-09
11	3278038	0	T/C	0.014	0.67	0.11	1.87E-09
11	3278106	0	T/G	0.014	0.67	0.11	1.87E-09
11	3278286	0	G/A	0.014	0.67	0.11	1.87E-09
11	3278466	0	G/T	0.014	0.67	0.11	1.87E-09
11	3278529	0	G/A	0.014	0.67	0.11	1.87E-09
11	3278695	0	C/G	0.014	0.67	0.11	1.87E-09
11	3281138	0	A/G	0.014	0.67	0.11	1.87E-09
11	3310077	0	C/G	0.014	0.67	0.11	1.87E-09
11	3310659	0	A/G	0.014	0.67	0.11	1.87E-09
11	3310695	0	T/A	0.014	0.67	0.11	1.87E-09
11	3310825	0	T/C	0.014	0.67	0.11	1.87E-09
11	3310942	0	A/G	0.014	0.67	0.11	1.87E-09
11	3312387	0	C/A	0.014	0.67	0.11	1.87E-09
11	3315067	0	T/C	0.014	0.67	0.11	1.87E-09
11	3315096	0	G/A	0.014	0.67	0.11	1.87E-09
11	3315312	0	A/G	0.014	0.67	0.11	1.87E-09
11	3315326	0	T/C	0.014	0.67	0.11	1.87E-09
11	3316008	0	C/T	0.014	0.67	0.11	1.87E-09
11	3317156	0	T/C	0.014	0.67	0.11	1.87E-09
11	3317349	0	G/A	0.014	0.67	0.11	1.87E-09
11	3318114	0	C/T	0.014	0.67	0.11	1.87E-09
11	3319068	0	C/T	0.014	0.67	0.11	1.87E-09
11	3319078	0	A/G	0.014	0.67	0.11	1.87E-09

11	3319518	0	C/T	0.014	0.67	0.11	1.87E-09
11	3319878	0	A/G	0.014	0.67	0.11	1.87E-09
11	3320833	0	T/C	0.014	0.67	0.11	1.87E-09
11	3321718	0	T/C	0.014	0.67	0.11	1.87E-09
11	3322611	0	T/A	0.014	0.67	0.11	1.87E-09
11	3325039	0	T/A	0.014	0.67	0.11	1.87E-09
11	3325203	0	A/T	0.014	0.67	0.11	1.87E-09
11	3325205	0	T/C	0.014	0.67	0.11	1.87E-09
11	3325352	0	T/C	0.014	0.67	0.11	1.87E-09
11	3325614	0	G/A	0.014	0.67	0.11	1.87E-09
11	3326518	0	A/G	0.014	0.67	0.11	1.87E-09
11	3326522	0	G/T	0.014	0.67	0.11	1.87E-09
11	3326550	0	C/T	0.014	0.67	0.11	1.87E-09
11	3326926	0	T/A	0.014	0.67	0.11	1.87E-09
11	3327852	0	T/C	0.014	0.67	0.11	1.87E-09
11	3328169	0	T/C	0.014	0.67	0.11	1.87E-09
11	3328311	0	C/T	0.014	0.67	0.11	1.87E-09
11	3328451	0	A/G	0.014	0.67	0.11	1.87E-09
11	3328726	0	G/A	0.014	0.67	0.11	1.87E-09
11	3329211	0	C/G	0.014	0.67	0.11	1.87E-09
11	3329505	0	T/C	0.014	0.67	0.11	1.87E-09
11	3329506	0	A/G	0.014	0.67	0.11	1.87E-09
11	3330361	0	A/C	0.014	0.67	0.11	1.87E-09
11	3331280	0	A/T	0.014	0.67	0.11	1.87E-09
11	3331281	0	A/G	0.014	0.67	0.11	1.87E-09
11	3332573	0	T/C	0.014	0.67	0.11	1.87E-09
11	3333643	0	C/T	0.014	0.67	0.11	1.87E-09
11	3333725	0	G/C	0.014	0.67	0.11	1.87E-09
11	3334068	0	G/C	0.014	0.67	0.11	1.87E-09
11	3334071	0	A/G	0.014	0.67	0.11	1.87E-09
11	3334197	0	A/C	0.014	0.67	0.11	1.87E-09
11	3334206	0	T/C	0.014	0.67	0.11	1.87E-09
11	3334209	0	T/C	0.014	0.67	0.11	1.87E-09
11	3334281	0	A/G	0.014	0.67	0.11	1.87E-09
11	3334328	0	A/T	0.014	0.67	0.11	1.87E-09
11	3334871	0	C/T	0.014	0.67	0.11	1.87E-09
11	3334872	0	T/C	0.014	0.67	0.11	1.87E-09
11	3334895	0	A/G	0.014	0.67	0.11	1.87E-09
11	3337798	0	A/T	0.014	0.67	0.11	1.87E-09
11	3939278	0	A/G	0.014	0.67	0.11	1.87E-09

11	3939347	0	T/C	0.014	0.67	0.11	1.87E-09
11	3939385	0	G/T	0.014	0.67	0.11	1.87E-09
11	3939540	0	G/A	0.014	0.67	0.11	1.87E-09
11	3940183	0	C/G	0.014	0.67	0.11	1.87E-09
11	3940200	0	C/T	0.014	0.67	0.11	1.87E-09
11	3940216	0	A/G	0.014	0.67	0.11	1.87E-09
11	3940234	0	T/C	0.014	0.67	0.11	1.87E-09
11	3940238	0	G/T	0.014	0.67	0.11	1.87E-09
11	3940266	0	C/T	0.014	0.67	0.11	1.87E-09
11	3940366	0	C/T	0.014	0.67	0.11	1.87E-09
11	3940380	0	T/C	0.014	0.67	0.11	1.87E-09
11	3940396	0	G/T	0.014	0.67	0.11	1.87E-09
11	3940509	0	T/C	0.014	0.67	0.11	1.87E-09
11	3940526	0	C/T	0.014	0.67	0.11	1.87E-09
11	3940544	0	C/T	0.014	0.67	0.11	1.87E-09
11	3940569	0	T/C	0.014	0.67	0.11	1.87E-09
11	3940580	0	A/G	0.014	0.67	0.11	1.87E-09
11	3940722	0	T/C	0.014	0.67	0.11	1.87E-09
11	3940860	0	T/C	0.014	0.67	0.11	1.87E-09
11	3940920	0	T/C	0.014	0.67	0.11	1.87E-09
11	3940943	0	C/T	0.014	0.67	0.11	1.87E-09
11	3940949	0	A/T	0.014	0.67	0.11	1.87E-09
11	3940986	0	A/G	0.014	0.67	0.11	1.87E-09
11	3941099	0	A/G	0.014	0.67	0.11	1.87E-09
11	3941172	0	A/T	0.014	0.67	0.11	1.87E-09
11	3941193	0	C/T	0.014	0.67	0.11	1.87E-09
11	3941338	0	C/T	0.014	0.67	0.11	1.87E-09
11	3941340	0	C/T	0.014	0.67	0.11	1.87E-09
11	3941360	0	G/A	0.014	0.67	0.11	1.87E-09
11	3941373	0	G/C	0.014	0.67	0.11	1.87E-09
11	3941408	0	G/A	0.014	0.67	0.11	1.87E-09
11	3941568	0	G/C	0.014	0.67	0.11	1.87E-09
11	3941954	0	G/C	0.014	0.67	0.11	1.87E-09
11	3941976	0	A/G	0.014	0.67	0.11	1.87E-09
11	3942055	0	G/A	0.014	0.67	0.11	1.87E-09
11	3942124	0	C/T	0.014	0.67	0.11	1.87E-09
11	3942179	0	G/A	0.014	0.67	0.11	1.87E-09
11	3942283	0	A/G	0.014	0.67	0.11	1.87E-09
11	3942340	0	T/C	0.014	0.67	0.11	1.87E-09
11	3942390	0	C/A	0.014	0.67	0.11	1.87E-09

11	3942419	0	A/G	0.014	0.67	0.11	1.87E-09
11	3942483	0	A/G	0.014	0.67	0.11	1.87E-09
11	3942522	0	C/T	0.014	0.67	0.11	1.87E-09
11	3942625	0	A/G	0.014	0.67	0.11	1.87E-09
11	3943012	0	T/C	0.014	0.67	0.11	1.87E-09
11	3943079	0	G/C	0.014	0.67	0.11	1.87E-09
11	3943100	0	A/G	0.014	0.67	0.11	1.87E-09
11	3943188	0	G/A	0.014	0.67	0.11	1.87E-09
11	3943204	0	T/A	0.014	0.67	0.11	1.87E-09
11	3943213	0	G/A	0.014	0.67	0.11	1.87E-09
11	3943248	0	T/A	0.014	0.67	0.11	1.87E-09
11	3943298	0	A/G	0.014	0.67	0.11	1.87E-09
11	3943342	0	T/C	0.014	0.67	0.11	1.87E-09
11	3943352	0	G/A	0.014	0.67	0.11	1.87E-09
11	3943444	0	G/A	0.014	0.67	0.11	1.87E-09
11	3943544	0	C/T	0.014	0.67	0.11	1.87E-09
11	3943573	0	A/G	0.014	0.67	0.11	1.87E-09
11	3943586	0	C/T	0.014	0.67	0.11	1.87E-09
11	3943627	0	C/T	0.014	0.67	0.11	1.87E-09
11	3943664	0	A/G	0.014	0.67	0.11	1.87E-09
11	3943665	0	A/C	0.014	0.67	0.11	1.87E-09
11	3943769	0	C/G	0.014	0.67	0.11	1.87E-09
11	3943783	0	A/C	0.014	0.67	0.11	1.87E-09
11	3943891	0	A/G	0.014	0.67	0.11	1.87E-09
11	3943937	0	C/T	0.014	0.67	0.11	1.87E-09
11	3944041	0	T/C	0.014	0.67	0.11	1.87E-09
11	3944138	0	C/T	0.014	0.67	0.11	1.87E-09
11	3944208	0	C/T	0.014	0.67	0.11	1.87E-09
11	3944231	0	T/C	0.014	0.67	0.11	1.87E-09
11	3944315	0	C/T	0.014	0.67	0.11	1.87E-09
11	3945588	0	T/C	0.014	0.67	0.11	1.87E-09
11	3946124	0	G/C	0.014	0.67	0.11	1.87E-09
11	3946485	0	A/G	0.014	0.67	0.11	1.87E-09
11	3947881	0	G/A	0.014	0.67	0.11	1.87E-09
11	3948094	0	T/C	0.014	0.67	0.11	1.87E-09
11	3948270	0	C/T	0.014	0.67	0.11	1.87E-09
11	3948953	0	G/A	0.014	0.67	0.11	1.87E-09
11	3949183	0	A/G	0.014	0.67	0.11	1.87E-09
11	3949488	0	G/C	0.014	0.67	0.11	1.87E-09
11	3989007	0	G/T	0.014	0.67	0.11	1.87E-09

11	3991530	0	A/G	0.014	0.67	0.11	1.87E-09
11	4044280	0	C/A	0.014	0.67	0.11	1.87E-09
11	4044502	0	T/C	0.014	0.67	0.11	1.87E-09
11	4044896	0	A/G	0.014	0.67	0.11	1.87E-09
11	4051291	0	A/G	0.014	0.67	0.11	1.87E-09
11	4052682	0	T/C	0.014	0.67	0.11	1.87E-09
11	4052762	0	A/G	0.014	0.67	0.11	1.87E-09
11	4053576	0	A/C	0.014	0.67	0.11	1.87E-09
11	4060138	0	C/T	0.014	0.67	0.11	1.87E-09
11	4061088	0	A/G	0.014	0.67	0.11	1.87E-09
11	4061128	0	C/G	0.014	0.67	0.11	1.87E-09
11	4061204	0	C/G	0.014	0.67	0.11	1.87E-09
11	4061208	0	G/A	0.014	0.67	0.11	1.87E-09
11	4061225	0	T/C	0.014	0.67	0.11	1.87E-09
11	4061360	0	C/T	0.014	0.67	0.11	1.87E-09
11	4066504	0	G/A	0.014	0.67	0.11	1.87E-09
11	4066697	0	A/G	0.014	0.67	0.11	1.87E-09
11	4067148	0	T/C	0.014	0.67	0.11	1.87E-09
11	4073881	0	A/T	0.014	0.67	0.11	1.87E-09
11	4074015	0	A/G	0.014	0.67	0.11	1.87E-09
11	2719869	0	T/C	0.011	0.75	0.12	2.02E-09
11	2719880	0	G/T	0.011	0.75	0.12	2.02E-09
11	2720343	0	T/C	0.011	0.75	0.12	2.02E-09
11	2720899	0	G/A	0.011	0.75	0.12	2.02E-09
11	2722217	0	G/C	0.011	0.75	0.12	2.02E-09
11	2723290	0	A/C	0.011	0.75	0.12	2.02E-09
11	2724004	0	C/G	0.011	0.75	0.12	2.02E-09
11	2724053	0	A/G	0.011	0.75	0.12	2.02E-09
11	2724268	0	G/A	0.011	0.75	0.12	2.02E-09
11	2724321	0	T/C	0.011	0.75	0.12	2.02E-09
11	2724367	0	C/T	0.011	0.75	0.12	2.02E-09
11	2724506	0	T/G	0.011	0.75	0.12	2.02E-09
11	2724560	0	T/C	0.011	0.75	0.12	2.02E-09
11	2724812	0	A/G	0.011	0.75	0.12	2.02E-09
11	2724923	0	G/A	0.011	0.75	0.12	2.02E-09
11	2725592	0	C/T	0.011	0.75	0.12	2.02E-09
11	2725697	0	G/A	0.011	0.75	0.12	2.02E-09
11	2726312	0	C/T	0.011	0.75	0.12	2.02E-09
11	2726451	0	A/G	0.011	0.75	0.12	2.02E-09
11	2727077	0	G/C	0.011	0.75	0.12	2.02E-09

11	2727128	0	A/G	0.011	0.75	0.12	2.02E-09
11	2728233	0	T/C	0.011	0.75	0.12	2.02E-09
11	2728363	0	A/G	0.011	0.75	0.12	2.02E-09
11	2728417	0	A/G	0.011	0.75	0.12	2.02E-09
11	2728469	0	T/C	0.011	0.75	0.12	2.02E-09
11	2729317	0	G/C	0.011	0.75	0.12	2.02E-09
11	2729480	0	A/G	0.011	0.75	0.12	2.02E-09
11	2729860	0	A/G	0.011	0.75	0.12	2.02E-09
11	2730002	0	A/G	0.011	0.75	0.12	2.02E-09
11	2730005	0	A/G	0.011	0.75	0.12	2.02E-09
11	2730007	0	G/T	0.011	0.75	0.12	2.02E-09
11	2730010	0	T/C	0.011	0.75	0.12	2.02E-09
11	2730015	0	G/A	0.011	0.75	0.12	2.02E-09
11	2730017	0	T/G	0.011	0.75	0.12	2.02E-09
11	2730023	0	A/G	0.011	0.75	0.12	2.02E-09
11	2730027	0	A/G	0.011	0.75	0.12	2.02E-09
11	2730029	0	T/C	0.011	0.75	0.12	2.02E-09
11	2730035	0	T/C	0.011	0.75	0.12	2.02E-09
11	2730036	0	G/A	0.011	0.75	0.12	2.02E-09
11	2730038	0	G/A	0.011	0.75	0.12	2.02E-09
11	2730043	0	T/A	0.011	0.75	0.12	2.02E-09
11	2730047	0	C/T	0.011	0.75	0.12	2.02E-09
11	2731741	0	T/A	0.011	0.75	0.12	2.02E-09
11	2732074	0	A/G	0.011	0.75	0.12	2.02E-09
11	2732695	0	A/G	0.011	0.75	0.12	2.02E-09
11	2733394	0	T/C	0.011	0.75	0.12	2.02E-09
11	2733987	0	A/T	0.011	0.75	0.12	2.02E-09
11	2734201	0	A/G	0.011	0.75	0.12	2.02E-09
11	2734353	0	A/C	0.011	0.75	0.12	2.02E-09
11	2734365	0	A/G	0.011	0.75	0.12	2.02E-09
11	2734393	0	A/G	0.011	0.75	0.12	2.02E-09
11	2734545	0	G/C	0.011	0.75	0.12	2.02E-09
11	2734824	0	T/C	0.011	0.75	0.12	2.02E-09
11	2735403	0	G/A	0.011	0.75	0.12	2.02E-09
11	2735489	0	T/C	0.011	0.75	0.12	2.02E-09
11	2735490	0	G/A	0.011	0.75	0.12	2.02E-09
11	2735512	0	C/G	0.011	0.75	0.12	2.02E-09
11	2735562	0	A/G	0.011	0.75	0.12	2.02E-09
11	2735609	0	A/C	0.011	0.75	0.12	2.02E-09
11	2735623	0	T/C	0.011	0.75	0.12	2.02E-09

11	2735675	0	G/A	0.011	0.75	0.12	2.02E-09
11	2736501	0	T/C	0.011	0.75	0.12	2.02E-09
11	2739929	0	G/C	0.011	0.75	0.12	2.02E-09
11	2739936	0	A/C	0.011	0.75	0.12	2.02E-09
11	2740142	0	C/T	0.011	0.75	0.12	2.02E-09
11	2740152	0	A/G	0.011	0.75	0.12	2.02E-09
11	2740160	0	T/G	0.011	0.75	0.12	2.02E-09
11	2740643	0	G/A	0.011	0.75	0.12	2.02E-09
11	2741067	0	C/A	0.011	0.75	0.12	2.02E-09
11	2741419	0	T/C	0.011	0.75	0.12	2.02E-09
11	2741603	0	A/G	0.011	0.75	0.12	2.02E-09
11	2742807	0	C/T	0.011	0.75	0.12	2.02E-09
11	2742813	0	C/G	0.011	0.75	0.12	2.02E-09
11	2743001	0	T/C	0.011	0.75	0.12	2.02E-09
11	2744002	0	A/G	0.011	0.75	0.12	2.02E-09
11	2744394	0	C/T	0.011	0.75	0.12	2.02E-09
11	2744581	0	T/C	0.011	0.75	0.12	2.02E-09
11	2744585	0	C/T	0.011	0.75	0.12	2.02E-09
11	2744595	0	A/G	0.011	0.75	0.12	2.02E-09
11	2744655	0	C/T	0.011	0.75	0.12	2.02E-09
11	2744661	0	T/C	0.011	0.75	0.12	2.02E-09
11	2744667	0	T/C	0.011	0.75	0.12	2.02E-09
11	2748864	0	G/T	0.011	0.75	0.12	2.02E-09
11	2751907	0	G/A	0.011	0.75	0.12	2.02E-09
11	2751908	0	G/A	0.011	0.75	0.12	2.02E-09
11	2752118	0	A/T	0.011	0.75	0.12	2.02E-09
11	2752143	0	A/G	0.011	0.75	0.12	2.02E-09
11	2752271	0	G/A	0.011	0.75	0.12	2.02E-09
11	2752304	0	G/A	0.011	0.75	0.12	2.02E-09
11	2753088	0	G/C	0.011	0.75	0.12	2.02E-09
11	2753113	0	A/G	0.011	0.75	0.12	2.02E-09
11	2753119	0	C/T	0.011	0.75	0.12	2.02E-09
11	2753171	0	G/T	0.011	0.75	0.12	2.02E-09
11	2753383	0	A/G	0.011	0.75	0.12	2.02E-09
11	2753389	0	G/C	0.011	0.75	0.12	2.02E-09
11	2753404	0	A/G	0.011	0.75	0.12	2.02E-09
11	2753415	0	T/C	0.011	0.75	0.12	2.02E-09
11	2753513	0	G/A	0.011	0.75	0.12	2.02E-09
11	2753602	0	G/A	0.011	0.75	0.12	2.02E-09
11	2753785	0	T/C	0.011	0.75	0.12	2.02E-09

11	2753787	0	T/G	0.011	0.75	0.12	2.02E-09
11	2753843	0	A/G	0.011	0.75	0.12	2.02E-09
11	2753844	0	G/A	0.011	0.75	0.12	2.02E-09
11	2753944	0	A/G	0.011	0.75	0.12	2.02E-09
11	2754047	0	C/T	0.011	0.75	0.12	2.02E-09
11	2754068	0	T/C	0.011	0.75	0.12	2.02E-09
11	2754107	0	G/C	0.011	0.75	0.12	2.02E-09
11	2754280	0	C/A	0.011	0.75	0.12	2.02E-09
11	2754289	0	G/A	0.011	0.75	0.12	2.02E-09
11	2754329	0	A/G	0.011	0.75	0.12	2.02E-09
11	2754356	0	C/T	0.011	0.75	0.12	2.02E-09
11	2754357	0	A/G	0.011	0.75	0.12	2.02E-09
11	2754365	0	G/C	0.011	0.75	0.12	2.02E-09
11	2754379	0	C/T	0.011	0.75	0.12	2.02E-09
11	2754460	0	A/G	0.011	0.75	0.12	2.02E-09
11	2754642	0	T/C	0.011	0.75	0.12	2.02E-09
11	2754830	0	A/G	0.011	0.75	0.12	2.02E-09
11	2755084	0	A/G	0.011	0.75	0.12	2.02E-09
11	2755188	0	T/C	0.011	0.75	0.12	2.02E-09
11	2765561	0	T/G	0.011	0.75	0.12	2.02E-09
11	2765564	0	A/C	0.011	0.75	0.12	2.02E-09
11	2765738	0	C/G	0.011	0.75	0.12	2.02E-09
11	2765745	0	A/C	0.011	0.75	0.12	2.02E-09
11	2765755	0	A/G	0.011	0.75	0.12	2.02E-09
11	2765774	0	A/G	0.011	0.75	0.12	2.02E-09
11	2765850	0	T/C	0.011	0.75	0.12	2.02E-09
11	2766008	0	C/A	0.011	0.75	0.12	2.02E-09
11	2766388	0	T/C	0.011	0.75	0.12	2.02E-09
11	2796718	0	T/C	0.011	0.75	0.12	2.02E-09
11	2802414	0	T/C	0.011	0.75	0.12	2.02E-09
11	2802456	0	T/C	0.011	0.75	0.12	2.02E-09
11	2819045	0	G/A	0.011	0.75	0.12	2.02E-09
11	2878273	0	T/C	0.011	0.75	0.12	2.02E-09
11	2878277	0	A/G	0.011	0.75	0.12	2.02E-09
11	2878279	0	T/A	0.011	0.75	0.12	2.02E-09
11	2878876	0	C/T	0.011	0.75	0.12	2.02E-09
11	2888502	0	C/G	0.011	0.75	0.12	2.02E-09
11	2904518	0	T/C	0.011	0.75	0.12	2.02E-09
11	2904528	0	A/G	0.011	0.75	0.12	2.02E-09
11	2904561	0	G/A	0.011	0.75	0.12	2.02E-09

11	2904575	0	T/C	0.011	0.75	0.12	2.02E-09
11	2904576	0	G/A	0.011	0.75	0.12	2.02E-09
11	2904593	0	T/C	0.011	0.75	0.12	2.02E-09
11	2904599	0	C/T	0.011	0.75	0.12	2.02E-09
11	2904608	0	C/T	0.011	0.75	0.12	2.02E-09
11	2904652	0	T/C	0.011	0.75	0.12	2.02E-09
11	2904656	0	A/G	0.011	0.75	0.12	2.02E-09
11	2616942	0	A/G	0.017	0.62	0.10	2.13E-09
11	103293	0	A/T	0.025	0.42	0.07	2.39E-09
11	119942	0	T/C	0.025	0.42	0.07	2.39E-09
11	128696	0	C/T	0.025	0.42	0.07	2.39E-09
11	842707	0	C/G	0.025	0.42	0.07	2.39E-09
11	887777	0	A/T	0.025	0.42	0.07	2.39E-09
11	887778	0	A/G	0.025	0.42	0.07	2.39E-09
11	905638	0	T/A	0.025	0.42	0.07	2.39E-09
11	905772	0	C/G	0.025	0.42	0.07	2.39E-09
11	912192	0	G/C	0.025	0.42	0.07	2.39E-09
11	913445	0	G/C	0.025	0.42	0.07	2.39E-09
11	913550	0	G/A	0.025	0.42	0.07	2.39E-09
11	913708	0	C/T	0.025	0.42	0.07	2.39E-09
11	913717	0	A/G	0.025	0.42	0.07	2.39E-09
11	915618	0	A/G	0.025	0.42	0.07	2.39E-09
11	917018	0	A/G	0.025	0.42	0.07	2.39E-09
11	921628	0	T/C	0.025	0.42	0.07	2.39E-09
11	928412	0	C/T	0.025	0.42	0.07	2.39E-09
11	930839	0	C/T	0.025	0.42	0.07	2.39E-09
11	934383	0	C/T	0.025	0.42	0.07	2.39E-09
11	937063	0	A/G	0.025	0.42	0.07	2.39E-09
11	997029	0	T/C	0.025	0.42	0.07	2.39E-09
11	998032	0	T/C	0.025	0.42	0.07	2.39E-09
11	1005275	0	A/G	0.025	0.42	0.07	2.39E-09
11	1006026	0	T/C	0.025	0.42	0.07	2.39E-09
11	1012321	0	C/T	0.025	0.42	0.07	2.39E-09
11	1015794	0	C/G	0.025	0.42	0.07	2.39E-09
11	1019416	0	T/C	0.025	0.42	0.07	2.39E-09
11	1020301	0	T/C	0.025	0.42	0.07	2.39E-09
11	1023305	0	A/G	0.025	0.42	0.07	2.39E-09
11	1023895	0	A/G	0.025	0.42	0.07	2.39E-09
11	1192994	0	T/G	0.025	0.42	0.07	2.39E-09
11	1206766	0	A/G	0.025	0.42	0.07	2.39E-09

11	1207379	0	A/T	0.025	0.42	0.07	2.39E-09
11	1208824	0	G/A	0.025	0.42	0.07	2.39E-09
11	1213881	0	T/G	0.025	0.42	0.07	2.39E-09
11	1270119	0	T/C	0.025	0.42	0.07	2.39E-09
11	1300991	0	C/T	0.025	0.42	0.07	2.39E-09
11	1309052	0	A/G	0.025	0.42	0.07	2.39E-09
11	1313471	0	T/G	0.025	0.42	0.07	2.39E-09
11	1339354	0	A/G	0.025	0.42	0.07	2.39E-09
11	1342650	0	A/G	0.025	0.42	0.07	2.39E-09
11	1344486	0	T/C	0.025	0.42	0.07	2.39E-09
11	1351965	0	G/A	0.025	0.42	0.07	2.39E-09
11	1390601	0	T/C	0.025	0.42	0.07	2.39E-09
11	1404952	0	G/C	0.025	0.42	0.07	2.39E-09
11	1407695	0	T/C	0.025	0.42	0.07	2.39E-09
11	1268796	0	G/C	0.075	0.28	0.05	3.55E-09
11	1268800	0	C/G	0.075	0.28	0.05	3.55E-09
11	1268816	0	A/G	0.075	0.28	0.05	3.55E-09
11	1269864	0	T/G	0.075	0.28	0.05	3.55E-09
11	1270189	0	T/C	0.075	0.28	0.05	3.55E-09
11	1270274	0	A/G	0.075	0.28	0.05	3.55E-09
11	1270709	0	A/G	0.075	0.28	0.05	3.55E-09
11	1270944	0	T/A	0.075	0.28	0.05	3.55E-09
11	1272666	0	A/C	0.075	0.28	0.05	3.55E-09
11	1272673	0	G/C	0.075	0.28	0.05	3.55E-09
11	1272828	0	A/C	0.075	0.28	0.05	3.55E-09
11	1272829	0	A/G	0.075	0.28	0.05	3.55E-09
11	1273061	0	A/T	0.075	0.28	0.05	3.55E-09
11	1182762	0	T/C	0.031	0.38	0.06	7.62E-09
11	1190749	0	G/C	0.031	0.38	0.06	7.62E-09
11	1276133	0	T/C	0.031	0.38	0.06	7.62E-09
11	1279668	0	A/G	0.031	0.38	0.06	7.62E-09
11	1292722	0	A/G	0.031	0.38	0.06	7.62E-09
11	1292785	0	T/C	0.031	0.38	0.06	7.62E-09
11	1293435	0	T/C	0.031	0.38	0.06	7.62E-09
11	1298380	0	T/C	0.031	0.38	0.06	7.62E-09
11	1146340	0	A/C	0.067	0.29	0.05	8.26E-09
