# Supplementary material for: Whole-genome sequence association study identifies cyclin dependent kinase 8 as a key gene for the number of mummified piglets
Source: Anim Biosci. 2022 Sep 7;36(1):29–42. doi: 10.5713/ab.22.0115 (PMC9834657; doi:10.5713/ab.22.0115)
Supplement: Supplementary file 7 [file ab-22-0115-suppl7.pdf]

Supplemental Table S7. Summary of transcription factor information

Gene	Number of proteins	Peptides	Sequence coverage (%)	Unique sequence coverage (%)	Q-value	Score	iBAQ
YBX1,YBX2, CSD	11	9	35.5	24.9	0	127	13207000
PURB,PURG	7	8	26.3	26.3	0	81.679	55275000
LYAR	4	4	15.2	15.2	0	53.52	7716400
UBTF	7	4	7.6	7.6	0	30.296	306830
MBD1	8	3	5.8	5.8	0	27.208	558080
IQGAP1	4	3	2.2	2.2	0	18.341	166720
CREB1,ATF1,CREM	13	2	8.3	8.3	0	17.976	2892000
CNBP	3	1	8.2	8.2	0	16.815	368960
PC4	4	2	23.6	23.6	0	16.678	2194100
YBX3	4	5	18.9	6.1	0	13.6	800400
TFAM	2	2	11.4	11.4	0	13.386	735120
SAND	5	2	2.3	2.3	0	11.694	66464

SON	4	1	0.5	0.5	0.006079	6.1525	91591
ZNF236	4	1	0.6	0.6	0.006042	6.1344	0
PHB	3	1	4.2	4.2	0.005865	6.0171	0
CLOCK	6	1	1.5	1.5	0.008721	5.9882	0
